# Stereocalpin B, a New Cyclic Depsipeptide from the Antarctic Lichen *Ramalina terebrata*

**DOI:** 10.3390/metabo12020141

**Published:** 2022-02-03

**Authors:** Seulah Lee, Se Yun Jeong, Dieu Linh Nguyen, Jae Eun So, Ki Hyun Kim, Ji Hee Kim, Se Jong Han, Sung-Suk Suh, Jun Hyuck Lee, Ui Joung Youn

**Affiliations:** 1 Division of Life Sciences, Korea Polar Research Institute, Incheon 21990, Korea; seulah@kopri.re.kr (S.L.); ndlinh@kopri.re.kr (D.L.N.); cladonia@kopri.re.kr (J.E.S.); jhalgae@kopri.re.kr (J.H.K.); hansj@kopri.re.kr (S.J.H.); 2 School of Pharmacy, Sungkyunkwan University, Suwon 16419, Korea; jseyun12@gmail.com (S.Y.J.); khkim83@skku.edu (K.H.K.); 3 Department of Polar Sciences, University of Science and Technology, Incheon 21990, Korea; junhyucklee@kopri.re.kr; 4 Department of Bioscience, Mokpo National University, Mokpo 58554, Korea; sungsuksuh@mokpo.ac.kr; 5 Research Unit of Cryogenic Novel Material, Korea Polar Research Institute, Incheon 21990, Korea

**Keywords:** *Ramalina terebrata*, cyclic depsipeptides, dibenzofuran, antimicrobial, cytotoxicity, anti-inflammation

## Abstract

Stereocalpin B, a new cyclic depsipeptide (**1**), and a new dibenzofuran derivative (**3**), were isolated from the Antarctic lichen, *Ramalina terebrata* (Ramalinaceae), along with a known cyclic depsipeptide (**2**). The structures of new compounds were characterized by comprehensive spectrometric analyses; high-resolution fast atom bombardment mass spectrometry (HR-FABMS) and liquid chromatography-tandem mass spectrometry (LC-MS/MS). Stereocalpin B (**1**) existed in a rotameric equilibrium, which was confirmed using nuclear Overhauser effect spectroscopy (NOESY)/exchange spectroscopy (EXSY) spectrum. Absolute configurations of the amino acid units in **1** were assigned using the advanced Marfey’s method and subsequent NOESY analysis of the 5-hydroxy-2,4-dimethyl-3-oxo-decanoic acid residue confirmed the complete stereochemistry of **1**. Compounds **1-3** exhibited moderate antimicrobial activities against *E. coli*, with the IC_50_ values ranging from 18–30 μg/mL. Compound **2** exhibited cell growth inhibition against HCT116 cell lines, with the IC_50_ value of 20 ± 1.20 μM, and compounds **1** and **2** also showed potent anti-inflammatory activities against lipopolysaccharide (LPS)-induced RAW264.7 macrophages with the IC_50_ values ranging from 5–7 μM.

## 1. Introduction

Lichens, which are also referred to as lichenized fungi, are mutualistic symbionts involving microalga and/or cyanobacterium and fungus [1]. They are known to possess a broad variety of secondary metabolites with diverse biological activities, which are reported to be useful for the treatment of heart diseases, bronchitis, bleeding pile, vomiting, asthma, inflammation, and stomach disorders [1,2]. However, only a limited number of study has been carried out for the discovery of medicinal use and therapeutic potential of lichen-derived substances [3]. Lichens are well known for their composition of depsides and dibenzofurans as major secondary metabolites, where depsides are reported to have antioxidant and antifungal activities [2,4], and dibenzofurans are known to exhibit anticancer, antifungal, and antineoplastic properties [5,6,7]. The Antarctic lichen, *Ramalina terebrata*, is also known to possess various secondary metabolites such as usnic acid derivatives, ramalin, and anthraquinones [1,8,9,10]. Derived from *R. terebrata*, usnic acid, and usimines A–C were reported to have antibacterial activity against *B. subtilis* [11], ramalin to have anti-inflammatory, anticancer, and antioxidant activity [9,12], and parietin was shown to serve as both anti-aggregative and antioxidant agents in tauopathies [10,13].

In our continuous search for novel bioactive secondary metabolites from the Antarctic lichens, the acetone extract of *R. terebrata* was subjected to chemical investigation. Here, the identification of the unknown compound **1**, stereocalpin B, is reported along with compound **3,** which has been isolated as a natural product for the first time, together with a known cyclic depsipeptide (**2**). The structure elucidation of the new compounds (**1** and **3**) and the biological activities of all isolated compounds (**1**–**3**) are discussed below.

## 2. Results and Discussion

### 2.1. Isolation of Compounds ***1***–***3***

**Figure 1:**
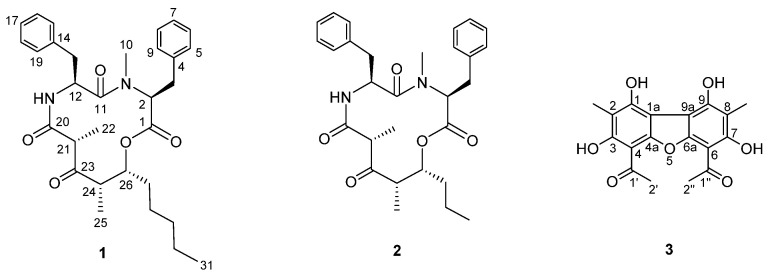
Structures of compounds **1**–**3** isolated from *R. terebrata*.

Air-dried and powdered lichen, *R. terebrata*, was extracted with acetone which provided the resultant acetone extract. The extract was subjected to fractionation and isolation, where column chromatography and HPLC purification techniques were used, resulting in the isolation of a new cyclic depsipeptide, stereocalpin B (**1**), and a new dibenzofuran derivative, 1,3,7,9-tetrahydroxy-2,8-dimethyl-4,6-di(ethanoyl)dibenzofuran (**3**), along with a known cyclic depsipeptide (**2**) (Figure 1). Compound **2** was identified to be stereocalpin A (**2**) [14] by comparing its NMR spectroscopic and physical data with those previously reported.

### 2.2. Structure Elucidation of Stereocalpin B (***1***)

Stereocalpin B (**1**) was isolated as a white powder. The molecular formula was determined to be C_31_H_40_N_2_O_5_ from the molecular ion peak [M + H]^+^ at *m/z* 521.3017 (calculated for C_31_H_41_N_2_O_5_, 521.3015) in the positive-ion mode of HR-FABMS. The IR spectrum showed absorption bands for N-H (3297 cm^−1^), C-H (2933 cm^−1^), C=O (1742 cm^−1^), and aromatic C-H (1653 cm^−1^) vibrations (Supplementary Material Figure S3). The ^1^H NMR spectrum of **1** (Table 1) showed signals that are similar to those of stereocalpin A (**2**) [14], isolated in the current study. The characteristic signals include two α-amino protons (δ_H_ 4.90 (1H, dd, *J* = 11.0, 4.6 Hz), 5.08 (1H, ddd, *J* = 9.5, 8.0, 6.6 Hz)), four methyl groups (δ_H_ 0.85 (3H, t, *J* = 6.7 Hz), 0.93 (3H, d, *J* = 6.6 Hz), 1.04 (3H, d, *J* = 7.1 Hz), 3.06 (3H, s)), and overlapped signals attributable to aromatic protons in the range of δ_H_ 6.60–7.39. The ^13^C NMR data of **1** (Table 1) acquired by the assistance of HSQC spectrum supported that compound **1** possesses two α-amino carbons (δ_C_ 50.5, 59.5), four methyl carbons (δ_C_ 13.6, 13.9, 14.3, 29.5), and overlapping signals between δ_C_ 126.2–136.5 attributable to the benzene rings.

**Figure 2:**
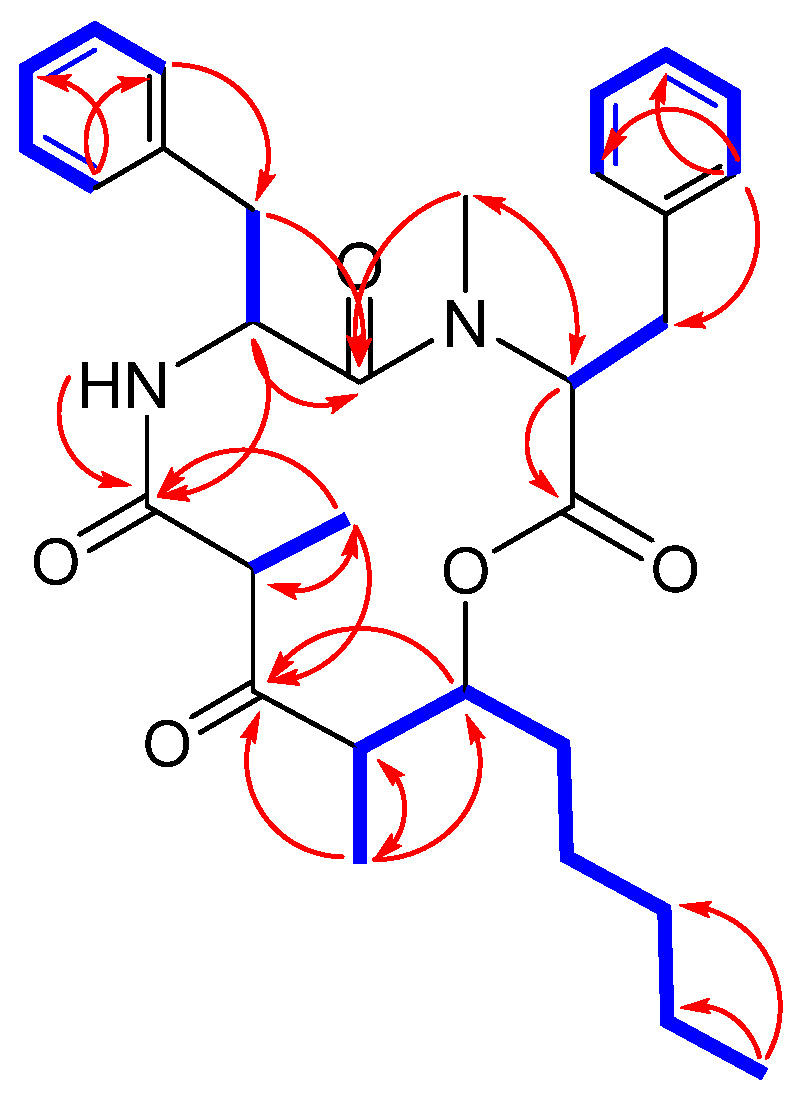
Key HMBC (red arrows) and ^1^H-^1^H COSY (bold blue lines) correlations of **1**.

By analyzing 2D NMR spectra, the structure of compound **1** was determined to be only different in the length of the carbon side chain (C27–C31) from stereocalpin A (C27–C29) [14], which was supported by the observed spin systems from C-24–C-26–C-27–C-28–C-29–C-30–C-31 in ^1^H-^1^H correlation spectroscopy (COSY) spectrum (Figure 2), consisting of Phe and *N*-Me-Phe residues as the amino acid sequences. The assignments were further confirmed by LC-MS/MS spectra, where fragment ions at *m/z* 342 (Phe−5,6-dehydro-2,4-dimethyl-3-oxo-decanoic acid), *m/z* 195 (5,6-dehydro-2,4-dimethyl-3-oxo-decanoic acid), *m/z* 180 (*N*-Me-Phe), and *m/z* 134 (*N*-Me-2-phenylethan-1-ylium-1-aminium) were detected, indicating the sequential cleavage of *N*-Me-Phe−Phe−5-hydroxy-2,4-dimethyl-3-oxo-decanoic acid (Figure S9).

Stereocalpin B (**1**) appeared to exist as HPLC inseparable mixture, and careful analysis of 2D NMR spectra revealed that **1** was either a mixture of diastereomers or equilibrating rotamers. The ^1^H and 2D NMR spectra gave sufficient information that **1** was a mixture of inseparable equilibrating rotamers, **1a** and **1b**, and the two sets of resonances existed with an intensity ratio of 1:1. This was further supported by the correlation signals observed in NOESY spectrum of **1**, using a technique also referred to as the exchange spectroscopy (EXSY) [15]. The NOESY/EXSY spectrum showed NOE correlations between the rotameric signals, for example, H-2 in **1a** with H-2 in **1b**, and H-26 in **1a** with H-26 in **1b** (Figure S10).

**Figure 3:**
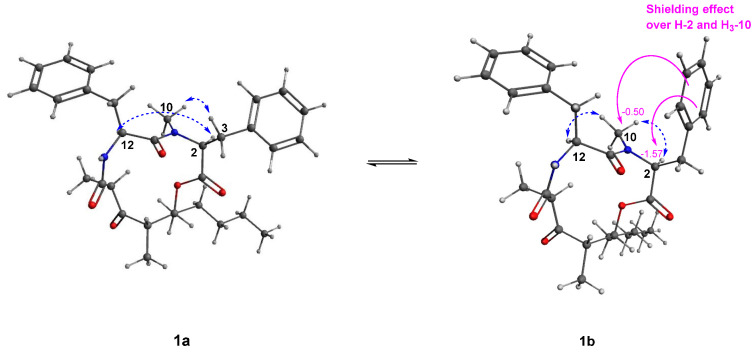
Key NOESY (blue dotted arrows) correlations of **1a** and **1b** and anisotropic shielding effect in **1b**.

The absolute configurations of the amino acid units were assigned using the advanced Marfey’s method [16,17]. LC-MS analysis of the acid hydrolysate of **1** revealed that the amino acid residues were L-Phe and *N*-Me-L-Phe, by comparing with the amino acid standards treated with Marfey’s reagent (1-fluoro-2-4-dinitrophenyl-5-L-alanine amide, FDAA) (Figure S11). Further study to assign the absolute configuration of the rest of the structure, 5-hydroxy-2,4-dimethyl-3-oxo-decanoic acid residue, was proposed by NOESY analysis. The nuclear Overhauser effects (NOEs) were observed between NH/H-13β, NH/H-21, H-21/H_3_-25, and H_3_-22/H-24, as reported for stereocalpin A by Seo et al. in 2008 [14]. The reported absolute configuration at C-21 for naturally occurring stereocalpin A is *R*; however, stereocalpin A possesses a potentially labile α-methyl β-keto carboxamide, where C-22 methyl functionality is capable of experiencing configurational change and converts into the 21-*epi* form via the enol (enolate) forms E1 and/or E2 during purification processes (Figure S12), clarifying the misleading NOE correlations of H-21 and H_3_-22 [18]. This also implies the directions of C-22 and C-25 methyl groups toward the opposite sides, from the macrocycle ring [18]. NOEs were also observed between H-24/H-26, H_3_-25/H-26, and H_3_-25/H_2_-27 posing difficulty in corroborating the stereochemistry at C-24 and C-26. Instead, vicinal *J* coupling constants between H-24 and H-26 were indicative of cis configuration (^3^*J*_H-24/H-26_ = 3.6 Hz in **1a** and 2.7 Hz in **1b**), suggesting close proximity of the two protons with a small dihedral angle [18]. Due to the conformational flexibility of the macrocyclic nature of **1**, we could not obtain suitable ECD calculations, as flexible natural products are known to have a large number of stable conformers [19]. The conformational differences of the rotamers were observable in the NOESY spectrum (Figure 3), and careful analysis of the spectrum revealed that rotamers **1a** and **1b** share the same configurations of the cyclic depsipeptide backbone, but significant differences were observed due to conformational changes. Prominent differences were the observation of NOEs between H-2/H-12 and H-3a/H_3_-10 for the conformational isomer **1a**, while they were not observed in case of **1b**. Instead, correlations between H-2/H_3_-10 and H_3_-10/H-12 were observed in **1b** (Figure 3). Based on these data, an estimation could be made that free rotation around the C-2–C-3 and C-3–C-4 bonds caused conformational changes affecting the interproton distances between neighboring protons. We expected that principal distinctions that would have been made due to this rotation would be distances between H-3/H-2 and H-3/H_3_-10, where calibrated interproton distances between H-3a/H-2 and H-3a/H_3_-10, calculated using NOE-based interproton distance measurement technique via the peak amplitude normalization for improved cross-relaxation (PANIC) method [20,21,22,23,24], were 2.48 Å and 2.50 Å in **1a**, respectively (Figures S13–S17). To predict the corresponding interproton distances in **1a** and **1b**, molecular mechanics (MM) and quantum mechanics (QM)-optimized 3D structures of each rotamer were generated, where the predicted values were 2.83 Å and 3.06 Å for H-3a/H-2, and 3.59 Å and 4.36 Å for H-3a/H_3_-10 in **1a** and **1b**, respectively. Although there was a discrepancy in calculated/predicted interproton distance between H-3a/H_3_-10, the calculated values were closer to that of **1a**, and the difference in predicted values of each rotamer also confirmed the occurrence of free rotation around the C-2–C-3 and C-3–C-4 bonds. The calculated interproton distances of H-2/H_3_-10 and H_3_-10/H-12 were 2.33 Å and 2.18 Å in **1b**, respectively (Figures S16 and S17), implying medium-strong NOEs [21].

Additionally, a noticeable disparity in the chemical shifts was observed, where chemical shifts for H-2 were δ_H_ 4.90 in **1a,** δ_H_ 3.33 in **1b**, and ^13^C chemical shifts were δ_C_ 59.5 and δ_C_ 67.3, respectively. This appeared to be caused by an anisotropic shielding effect of the benzene ring in **1b**, generating upfield shift for H-2 as compared with the corresponding proton chemical shift in **1a** (Figure 3) [25,26], as free rotation occurs around the C-2–C-3 and C-3–C-4 bonds. *N*-methyl group also seemed to be exposed to the shielding effect, where ^1^H and ^13^C chemical shifts were δ_H_ 3.06 and δ_C_ 29.5 in **1a**, and δ_H_ 2.56 and δ_C_ 39.4 in **1b** (Figure 3). Based on the above intensive structural analysis, the complete structure of **1** was determined as shown in Figure 1 and named stereocalpin B.

### 2.3. Structure Elucidation of Compound ***3***

1,3,7,9-Tetrahydroxy-2,8-dimethyl-4,6-di(ethanoyl)dibenzofuran (**3**) was isolated as yellow powder. The molecular formula was established as C_18_H_16_O_7_ from the molecular ion peak [M + H]^+^ at *m/z* 345.0972 (calcd. for C_18_H_17_O_7_, 345.0974) in the positive-ion mode of HR-FABMS. The IR spectrum showed absorption bands of hydroxyl (3370 cm^−1^), carbonyl (1697 cm^−1^) and aromatic (1632 cm^−1^) functional groups (Figure S20). The ^1^H NMR spectrum of **3** (Table 2) showed signals for two deshielded methyl groups at δ_H_ 1.99 (3H, s) and 2.66 (3H, s), and a chelated hydroxyl group at δ_H_ 13.37 (1H, s). The ^13^C NMR data (Table 2) showed only 9 carbon signals comprised of two methyl groups, a carbonyl group, oxygenated and quaternary aromatic carbons, which suggested that compound **3** is symmetric.

**Figure 4:**
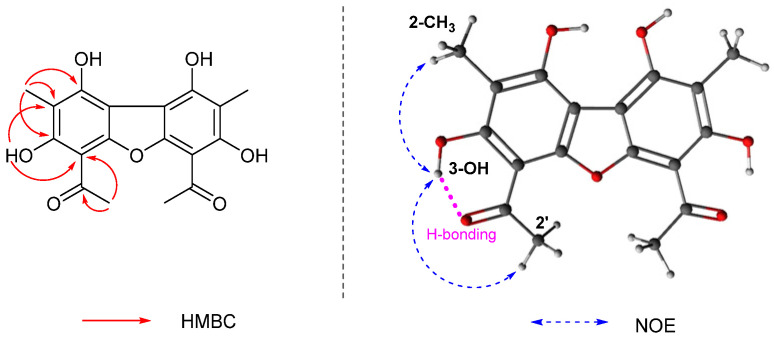
Key HMBC (red arrows) and NOE (blue dotted arrows) correlations of **3**.

The HMBC correlations of H_3_-2′/C-1′ (δ_C_ 200.8), H_3_-2′/C-4 (δ_C_ 101.0), 3-OH/C-4, 3-OH/C-2 (δ_C_ 106.9), CH_3_-2/C-2, CH_3_-2/C-1 (δ_C_ 157.0) and CH_3_-2/C-3 completed the partial structure of compound **3** (Figure 4). Based on the evidence that **3** had a symmetric structure, its spectroscopic data were compared with those previously reported, and it was found that compound **3** was structurally similar with 1,3,7,9-tetrahydroxy-2,8-dimethyl-4,6-di(2-methylbutanoyl)dibenzofuran and 1,3,7,9-tetrahydroxy-2,8-dimethyl-4,6-di(2-methylpropionyl)dibenzofuran [27]. The only difference was the side chains at C-4 and C-6, where the acetyl group was present in the case of **3**. The hydroxyl groups attached at C-3 and C-7 (δ_H_ 13.37) appeared to participate in a strong intermolecular hydrogen bond, each with OC-1′ and OC-1′’ groups, respectively. The cross-peaks between 3-OH/H_3_-2′, and 3-OH/2-CH_3_ in NOESY spectrum further supported the structure (Figure 4), deducing **3** to be 1,3,7,9-tetrahydroxy-2,8-dimethyl-4,6-di(ethanoyl)dibenzofuran. Compound **3** was registered in SciFinder with CAS registry number 674786-23-3; however, it has not been isolated as a natural product, and this is the first study to describe **3** in terms of its chemical nature.

### 2.4. Biological Activities of the Isolated Compounds (***1***–***3***)

All isolated compounds were evaluated for antimicrobial activities against *Escherichia coli*, *Staphylococcus aureus*, *Klebsiella pneumoniae*, *Candida albicans*, and *Mycobacterium smegmatis*. The tested compounds (**1**–**3**) only showed significant activity against *E. coli*, where compounds **1–3** exhibited moderate inhibition with the IC_50_ values of 30, 28, and 18 μg/mL, respectively, compared to the positive control (Apramycin, IC_50_ = 2 μg/mL) (Table S1). Compounds **1**–**3** were also tested for their cytotoxicity and anti-inflammatory activity. Compound **2** exhibited cytotoxicity against HCT116 cells, human colorectal carcinoma cell lines, with the IC_50_ value of 20 ± 1.20 μM (Table S2). Compounds **1** and **2** showed strong inhibition on NO production in LPS-induced RAW264.7 macrophages, in which the IC_50_ values were 7 ± 0.004 μM and 5 ± 0.006 μM, respectively. Stereocalpins are a unique class of depsipeptides derived from the Antarctic lichens, having only stereocalpin A being previously reported. Only small groups of dibenzofurans have been reported thus far, and the most studied type is usnic acid, adding uniqueness to the identification of compound **3** as another type of dibenzofuran isolated from the Antarctic lichen. Based on these findings, it was rationally concluded that stereocalpins and dibenzofuran derivatives from the Antarctic lichen *R. terebrata* might represent new structural classes of antimicrobial, anti-proliferative, and anti-inflammatory agents.

## 3. Materials and Methods

### 3.1. General Experimental Procedures

Optical rotations were measured in the wavelength range 254–880 nm at 25 °C using Jasco P-2000 polarimeter (Jasco, MD, USA). Infrared (IR) spectra were recorded on Nicolet iN10 MX FT-IR microscope (Thermo Fisher Scientific, Waltham, MA, USA). Ultraviolet (UV) spectra were acquired on Agilent 8453 UV-visible spectrophotometer (Agilent Technologies, Santa Clara, CA, USA). Mass spectra were recorded on JEOL JMS-700 mass spectrometer (JEOL Ltd., Akishima, Tokyo, Japan). NMR spectra were measured using a Bruker AVANCE III operating at 600 MHz (^1^H) and 150 MHz (^13^C) (Bruker, Billerica, MA, USA). LC/MS analysis was performed using an Agilent 1200 series HPLC system with a diode array detector and a 6130 Series ESI mass spectrometer. Preparative high-performance liquid chromatography (HPLC) was conducted using a Waters 2545 binary HPLC pump with Waters 2998 photodiode array detector (Waters, Milford, MA, USA) and YMC-Pack ODS-A-HG column (250 × 20 mm, 10 μm; flow rate: 25 mL/min) (Waters). Semi-preparative HPLC was conducted on YL 9100 HPLC system (Young Lin, South Korea) equipped with a UV/Vis detector using an Alltech reversed-phase YMC-Pak C-18 column (10 μm, 20 × 250 mm) and a normal-phase YMC-Pack SIL-HG column (S-10 μm, 12 nm, 20 × 250 mm). Column chromatography was performed using silica gel (230–400 mesh, Merck, Darmstadt, Germany) and C-18 (YMC·GEL ODS-A, 12 nm, S-150 μm). Thin-layer chromatography (TLC) was conducted using silica gel 60 F254 (0.25 mm, Merck) plates and reverse-phase (RP)-18 F254s plates (Merck). Spots on TLC were detected using UV and heating after dipping in 20% sulfuric acid in H_2_O.

### 3.2. Lichen Material

The specimens of lichen, *R. terebrata*, were collected from King George Island, Antarctica, (62°12′53.69′′ S; 58°55′23.87′′ W) in January 2021, and identified by Dr. Ji Hee Kim. A voucher specimen (An-L16) was deposited at the Natural Product Chemistry Laboratory of the Korea Polar Research Institute.

### 3.3. Extraction and Isolation

The air-dried and powdered lichen, *R. terebrata* (14.0 g), was extracted in acetone by maceration (2 × 1 L) at room temperature. Extracts were filtered, and the filtrate was evaporated under reduced pressure with a rotavapor to obtain a crude extract (1.9 g). From the concentrated crude extract, a large portion of yellow powder (292.0 mg) was separated into a different fraction by scraping from the surface of the flask. It was then extracted with chloroform and subsequently subjected to preparative HPLC (MeOH/H_2_O, 90:10→100:0) to give compound **3** (t_R_ 48.0 min, 82.0 mg). The remaining crude extract was subjected to silica gel column chromatography (CC) (hexane/EtOAc, 50:1→1:1 and CHCl_3_/MeOH, 20:1→1:1) to yield 10 fractions (Fr. A1–A10). Fr. Fr. A6 (29.0 mg) was subjected to semi-preparative HPLC (MeCN/H_2_O, 90:10→100:0) to give compound **1** (t_R_ 46.5 min, 2.0 mg) and subfraction A62 (6.0 mg), which was repurified using the same method (MeOH/ H_2_O, 97:3) to yield compound **2** (t_R_ 35.5 min, 3.0 mg).

**Stereocalpin B (1)**: White powder; ${\left[\alpha \right]}_{D}^{25}$−128 (c 0.1, MeOH); UV (MeOH) *λ*_max_ (log *ε*) = 208 (2.6) nm; ECD (MeOH) *λ*_max_ (Δ*ε*) 235 (−552.4), 300 (−489.3) nm; IR (KBr) *ν*_max_: 3297, 2933, 1742, 1653, 1454, and 1200 cm^−1^; (+)-HR-FABMS *m/z* 521.3017 [M + H]^+^ (calculated for C_31_H_41_N_2_O_5_, 521.3015); for ^1^H and ^13^C NMR spectroscopic data, see Table 1.

**1,3,7,9-tetrahydroxy-2,8-dimethyl-4,6-di(ethanoyl)dibenzofuran (3)**: Yellow powder; UV (MeOH) *λ*_max_ (log *ε*) = 230 (3.7) nm, 285 (2.5) nm; IR (KBr) *ν*_max_: 3370, 2931, 1697, 1632, 1564, 1396, 1289, 1192, and 1067 cm^−1^; (+)-HR-FABMS *m/z* 345.0972 [M + H]^+^ (calculated for C_18_H_17_O_7_, 345.0974); for ^1^H and ^13^C NMR spectroscopic data, see Table 2.

### 3.4. Advanced Marfey’s Analyses of FDAA Derivatives Using LC-MS

A portion of stereocalpin B (**1**, 1.0 mg) was hydrolyzed in 6 N HCl (1 mL) at 100 °C overnight. The reaction mixture was evaporated to dryness and dissolved in 150 μL H_2_O. To the solution, 70 μL of 1 M NaHCO_3_ and 200 μL of 1% FDAA in acetone were added, and the reaction mixture was stirred at 37 °C for 1 hr. The reaction was quenched with the addition of 100 μL of 1 N HCl. The DAA derivatives were analyzed by LC-MS using an analytical Kinetex C18 100 Å column (100 mm × 2.1 mm i.d., 5 μm; Phenomenex, Torrance, CA, USA) at a flow rate of 0.3 mL/ min. The mobile phases were 0.1% (*v*/*v*) formic acid aqueous solution (A) and 0.1% (*v/v*) formic acid acetonitrile (B) with the following gradient: 10–60% B (0–40 min), 60–100% B (40–41 min), 100% B (41–51 min), 100–10% B (51–52 min), 10% B (52–62 min). Retention times were compared to derivatized amino acid standards. Elution of the target peaks was detected by the extraction of relevant ions. Retention times for the analytical standards were as follows: L-Phe 21.8 min, D-Phe 23.9 min, *N*-Me-L-Phe 22.1 min, and *N*-Me-D-Phe 22.4 min. The L-DLA-derivatized hydrolysate of **1** gave peaks at 21.7 min and 22.0 min, which confirmed the presence of L-Phe and *N*-Me-L-Phe (Figure S11).

### 3.5. Computational Details

Initial conformational searches were performed at the MMFF94 force field using the MacroModel (Version 9.9, Schrödinger LLC) program with a mixed torsional/low-mode sampling method where the gas phase with a 20 kJ/mol energy window and 10,000 maximum iterations were employed. Quantum-mechanics geometry optimization of all the identified conformers within 10 kJ/mol of the relative MMFF94 energy level was performed utilizing the Tmolex 4.3.1 at the B3LYP/6-31 + G (d, p) theory level with the polarizable continuum model (PCM) (methanol) for solvation.

### 3.6. PANIC Analysis

The PANIC analysis was carried out according to the previous communication with slight modification. Slices of 2D NOESY spectrum of **1** were obtained using MestReNova software (version 14.1.2-25024). As a reference, the interproton distance between two aromatic protons with *ortho*-relationship, H-5 and H-6 ($r_{\mathrm{reference}}$), was set as 2.5 Hz. The corresponding NOE intensity (${\mathrm{NOE}}_{\mathrm{reference}}$) was obtained by integrating the signal of H-6 from the slice of 2D NOESY spectrum irradiated at H-5. The NOE intensities of H-3a in **1a** (${\mathrm{NOE}}_{\mathrm{unknown}}$), and H_3_-10 in **1b** (${\mathrm{NOE}}_{\mathrm{unknown}}$), were acquired by irradiation at H_3_-10 and H-2 of **1a** and H-2 and H-12 of **1b** and integration of H-3a and H_3_-10 signals, respectively. The calibrated interproton distances ($r_{\mathrm{unknown}}$) were calculated using the equation below:

(1) NOEunknownNOEreference=(rreference)6(runknown)6

### 3.7. Antimicrobial Assay

The antimicrobial activities were tested with 5 microorganisms in total in 96-well-plate; *Escherichia coli* KCTC 1682 (Korea Collection for Type Cultures) and *Klebsiella pneumonia* ATCC 13883 (American Type Culture Collection) representing Gram-negative bacteria, *Staphylococcus aureus* KCTC 3881 and *Mycobacterium smegmatis* ATCC 607 representing Gram-positive bacteria and *Candida albicans* KCTC 27242 representing fungi. Cell culture (95 μL) was diluted to 0.5 McFarland Standard scale in each well. Compounds dissolved in DMSO were added up to final concentrations (0.5, 1, 2, 5, 10, 20, 50, and 100 μg/mL). Total culture (100 μL) was incubated at 37 °C for 16 h. Cell inhibition was measured at 600 nm using MultiskanTM GO Microplate Spectrophotometer (Thermo Scientific, Waltham, MA, USA). The IC_50_ value was calculated using an exponential trend line in Excel (Microsoft, Redmond, WA, USA). Apramycin and nystatin were used as a positive control against the bacterium and yeast, respectively. All measurements were performed in triplicates.

### 3.8. Cell Culture

RAW264.7 macrophages and HCT116 were cultured in Dulbecco’s modified Eagle’s medium (DMEM) supplemented with 10% fatal bovine serum (FBS) and 1% penicillin at 37 °C in a humidified CO_2_ incubator. In this study, macrophages were subjected in the presence or absence of different concentrations of the isolated compounds which were added 1 h prior to LPS (0.5 μg/mL) stimulation. HCT116 cells were seeded in a 96-well plate in triplicate at a density of 5 × 10^3^ cells/well, and incubated in 5% CO_2_ supplement at 37 °C.

### 3.9. MTS Assay

MTS assay was performed to determine the cytotoxic effects of the isolated compounds against HCT116 cells. The cells were seeded 2 × 10^5^ cells/mL on a 96-well plate. After incubation for 24 h, 10% MTS solution was added to the cell culture medium, and it was incubated at 37 °C for 1 h. The concentration of the treated compounds was from 50 μM to 6.25 μM, using serial dilution. The absorbance was measured after 24 h using a microplate reader (Promega., Madison, WI, USA) at 490 nm.

### 3.10. Measurement of nitric oxide (NO) production

NO concentration in the RAW264.7 cell culture supernatant was measured using the Griess reagent. Briefly, 100 μL of the collected supernatant was mixed with equal amounts of Griess reagent (1% sulfanilamide in 5% phosphoric acid, 0.1% *N*-(1-naphthyl) ethylenediamine). The mixtures were incubated for 10 min at room temperature, and then the absorbance value of each well was determined at the wavelength of 540 nm using a microplate reader. Nitrite concentration was determined using a sodium nitrite calibration curve (0–100 μM).

## Figures and Tables

**Table 1:** ^1^H and ^13^C NMR data of stereocalpin B (**1**) in CDCl_3_^a^.

Position	1a		1b	
	*δ*_H_ (m, *J* in Hz)	*δ*_C_	*δ*_H_ (m, *J* in Hz)	*δ*_C_
1		171.2		169.0
2	4.90 dd (11.0, 4.6)	59.5	3.33 dd (11.2, 3.2)	67.3
3	3.04 ^b^, 3.20 ^b^	35.3	3.04 ^b^, 3.27 dd (13.8, 3.2)	34.9
4		138.1		135.8
5	6.60 d (7.2)	128.9	7.39 d (7.0)	128.9
6	7.05 t (7.4)	128.3	7.31 ^b^	129.0
7	7.11 t (7.4)	126.2	7.24 ^b^	127.2
8	7.05 t (7.4)	128.3	7.31 ^b^	129.0
9	6.60 d (7.2)	128.9	7.39 d (7.0)	128.9
10	3.06 s	29.5	2.56 s	39.4
11		170.4		171.7
12	5.08 ddd (9.5, 8.0, 6.6)	50.5	5.18 ddd (11.0, 9.2, 4.9)	49.8
13	2.89 dd (14.1, 8.0), 3.12 dd (14.1, 6.6)	38.0	3.06 ^b^, 3.18 ^b^	36.8
14		136.5		135.7
15	7.08 d (7.0)	129.3	7.28 d (7.1)	128.9
16	7.24 t (7.0)	128.3	7.23 ^b^	128.4
17	7.18 t (7.0)	126.5	7.39 ^b^	127.2
18	7.24 t (7.0)	128.3	7.23 ^b^	128.4
19	7.08 d (7.0)	129.3	7.28 d (7.1)	128.9
NH	6.17 d (9.5)		6.65 d (9.2)	
20		166.7		168.3
21	3.17 m ^b^	57.0	3.43 q (6.5)	55.8
22	0.93 d (6.6)	13.6	1.27 d (6.5)	12.2
23		205.2		205.4
24	3.22 m ^b^	47.3	3.00 m ^b^	48.5
25	1.04 d (7.1)	14.3	1.14 d (7.4)	15.9
26	4.71 dt (11.8, 3.6)	77.3	5.04 dt (10.4, 2.7)	77.2
27	1.73 ^b^	29.7	1.83 ^b^	29.7
28	1.66 ^b,c^	27.3	1.69 ^b,c^	27.3
29	1.22 ^b^	31.3	1.22 ^b^	31.3
30	1.31 ^b^	22.2	1.31 ^b^	22.2
31	0.85 t (6.7) ^b,c^	13.9	0.86 t (6.7) ^b,c^	13.9

^a^ 600 MHz for ^1^H and 150 MHz for ^13^C; coupling constants (in Hz) are in parentheses. Assignments were based on heteronuclear single quantum coherence (HSQC), heteronuclear multiple bond correlation (HMBC), and ^1^H-^1^H COSY spectra. ^b^ Overlapped. ^c^ Exchangeable.

**Table 2:** ^1^H and ^13^C NMR data of compound **3** in DMSO-*d*_6_^a^.

Position	δH (m, J in Hz)	δC	Position	δH (m, J in Hz)	δC
1a		105.0	9		157.0
1		157.0	9a		105.0
2		106.9	2-CH_3_	1.99 s	7.5
3		162.4	8-CH_3_	1.99 s	7.5
4		101.0	1′		200.8
4a		155.4	2′	2.66 s	31.0
5			1′′		200.8
6a		155.4	2′′	2.66 s	31.0
6		101.0	3-OH	13.37 s	
7		162.4	7-OH	13.37 s	
8		106.9			

^a^ 600 MHz for ^1^H and 150 MHz for ^13^C; coupling constants (in Hz) are in parentheses. Assignments were based on HSQC and HMBC spectra.

## Data Availability

Data sharing is not applicable to this article. The data are not publicly available due to privacy.

## Supplementary Materials

The following supporting information can be downloaded at: https://www.mdpi.com/article/10.3390/metabo12020141/s1, Figure S1: HR-FABMS data of **1**, Figure S2: UV spectrum of **1**, Figure S3: IR spectrum of **1**, Figure S4: ^1^H NMR spectrum of **1**, Figure S5: ^1^H-^1^H COSY spectrum of **1**, Figure S6: NOESY spectrum of **1**, Figure S7: HSQC spectrum of **1**, Figure S8: HMBC spectrum of **1**, Figure S9: MS/MS spectrum analysis of **1**, Figure S10: Illustrative portion of the NOESY/EXSY spectrum of **1** showing exchanged cross-peaks (light blue) between signals of **1a** and **1b**, Figure S11: Advanced Marfey’s analysis of **1**, Figure S12: Possible stereoconversion of the C-22 methyl group of the proposed structure of stereocalpin A (**2**), Figure S13: Slice of 2D NOESY spectrum with H-5 irradiated for PANIC, Figure S14: Slice of 2D NOESY spectrum with H_3_-10 in **1a** irradiated for PANIC, Figure S15: Slice of 2D NOESY spectrum with H-2 in **1a** irradiated for PANIC, Figure S16: Slice of 2D NOESY spectrum with H-2 in **1b** irradiated for PANIC, Figure S17: Slice of 2D NOESY spectrum with H-12 in **1b** irradiated for PANIC, Figure S18: HR-FABMS data of **3**, Figure S19: UV spectrum of **3**, Figure S20: IR spectrum of **3**, Figure S21: ^1^H NMR spectrum of **3**, Figure S22: ^13^C NMR spectrum of **3**, Figure S23: NOESY spectrum of **3**, Figure S24: HSQC spectrum of **3**, Figure S25: HMBC spectrum of **3**, Table S1: IC_50_ values of compounds **1**-**3** and **6** against *E. coli*, Table S2: Cytotoxicity of compounds **1** and **2** against HCT116 cells, Table S3: NO inhibition of compounds **1** and **2** against LPS-induced RAW264.7 cells, Table S4: Gibbs free energies and Boltzmann distribution of conformer **1a**, Table S5: Gibbs free energies and Boltzmann distribution of conformer **1b**.
