# Supplementary material for: Stereocalpin B, a New Cyclic Depsipeptide from the Antarctic Lichen Ramalina terebrata
Source: Metabolites. 2022 Feb 3;12(2):141. doi: 10.3390/metabo12020141 (PMC8880677; doi:10.3390/metabo12020141)
Supplement: Supplementary file 1 [file metabolites-12-00141-s001.zip › metabolites-1539714-SI.pdf]

Supporting Information

Stereocalpin B, a new cyclic depsipeptide from the Antarctic lichen *Ramalina terebrata*

Seulah Lee [1], Se Yun Jeong [2], Dieu Linh Nguyen [1,3], Jae Eun So [1,3], Ki Hyun Kim [2], Ji Hee Kim [1], Se Jong Han [1,3], Sung-Suk Suh [4], Jun Hyuck Lee [3,5] and Ui Joung Youn [1,3,*]

[1] Division of Life Sciences, Korea Polar Research Institute, KIOST, Incheon, 21990, Republic of Korea

[2] School of Pharmacy, Sungkyunkwan University, Suwon 16419, Republic of Korea

[3] Department of Polar Sciences, University of Science and Technology, Incheon 21990, Republic of Korea

[4] Department of Bioscience, Mokpo National University, Muan, Republic of Korea

[5] Research Unit of Cryogenic Novel Material, Korea Polar Research Institute, Incheon 21990, Republic of Korea

* Corresponding author: Ui Joung Youn, Tel: +82-32-760-5562; Fax: +82-32-760-5509; E-mail: ujyoun@kopri.re.kr

List of Electronic Supplementary Information

Figure S1. HR-FABMS data of **1**....4
Figure S2. UV spectrum of **1**....5
Figure S3. IR spectrum of **1**....6
Figure S4. ^{1}H NMR spectrum of **1** ($CDCl_3$, 600 MHz)....7
Figure S5. ^{1}H-^{1}H COSY spectrum of **1** ($CDCl_3$)....8
Figure S6. NOESY spectrum of **1** ($CDCl_3$)....9
Figure S7. HSQC spectrum of **1** ($CDCl_3$)....10
Figure S8. HMBC spectrum of **1** ($CDCl_3$)....11
Figure S9. MS/MS spectrum analysis of **1**....12
Figure S10. Illustrative portion of the NOESY/EXSY spectrum of **1** showing exchanged cross-peaks (light blue) between signals of **1a** and **1b** (red arrows)....13
Figure S11. Advanced Marfey's analysis of **1**....14
Figure S12. Possible stereoconversion of the C-22 methyl group of the proposed structure of stereocalpin A (**2**)....15
Figure S13. Slice of 2D NOESY spectrum with H-5 irradiated for PANIC....16
Figure S14. Slice of 2D NOESY spectrum with H_3-10 in **1a** irradiated for PANIC....17
Figure S15. Slice of 2D NOESY spectrum with H-2 in **1a** irradiated for PANIC....18
Figure S16. Slice of 2D NOESY spectrum with H-2 in **1b** irradiated for PANIC....19
Figure S17. Slice of 2D NOESY spectrum with H-12 in **1b** irradiated for PANIC....20
Figure S18. HR-FABMS data of **3**....21
Figure S19. UV spectrum of **3**....22
Figure S20. IR spectrum of **3**....23

Figure S21. ^{1}H NMR spectrum of **3** (DMSO-d_6, 600 MHz)....24
Figure S22. ^{13}C NMR spectrum of **3** (DMSO-d_6, 150 MHz)....25
Figure S23. NOESY spectrum of **3** (DMSO-d_6)....26
Figure S24. HSQC spectrum of **3** (DMSO-d_6)....27
Figure S25. HMBC spectrum of **3** (DMSO-d_6)....28
Table S1. IC_{50} values of compounds **1-3** against *E. coli*....29
Table S2. Cytotoxicity of compounds **1** and **2** against HCT116 cells....30
Table S3. NO inhibition of compounds **1** and **2** against LPS-induced RAW264.7 cells....31
Table S4. Gibbs free energies and Boltzmann distribution of conformer **1a**....32
Table S5. Gibbs free energies and Boltzmann distribution of conformer **1b**....32
Coordinates of the conformers....33

Figure S1. HR-FABMS data of **1**

[Mass Spectrum]
Data : FAB-B303 Date : 05-Nov-2020 15:17
RT : 0.00 min Scan# : (1,27)
Elements : C 100/0, H 100/0, N 10/0, O 10/0
Mass Tolerance : 20ppm, 5mmu if m/z < 250, 20mmu if m/z > 1000
Unsaturation (U.S.) : -0.5 - 20.0

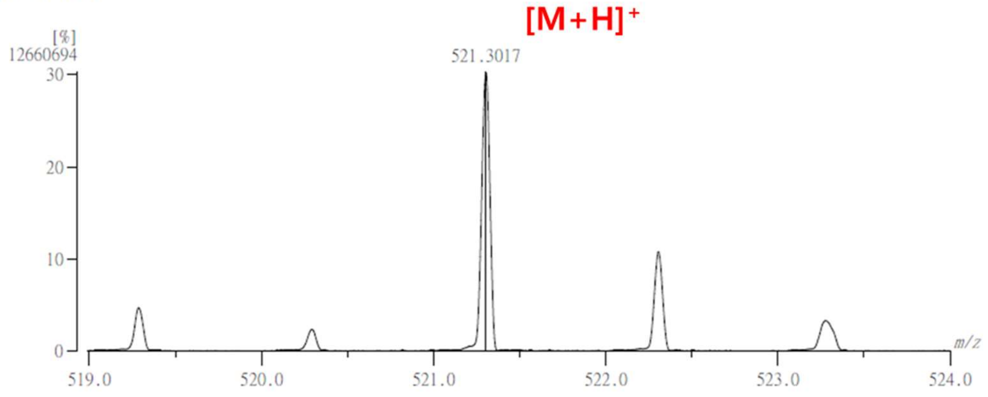

	Observed m/z	Int%	Err[ppm / mmu]	U.S.	Composition
1	521.3017	30.27	+0.3 / +0.2	18.0	C30 H35 N9
2			-2.3 / -1.2	17.5	C32 H37 N6 O
3			-4.8 / -2.5	17.0	C34 H39 N3 O2
4			+19.3 / +10.0	17.5	C33 H37 N4 O2
5			-16.1 / -8.4	13.5	C26 H37 N10 O2
6			-7.4 / -3.9	16.5	C36 H41 O3
7			+16.7 / +8.7	17.0	C35 H39 N O3
8			-18.7 / -9.7	13.0	C28 H39 N7 O3
9			+5.4 / +2.8	13.5	C27 H37 N8 O3
10			+2.9 / +1.5	13.0	C29 H39 N5 O4
11			+0.3 / +0.2	12.5	C31 H41 N2 O5
12			-11.0 / -5.7	9.0	C23 H39 N9 O5
13			+13.2 / +6.9	9.5	C22 H37 N10 O5
14			-13.5 / -7.1	8.5	C25 H41 N6 O6
15			+10.6 / +5.5	9.0	C24 H39 N7 O6
16			-16.1 / -8.4	8.0	C27 H43 N3 O7
17			+8.0 / +4.2	8.5	C26 H41 N4 O7
18			-18.7 / -9.7	7.5	C29 H45 O8
19			+5.4 / +2.8	8.0	C28 H43 N O8
20			-5.8 / -3.0	4.5	C20 H41 N8 O8
21			+18.3 / +9.5	5.0	C19 H39 N9 O8
22			-8.4 / -4.4	4.0	C22 H43 N5 O9
23			+15.7 / +8.2	4.5	C21 H41 N6 O9
24			-11.0 / -5.7	3.5	C24 H45 N2 O10
25			+13.1 / +6.9	4.0	C23 H43 N3 O10
26			+1.9 / +1.0	0.5	C15 H41 N10 O10

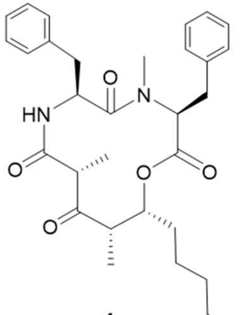

1

Figure S2. UV spectrum of **1**

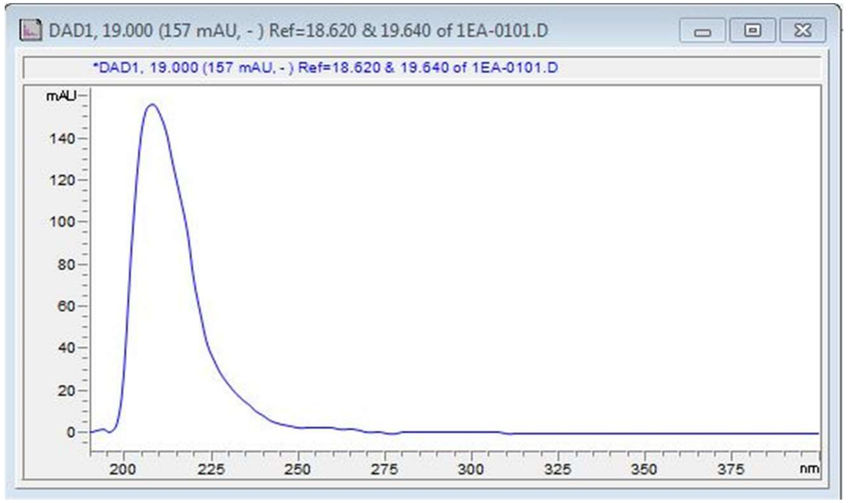

Figure S3. IR spectrum of **1**

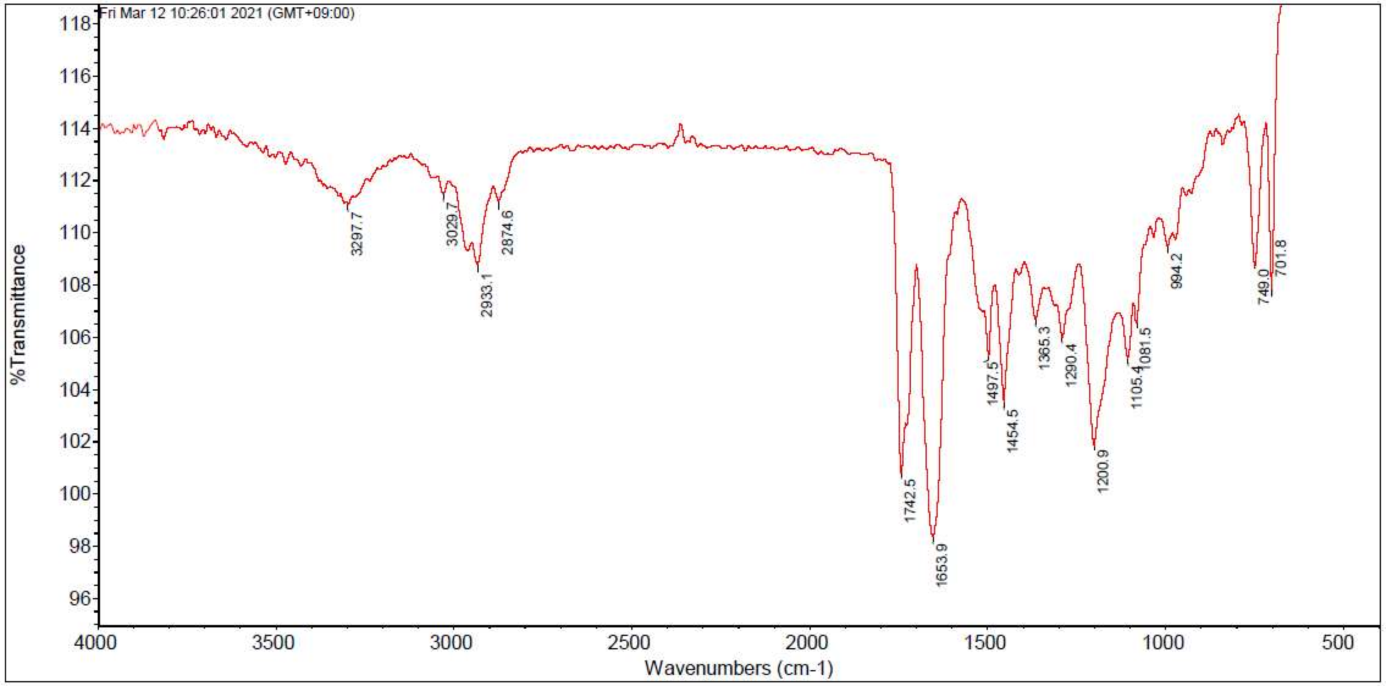

Fri Mar 12 10:32:45 2021 (GMT+09:00)
FIND PEAKS:

Spectrum:	Fri Mar 12 10:26:01 2021 (GMT+09:00)		
Region:	4000.0	400.0	
Absolute threshold:	112.472		
Sensitivity:	59		
Peak list:			

Position:	701.8	Intensity:	107.770
Position:	749.0	Intensity:	108.670
Position:	994.2	Intensity:	109.472
Position:	1081.5	Intensity:	106.450
Position:	1105.4	Intensity:	105.182
Position:	1200.9	Intensity:	101.790
Position:	1290.4	Intensity:	105.938
Position:	1365.3	Intensity:	106.697

Figure S4. ^{1}H NMR spectrum of **1** ($CDCl_3$, 600 MHz)

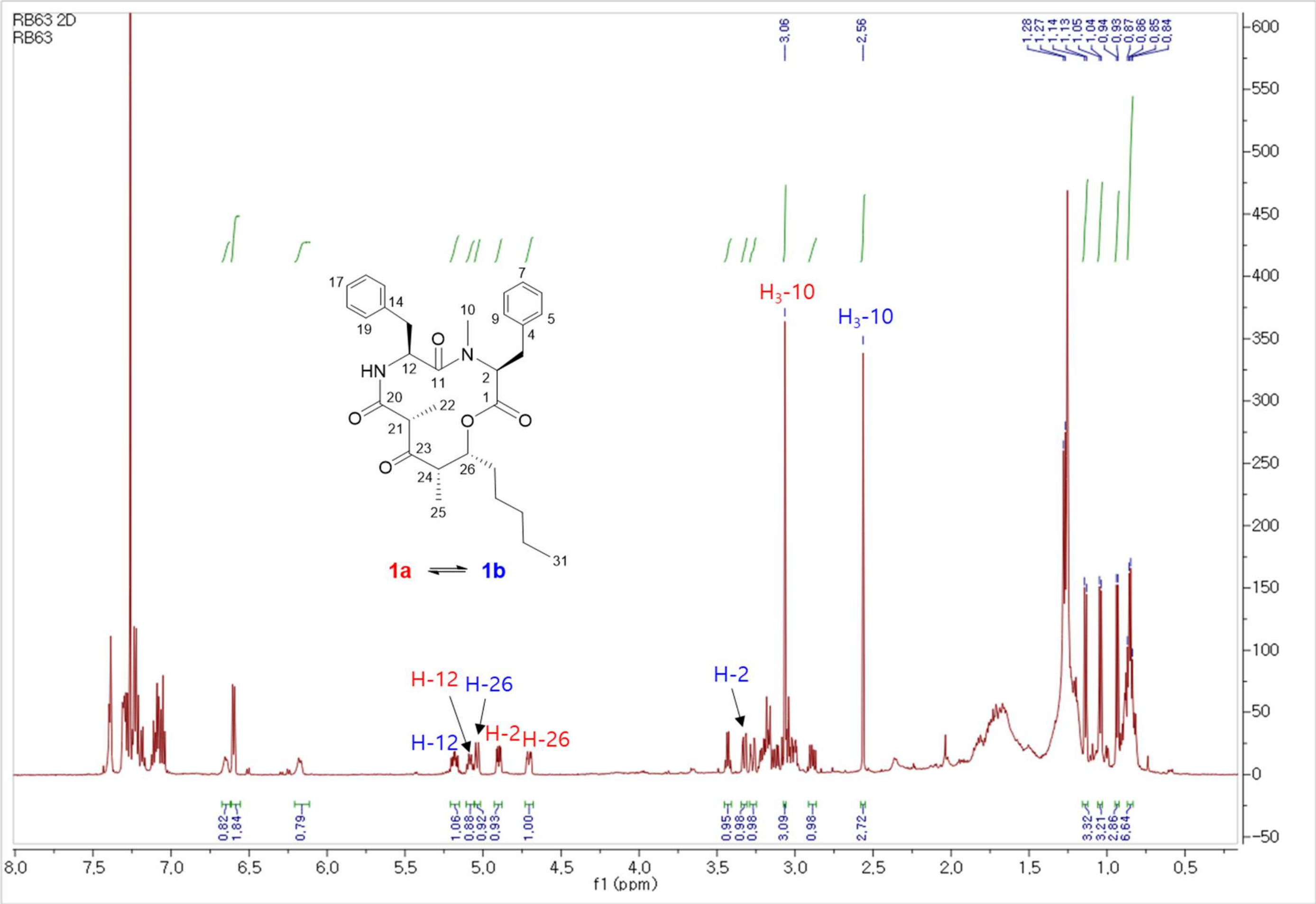

Figure S5. ^{1}H-^{1}H COSY spectrum of **1** ($CDCl_3$)

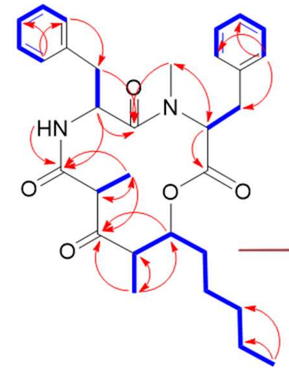

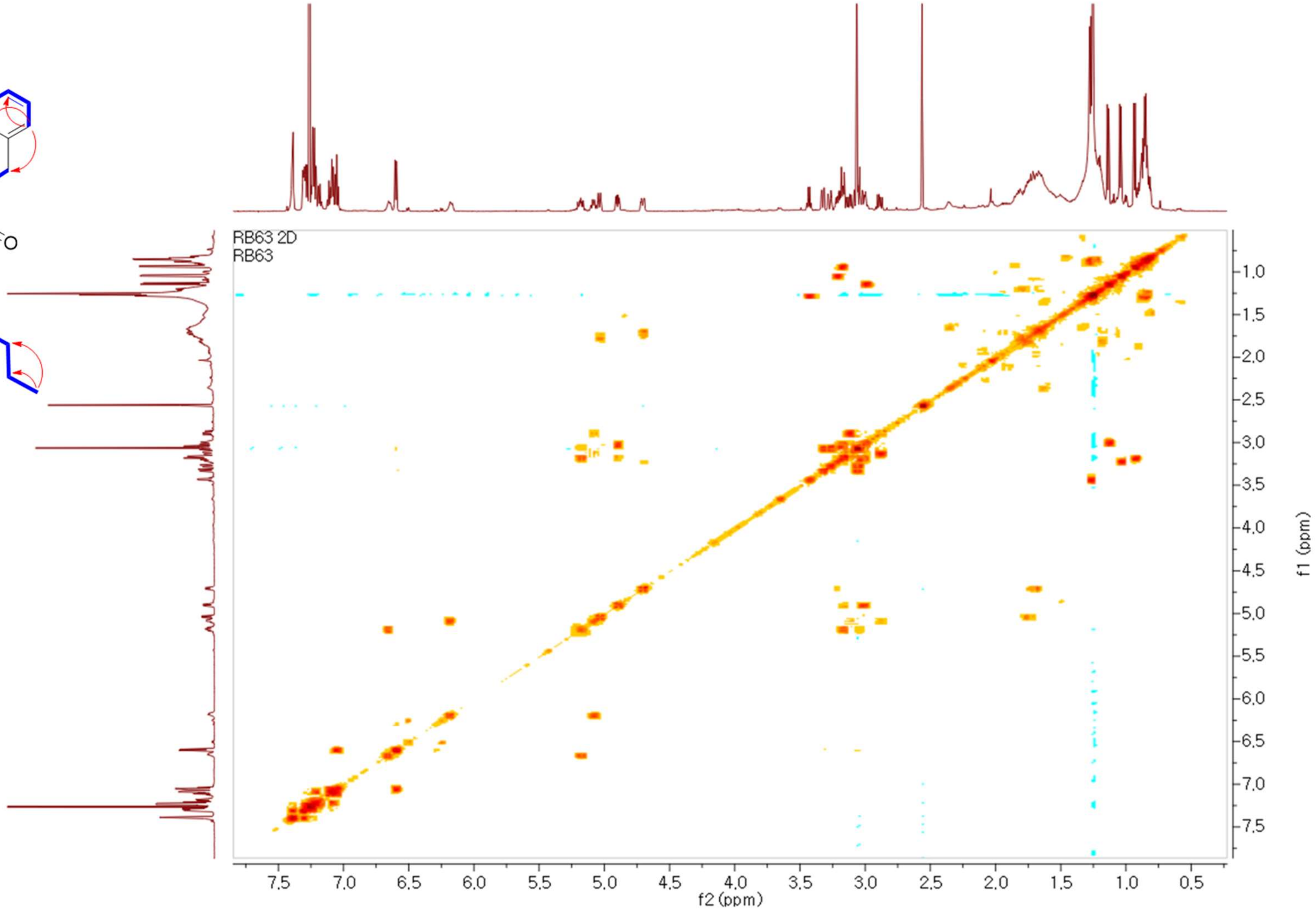

Figure S6. NOESY spectrum of **1** ($CDCl_3$)

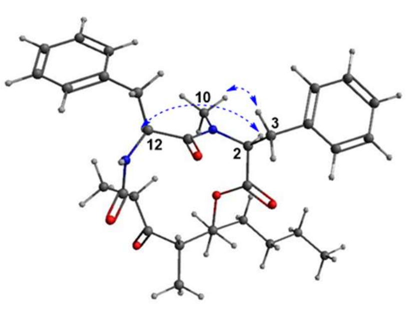

1a

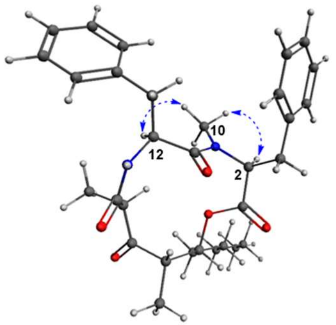

1b

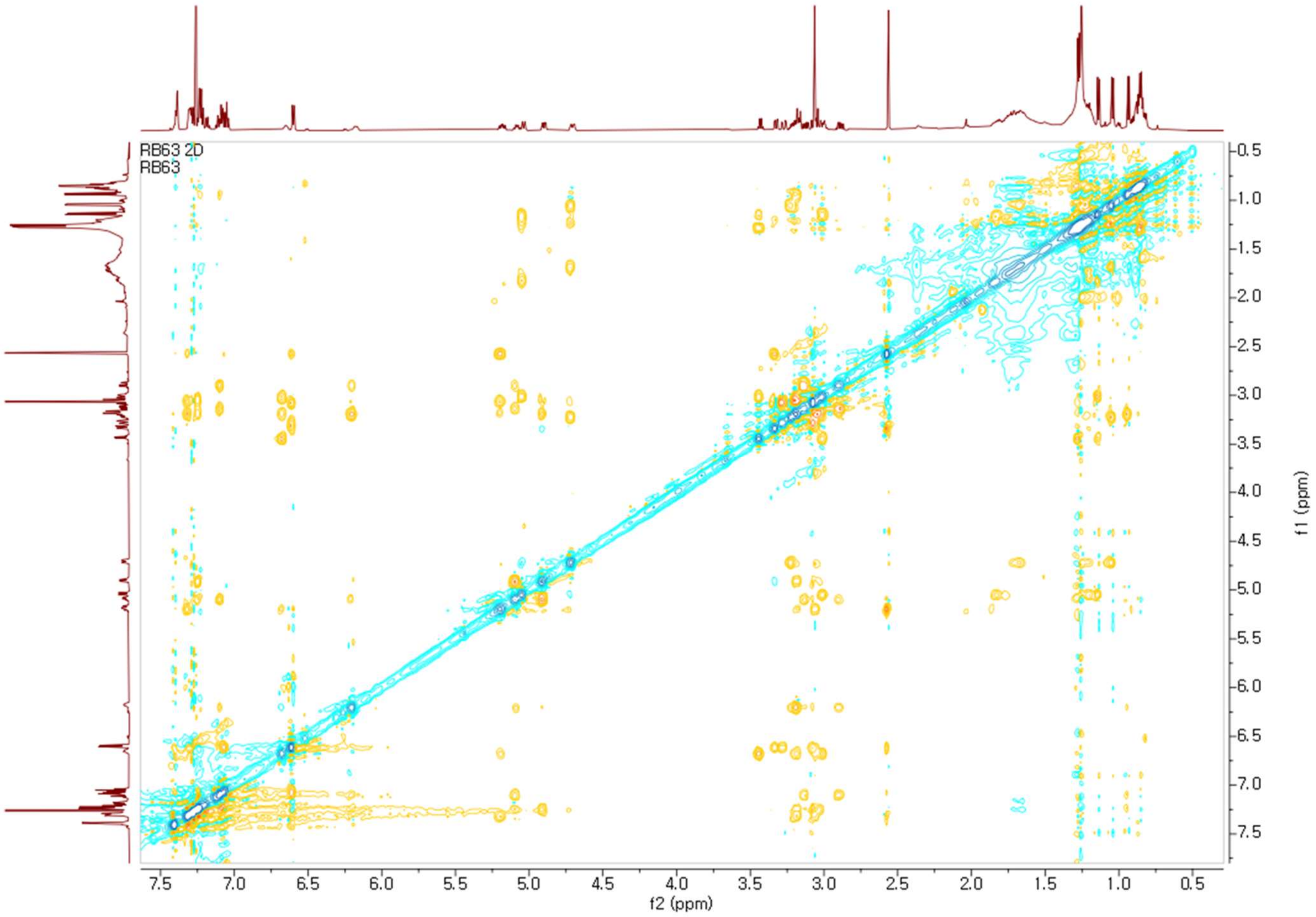

Figure S7. HSQC spectrum of **1** ($CDCl_3$)

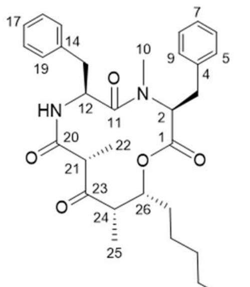

1

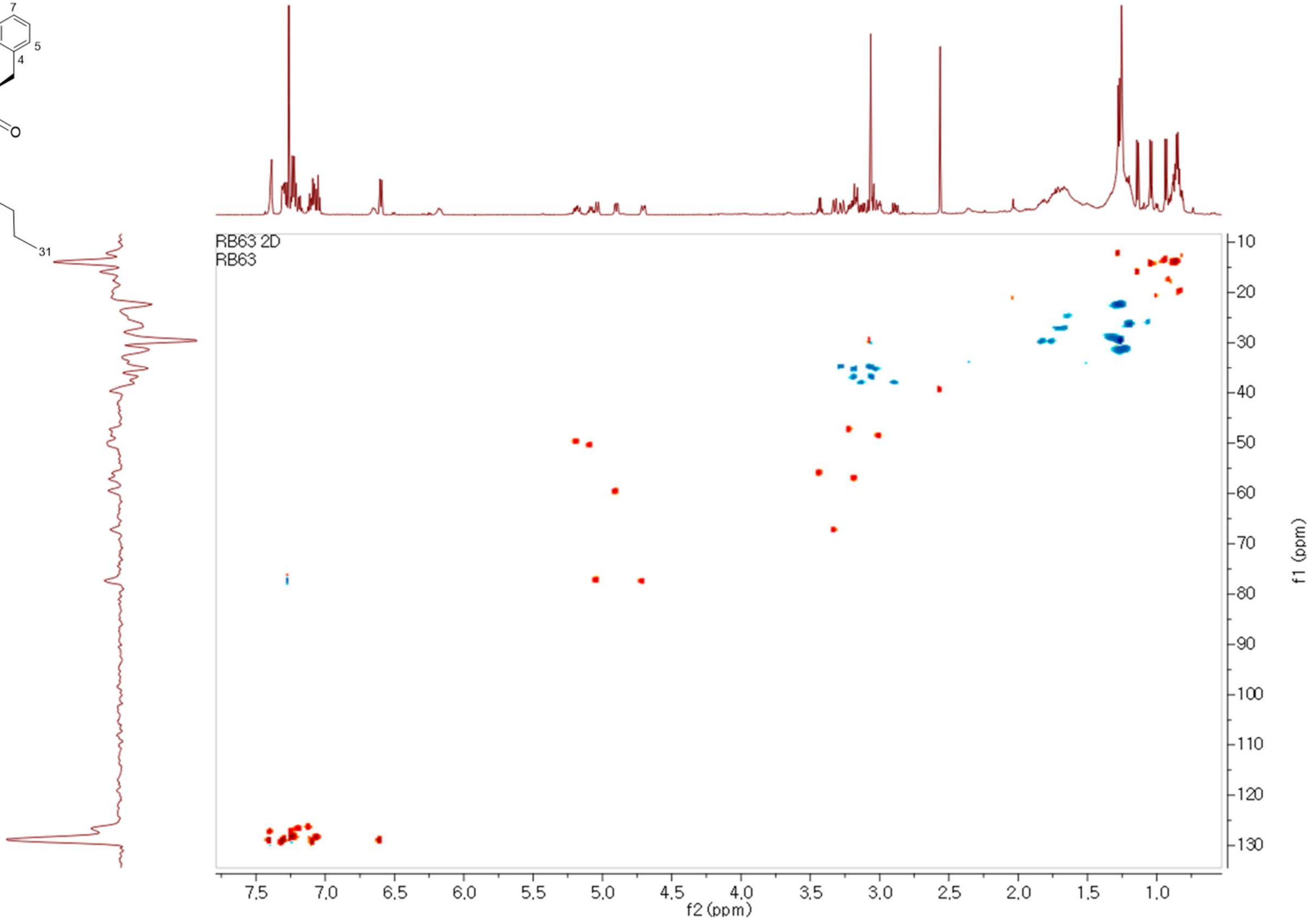

Figure S8. HMBC spectrum of **1** ($CDCl_3$)

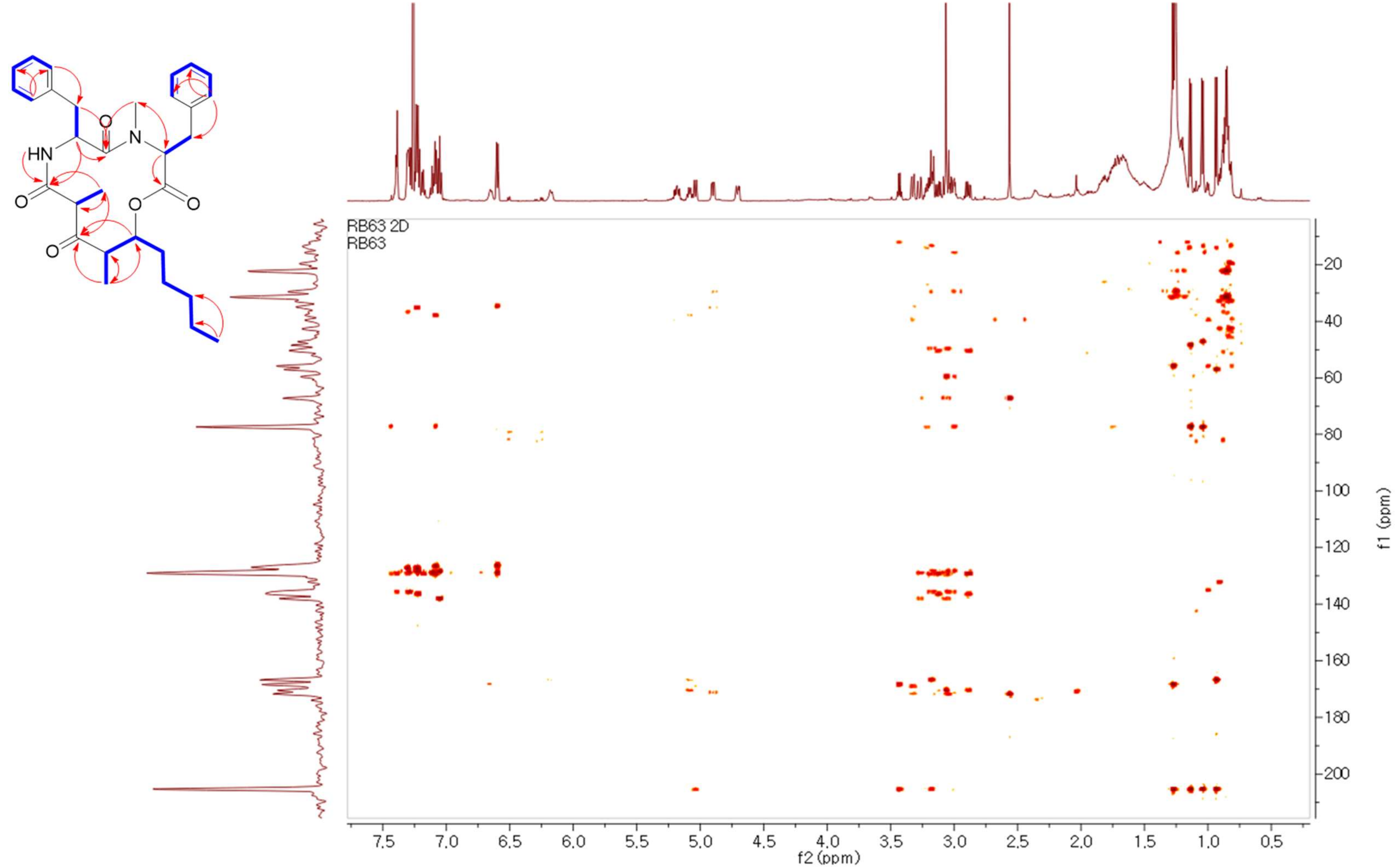

Figure S9. MS/MS spectrum analysis of **1**

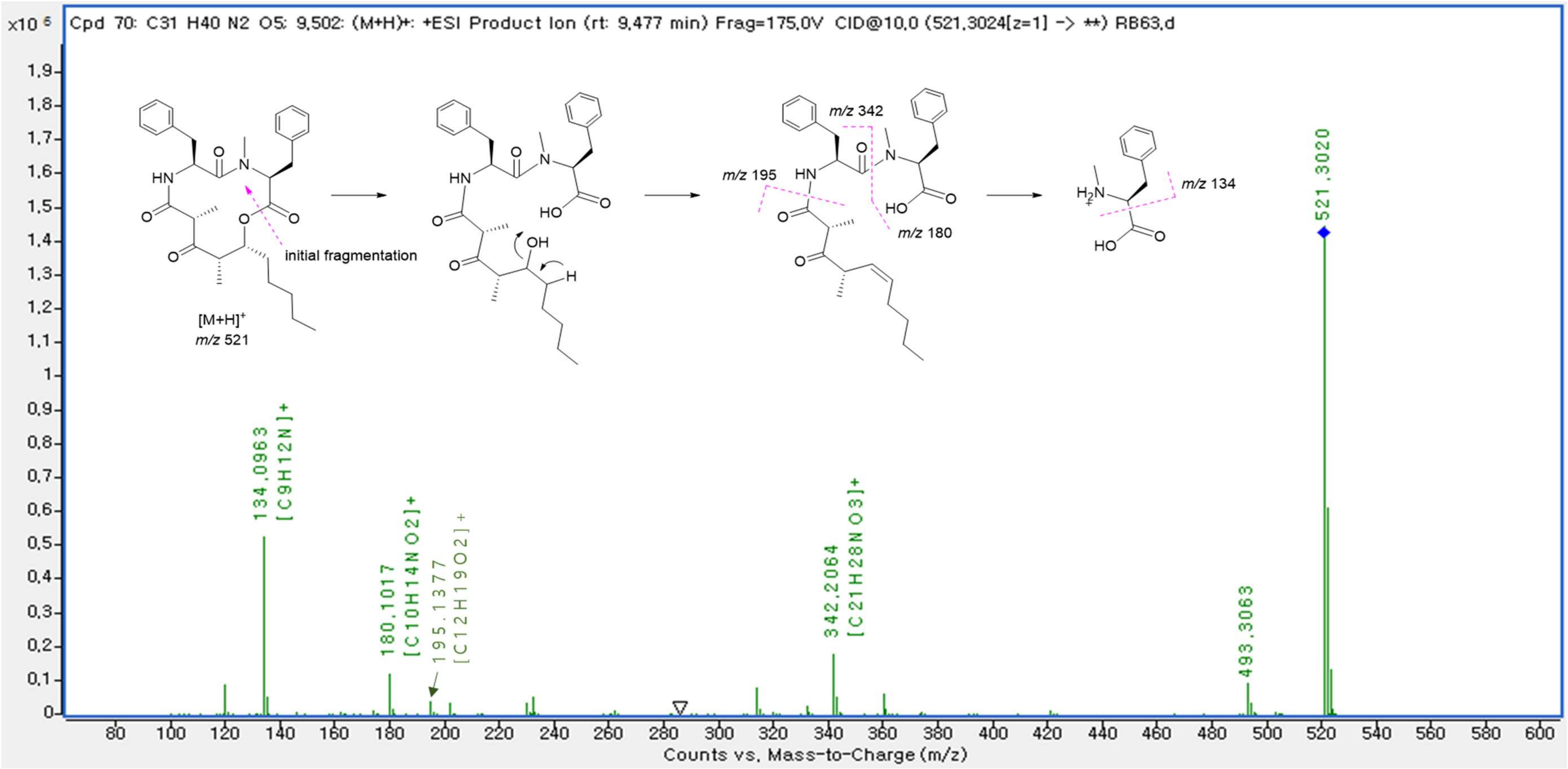

Figure S10. Illustrative portion of the NOESY/EXSY spectrum of **1** showing exchanged cross-peaks (light blue) between signals of **1a** and **1b** (red arrows)

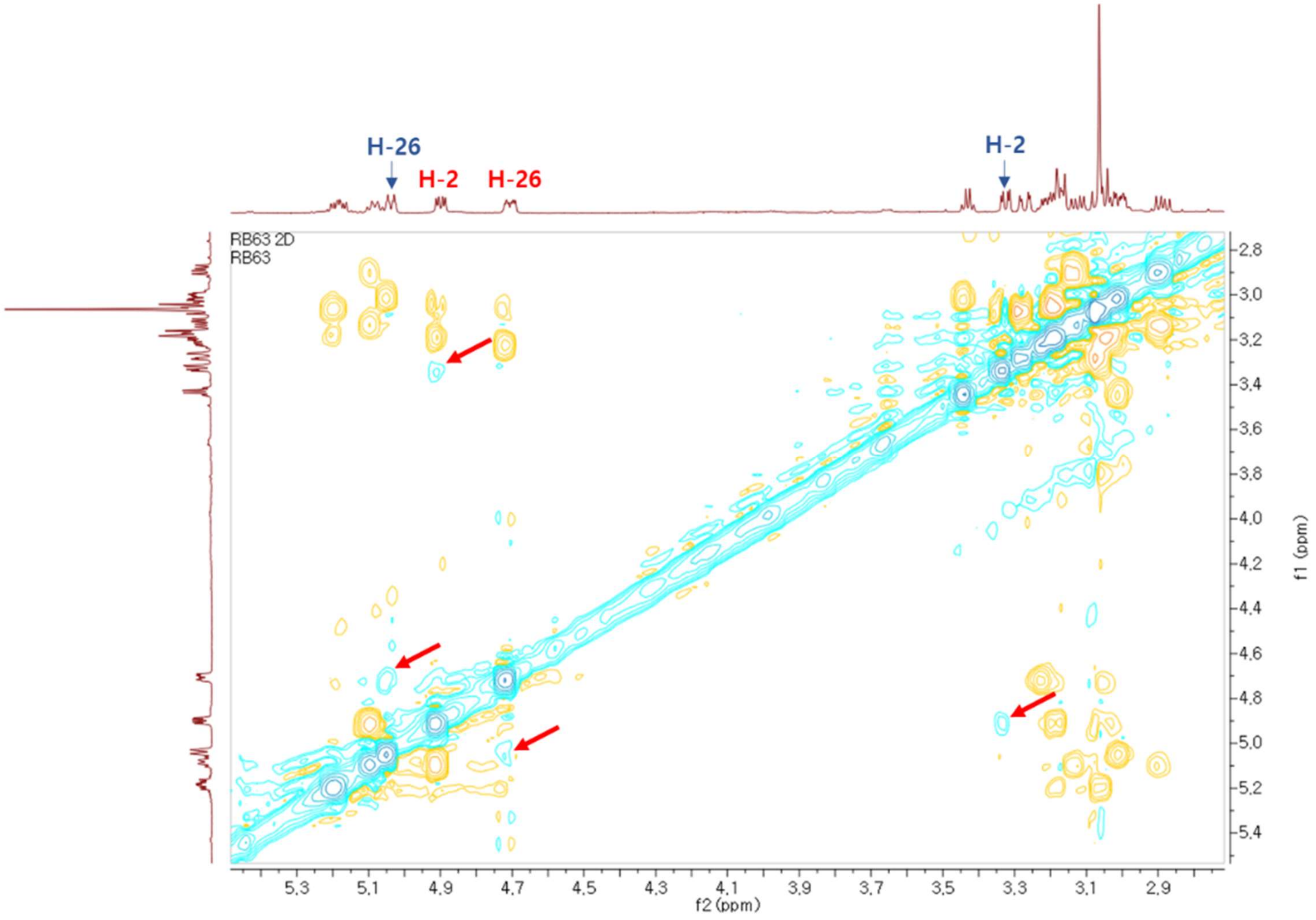

Figure S11. Advanced Marfey's analysis of **1** by LC-MS. Selective ion monitoring channels were set up at *m/z* 418 (a) and *m/z* 432 (b)

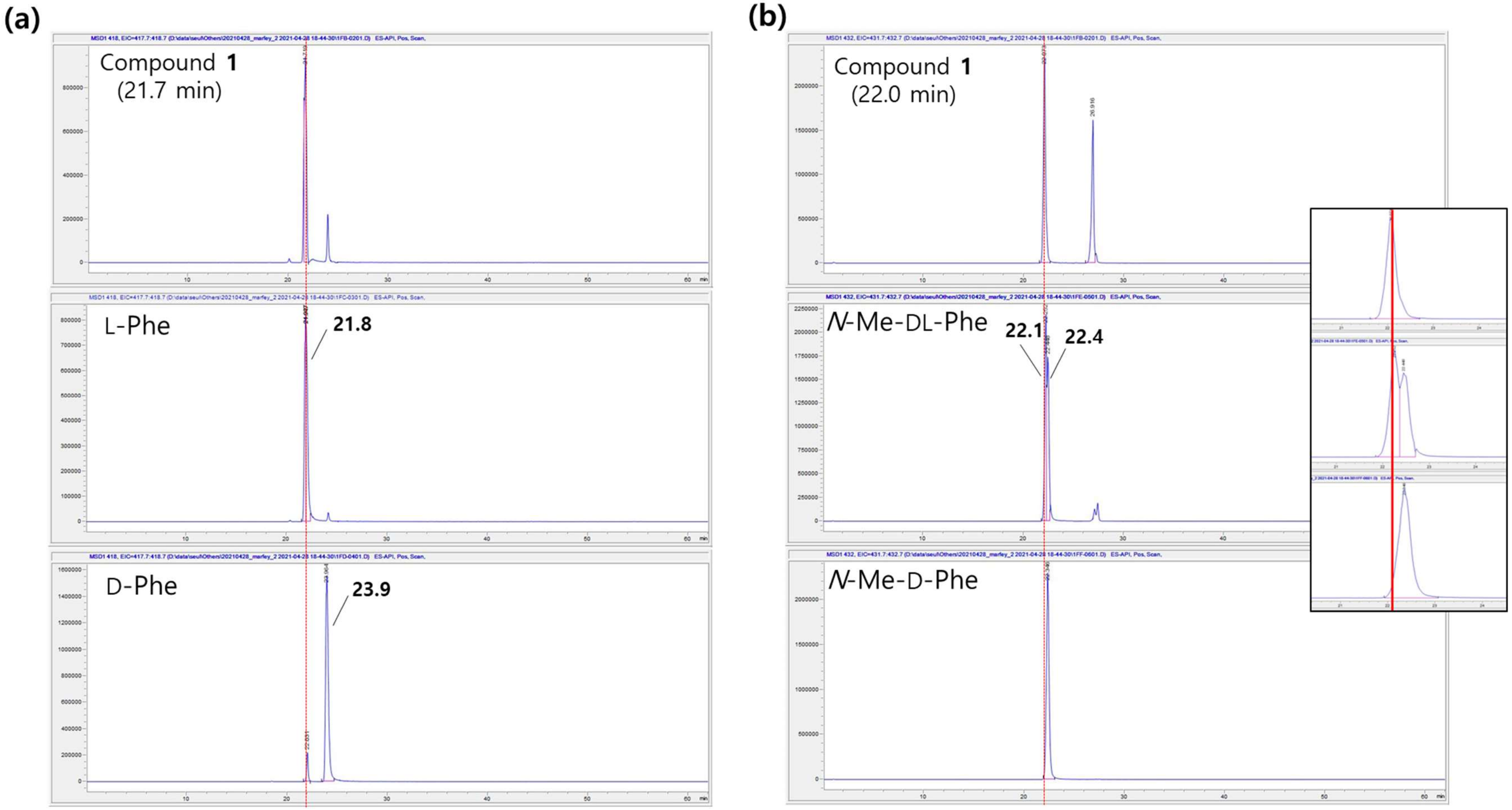

Figure S12. Possible stereoconversion of the C-22 methyl group of the proposed structure of stereocalpin A (**2**)

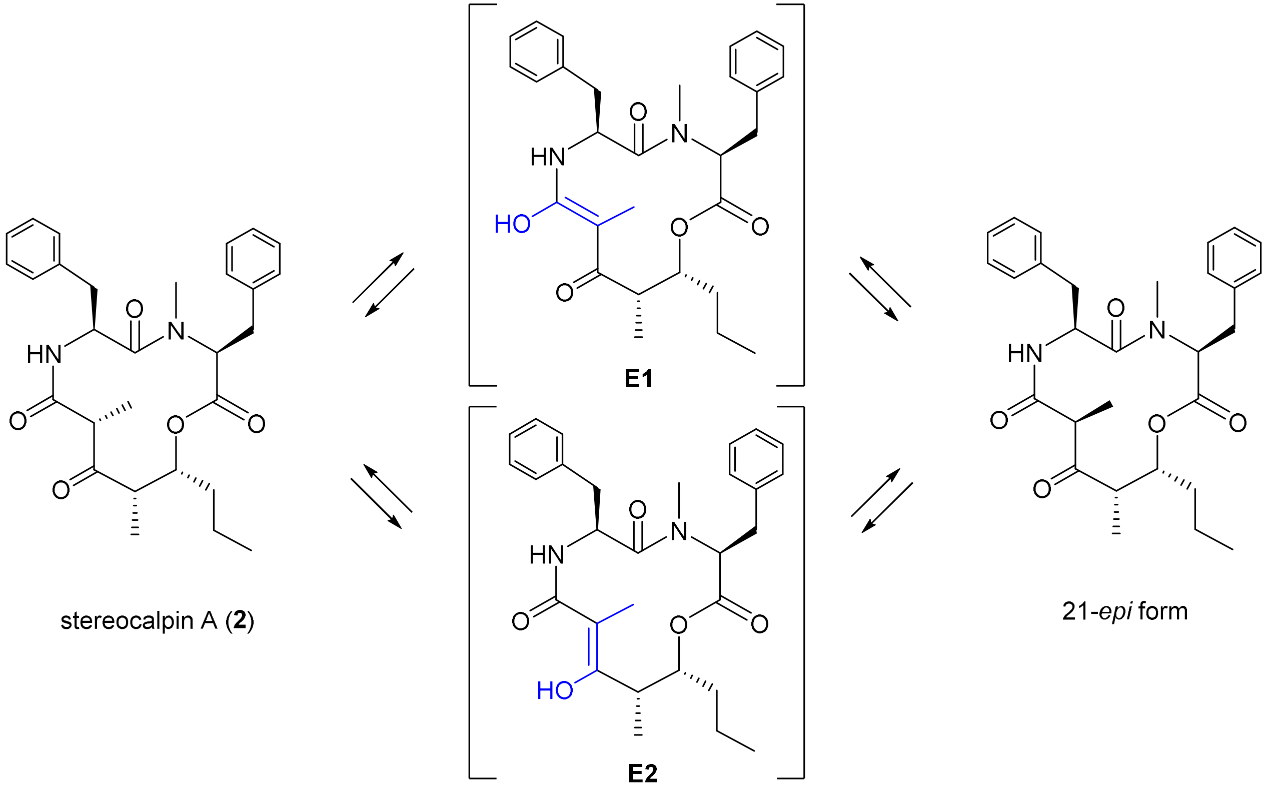

Figure S13. Slice of 2D NOESY spectrum with H-5 irradiated for PANIC

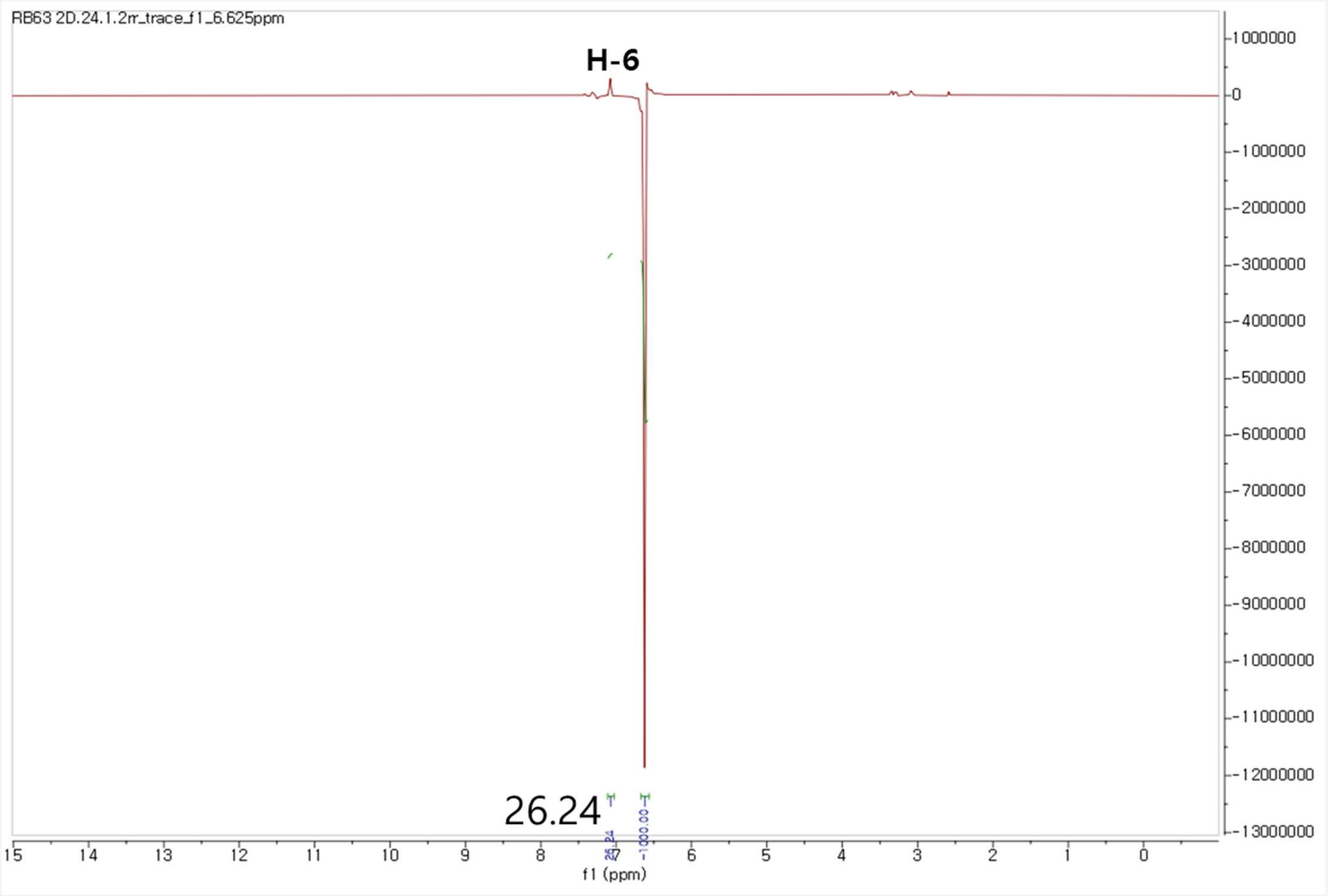

Figure S14. Slice of 2D NOESY spectrum with H_3-10 in **1a** irradiated for PANIC

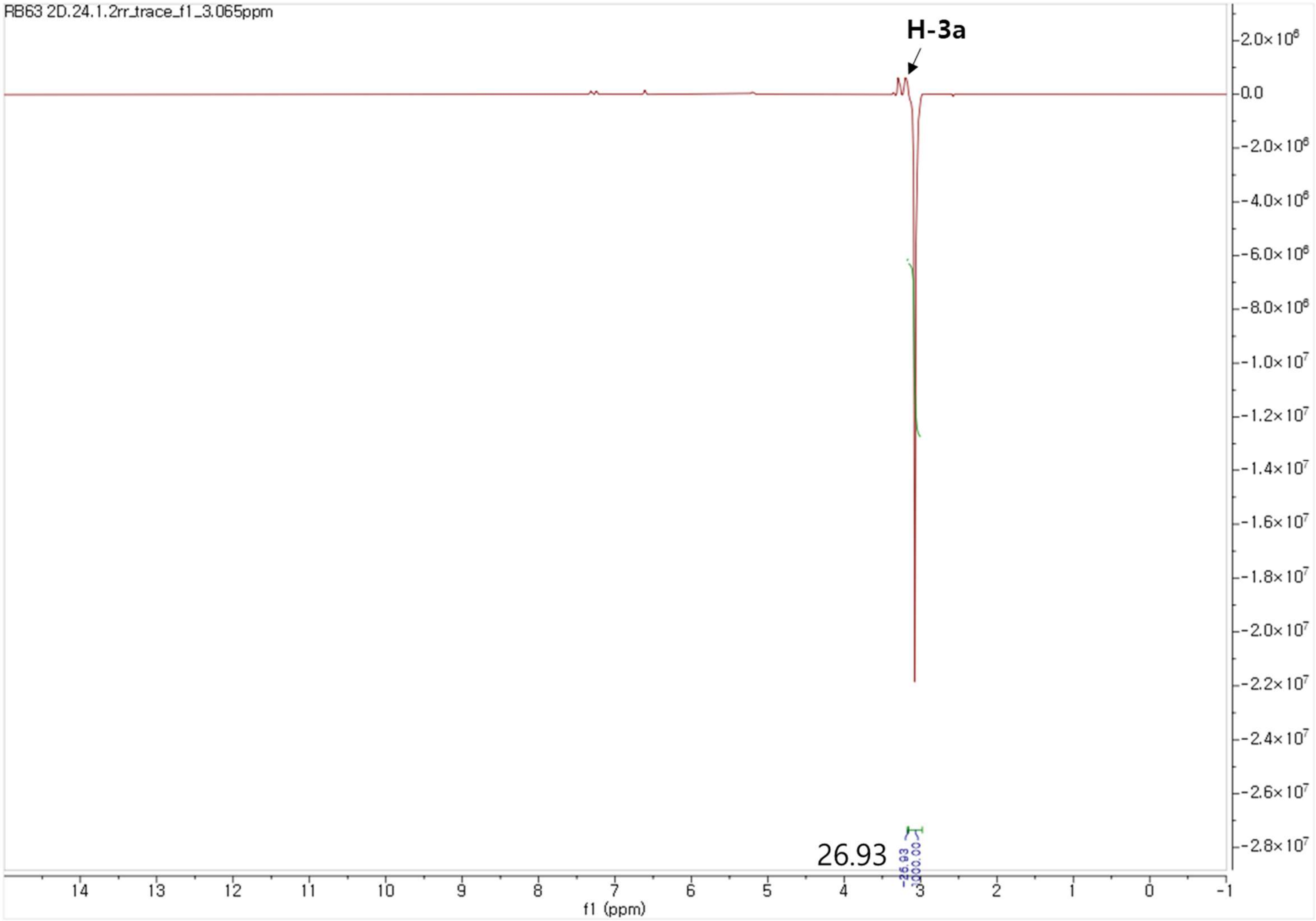

Figure S15. Slice of 2D NOESY spectrum with H-2 in **1a** irradiated for PANIC

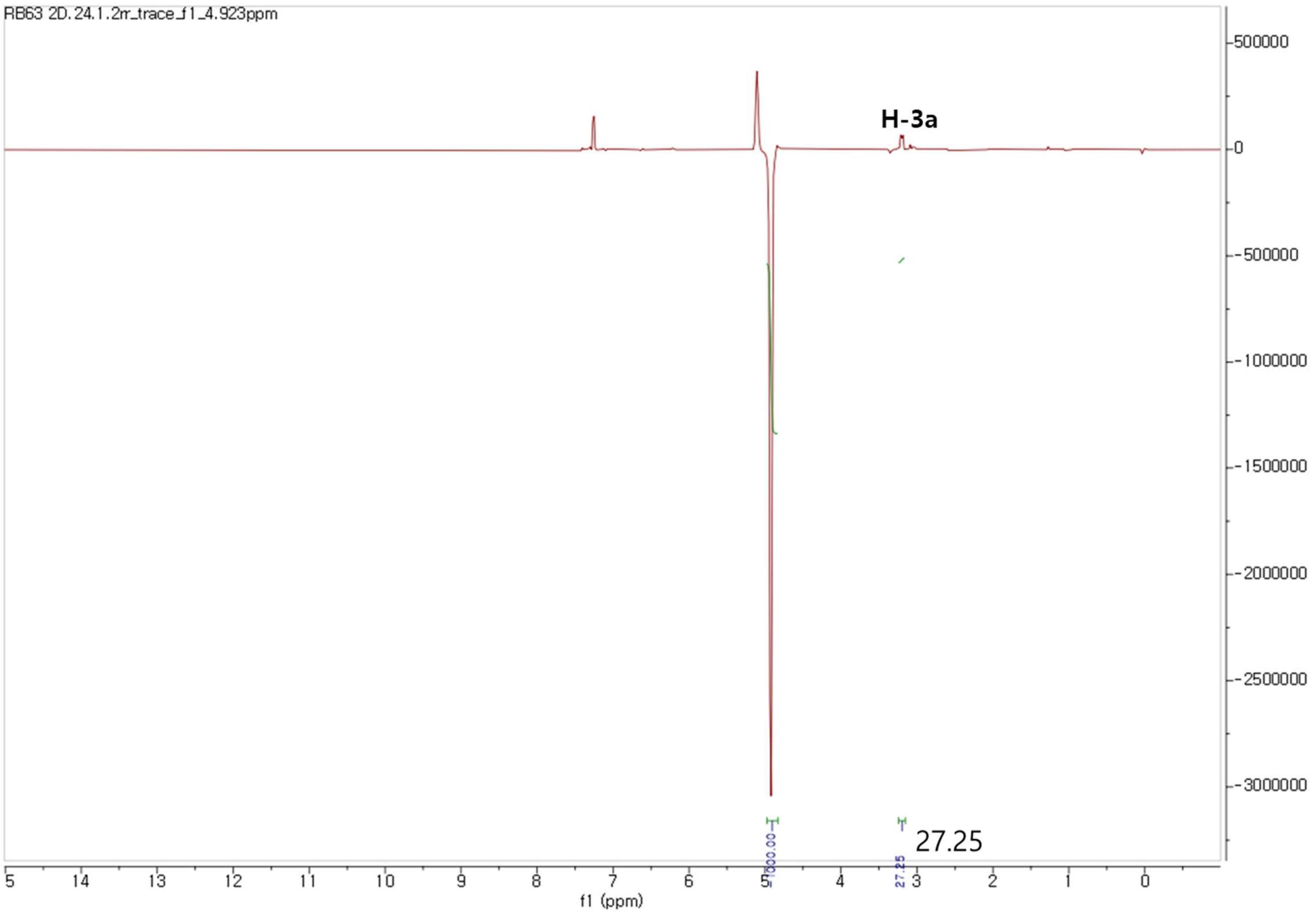

Figure S16. Slice of 2D NOESY spectrum with H-2 in **1b** irradiated for PANIC

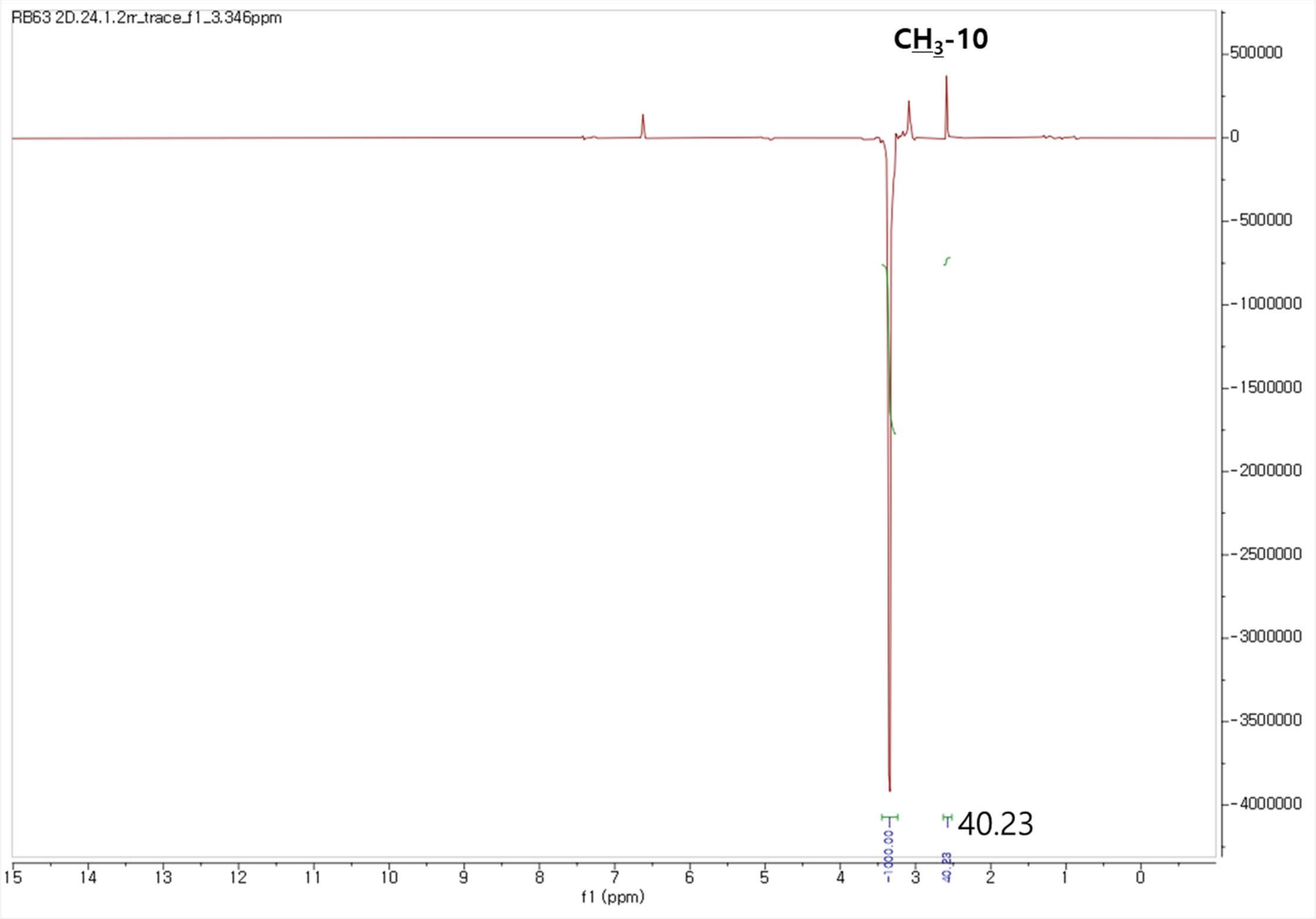

Figure S17. Slice of 2D NOESY spectrum with H-12 in **1b** irradiated for PANIC

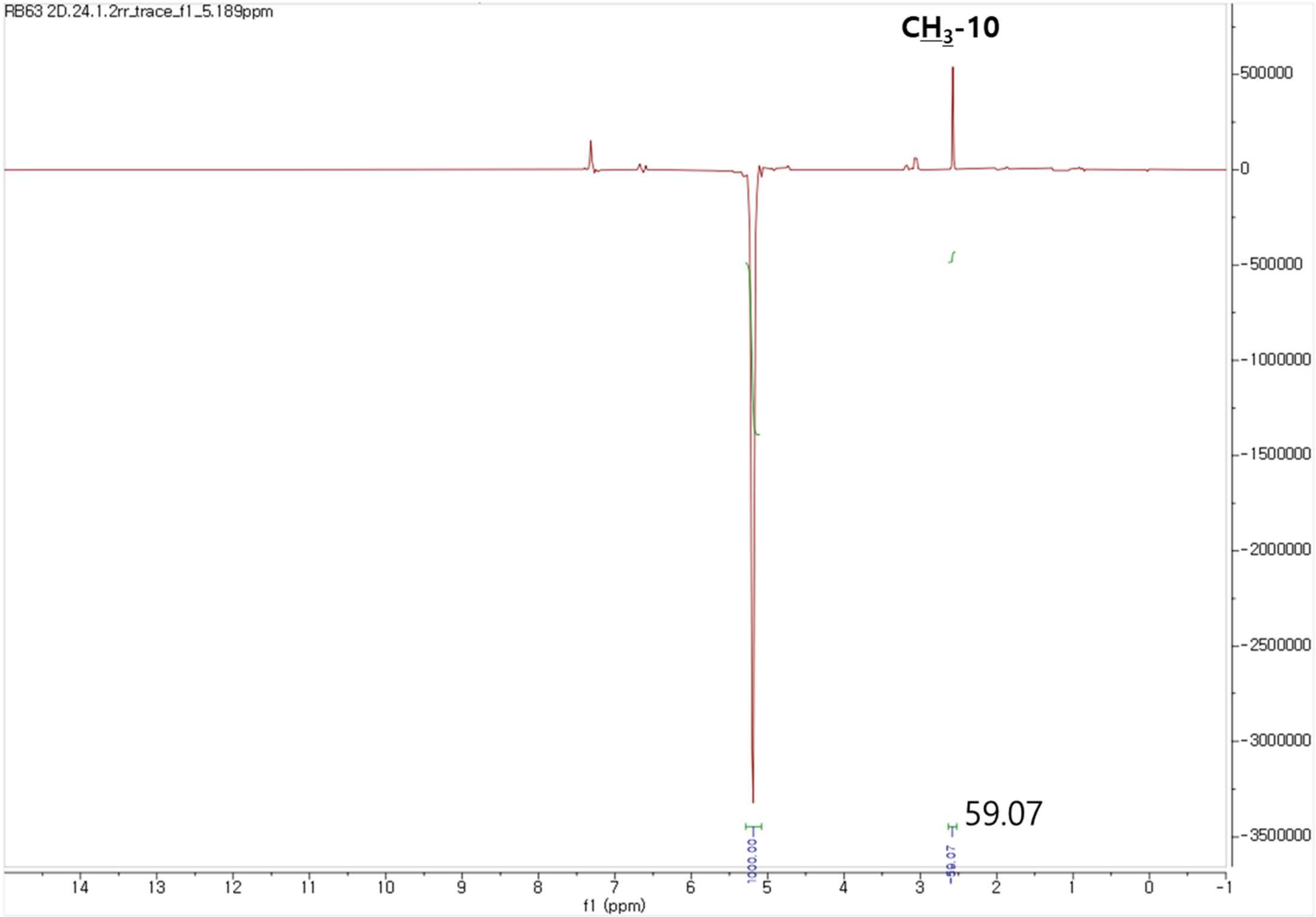

Figure S18. HR-FABMS data of **3**

[Mass Spectrum]
Data : FAB-A375 Date : 02-Jun-2020 14:37
RT : 1.99 min Scan# : (48,53)
Elements : C 100/0, H 100/0, O 10/0
Mass Tolerance : 1000ppm, 5mmu if m/z < 5, 10mmu if m/z > 10
Unsaturation (U.S.) : 0.0 - 20.0

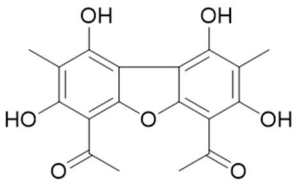

3

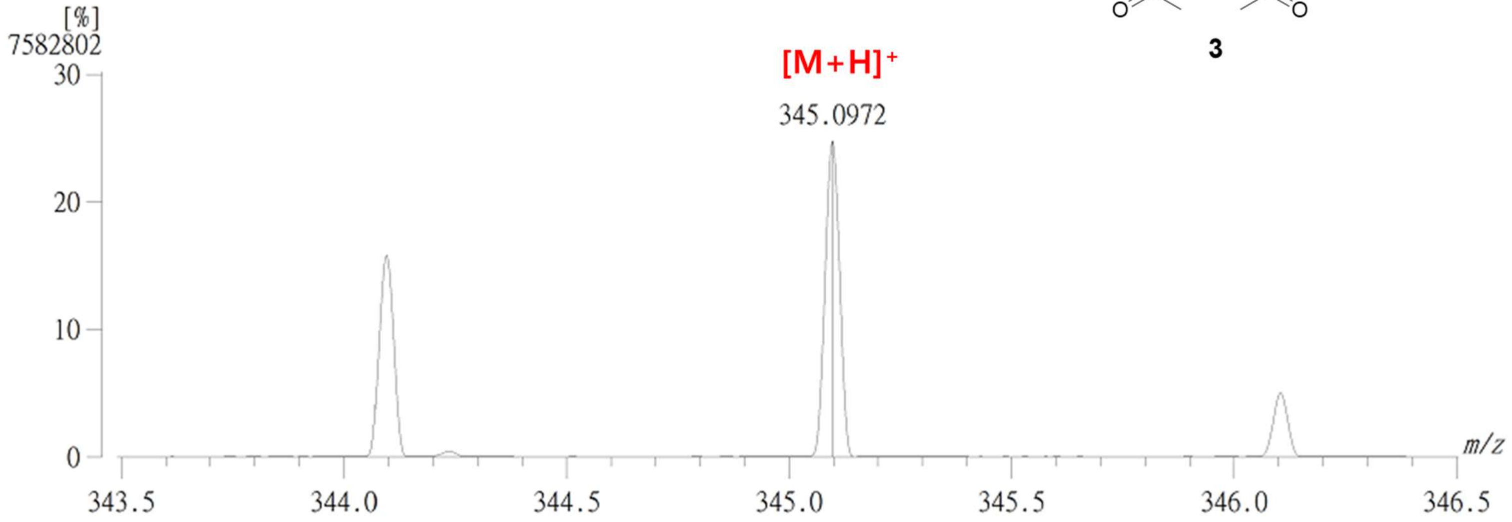

	Observed m/z	Int%	Err[ppm / mmu]	U.S.	Composition
1	345.0972	24.81	+16.4 / +5.6	19.5	C25 H13 O2
2			-0.7 / -0.2	10.5	C18 H17 O7

Figure S19. UV spectrum of **3**

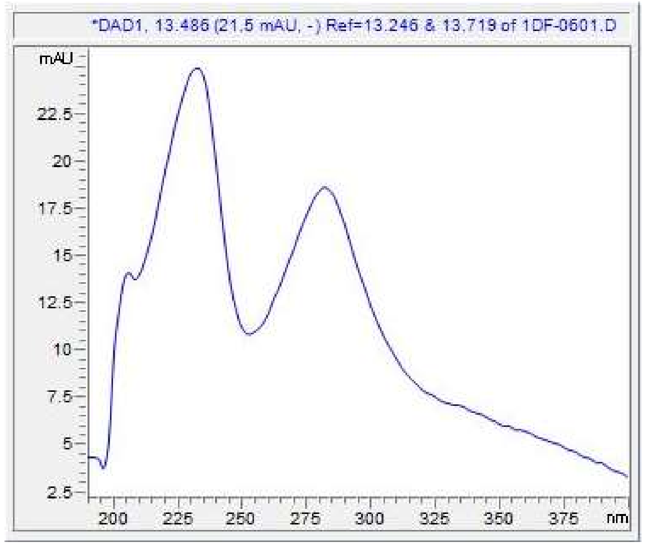

Figure S20. IR spectrum of **3**

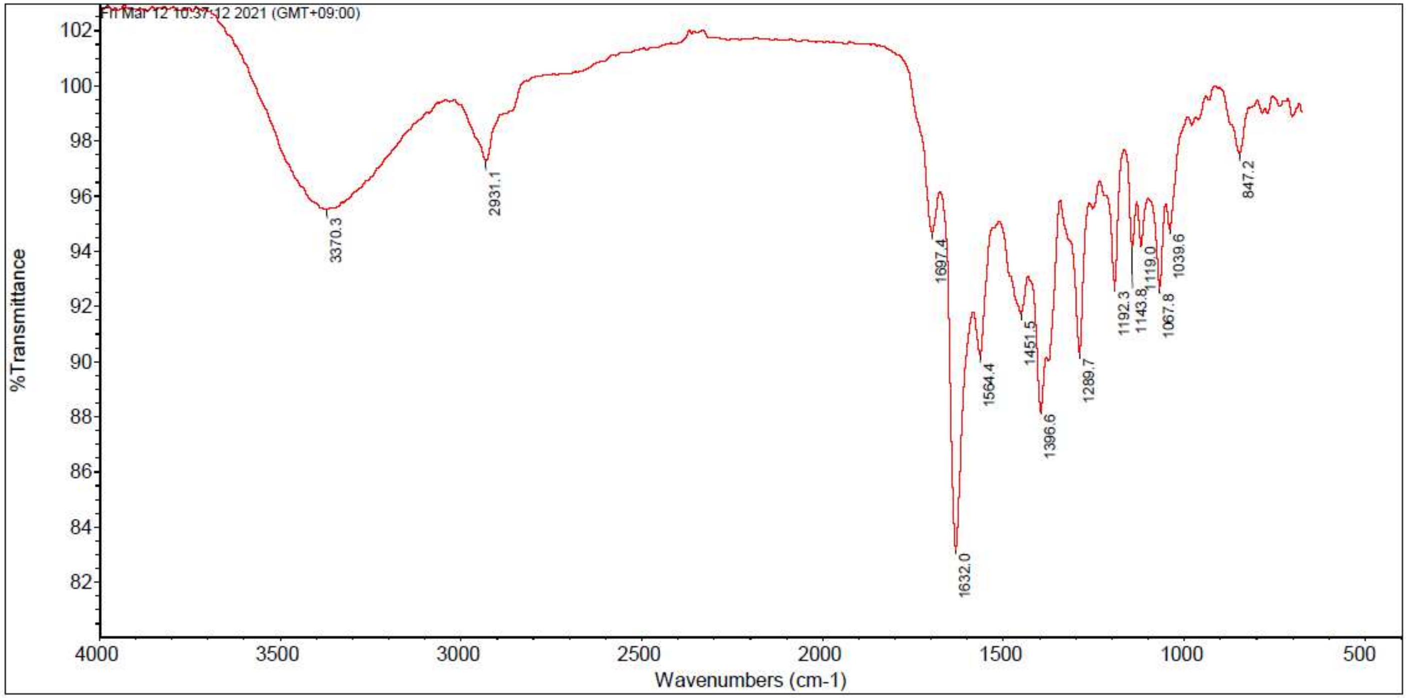

Fri Mar 12 10:38:22 2021 (GMT+09:00)
FIND PEAKS:

Spectrum: Fri Mar 12 10:37:12 2021 (GMT+09:00)
Region: 4000.0 400.0
Absolute threshold: 98.502
Sensitivity: 59
Peak list:

Position:	847.2	Intensity:	97.553
Position:	1039.6	Intensity:	94.746
Position:	1067.8	Intensity:	92.594
Position:	1119.0	Intensity:	94.185
Position:	1143.8	Intensity:	94.040
Position:	1192.3	Intensity:	92.614
Position:	1289.7	Intensity:	90.212
Position:	1396.6	Intensity:	88.162

Figure S21. ^{1}H NMR spectrum of **3** (DMSO-d_6, 600 MHz)

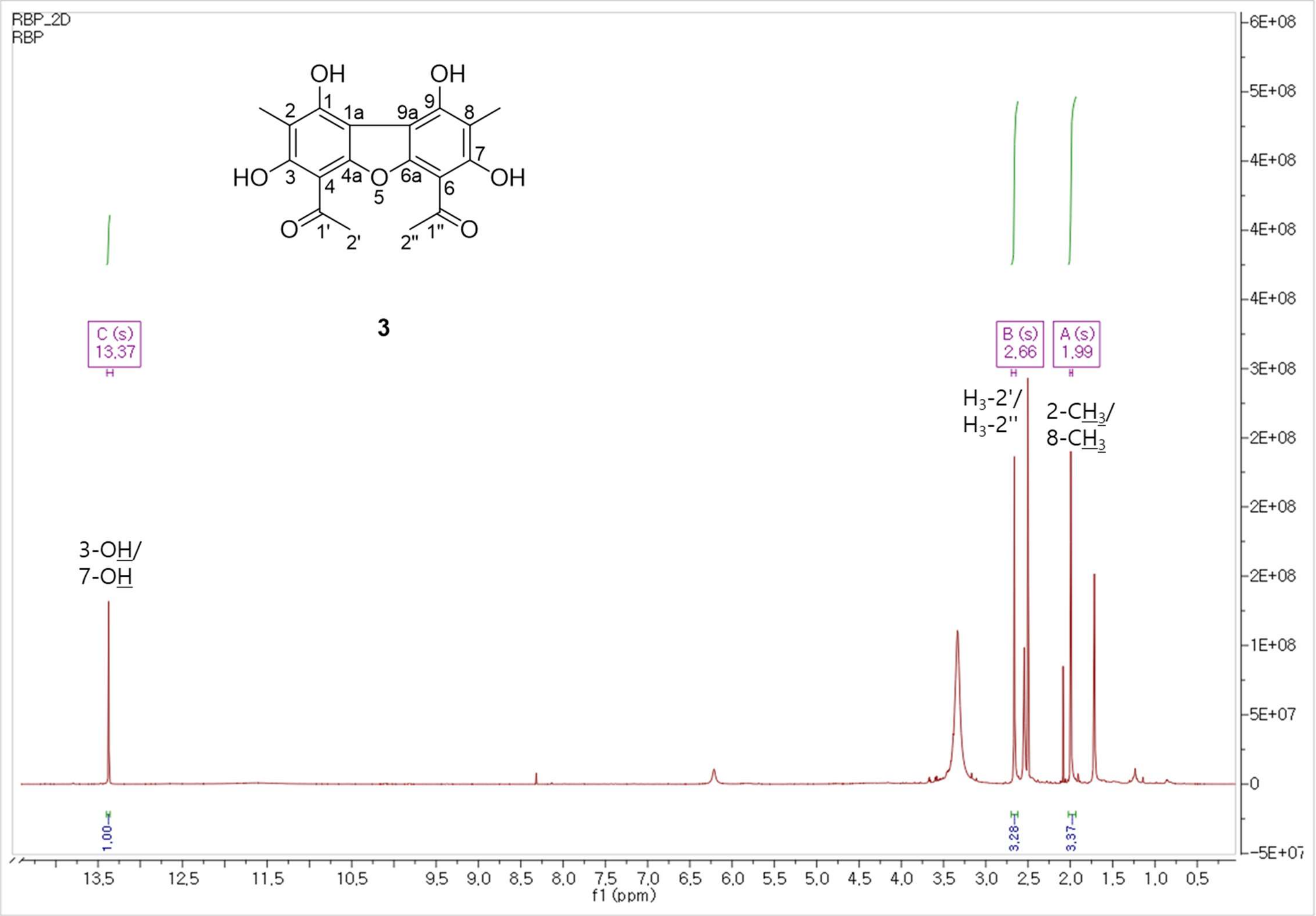

Figure S22. ^{13}C NMR spectrum of **3** (DMSO-d_6, 150 MHz)

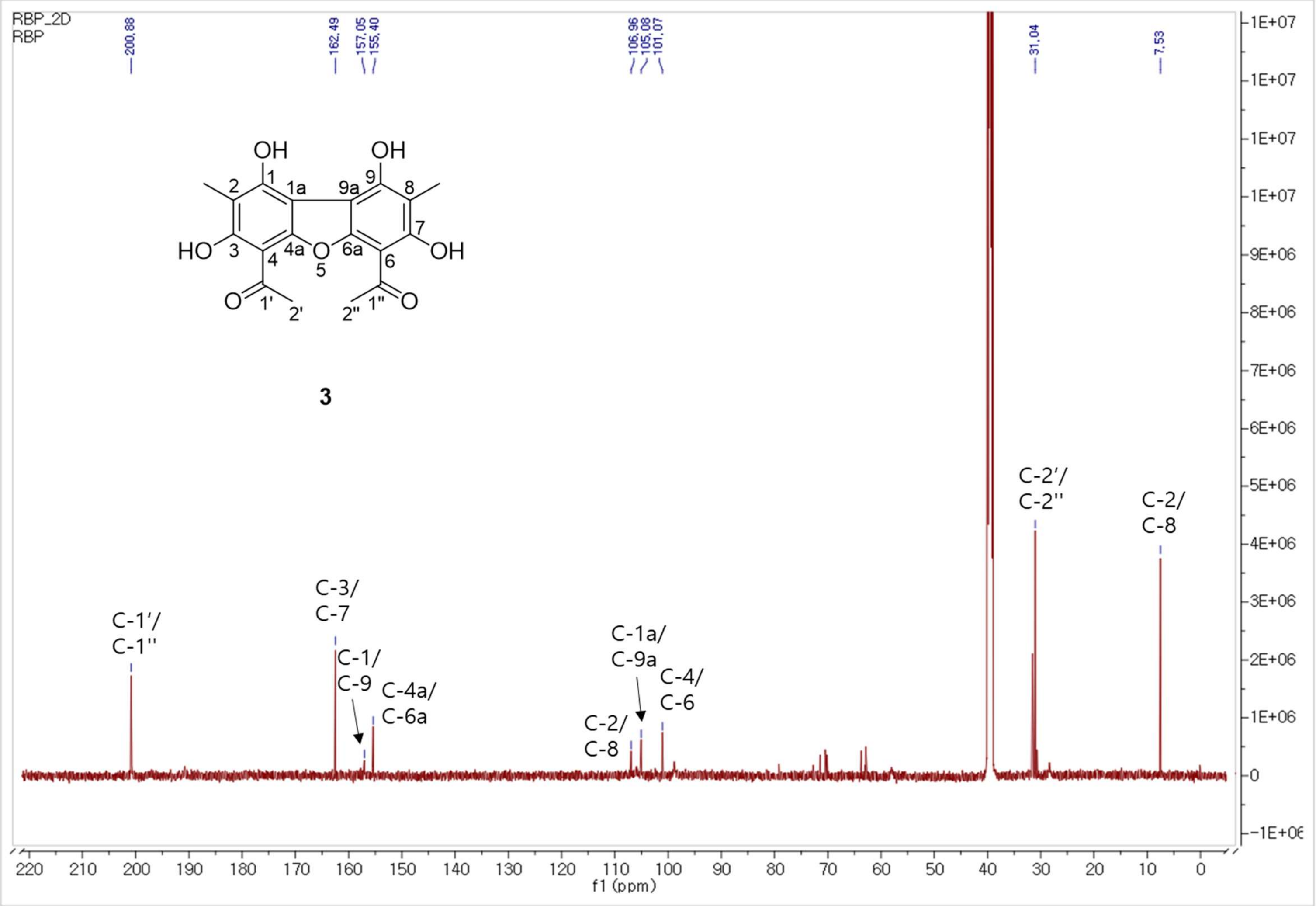

Figure S23. NOESY spectrum of **3** (DMSO-d_6)

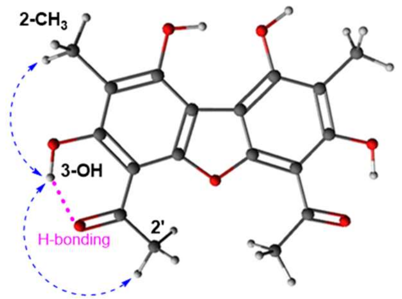

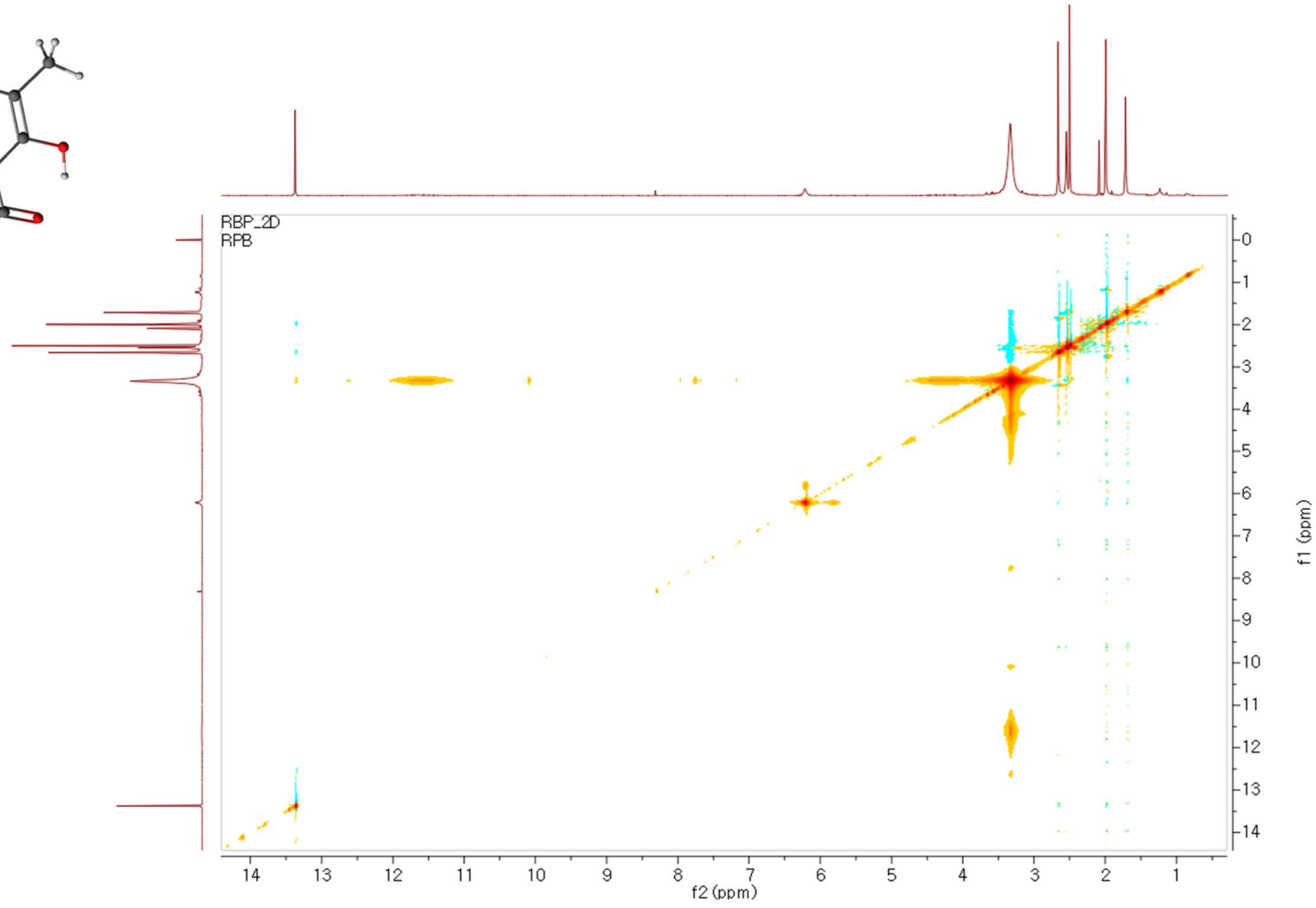

Figure S24. HSQC spectrum of **3** (DMSO-d_6)

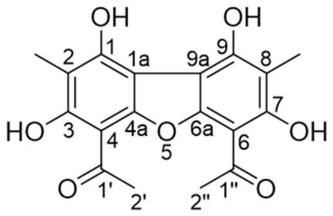

3

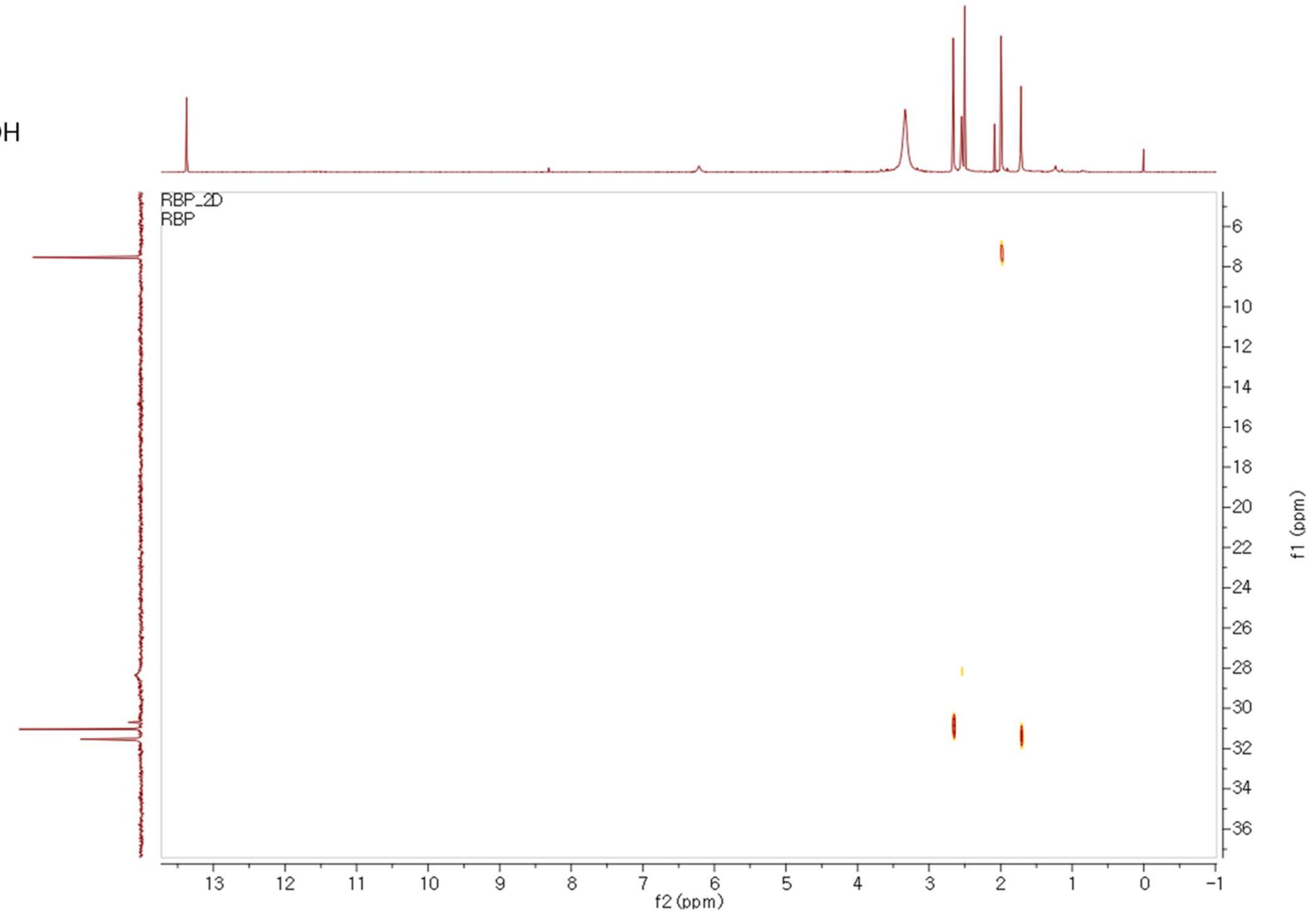

Figure S25. HMBC spectrum of **3** (DMSO-d_6)

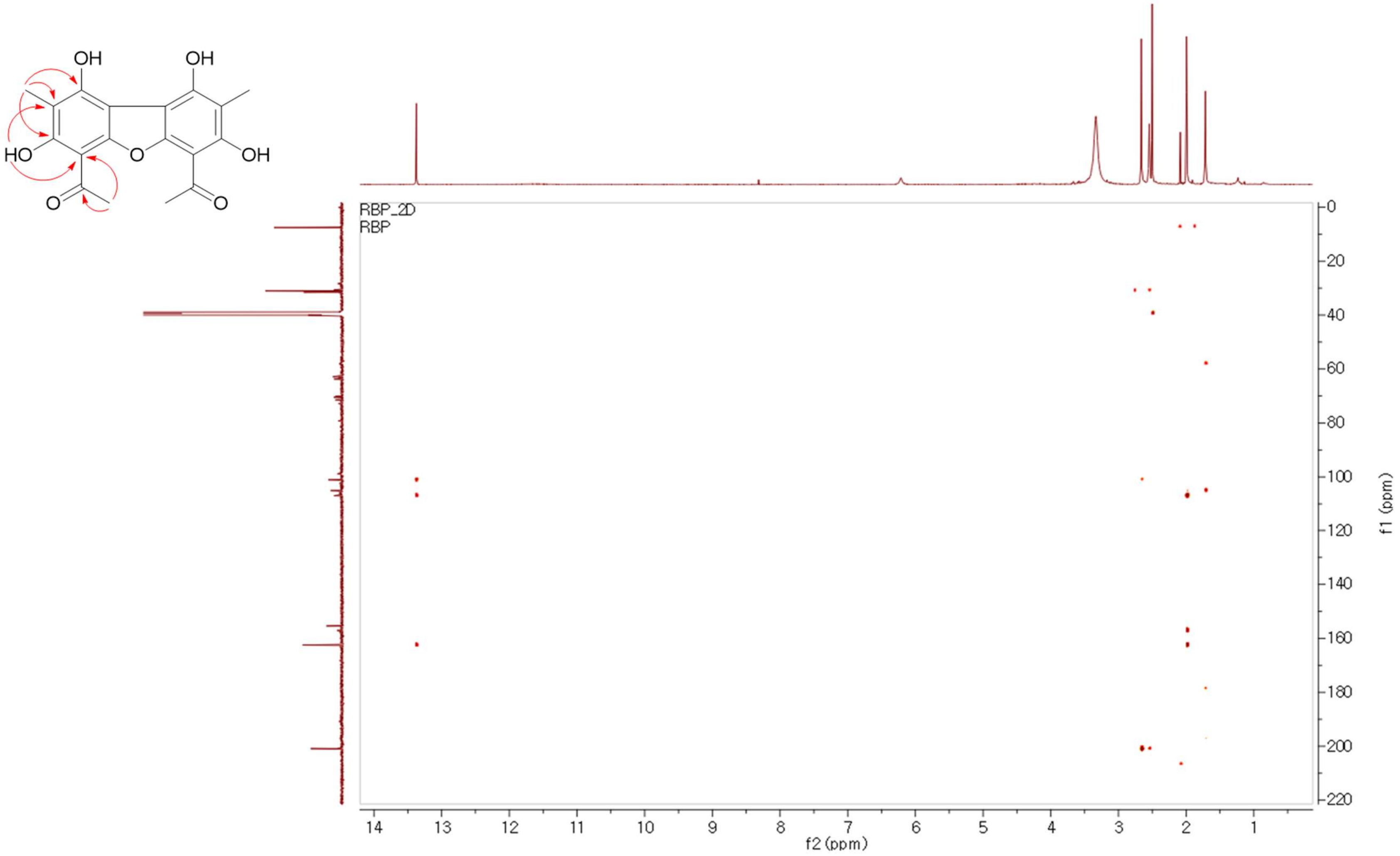

Table S1. IC_{50} values of compounds **1-3** against *E. coli*

Compounds	IC_{50} (ug/mL)
**Apramycin**	2.19
**1**	30.06
**2**	28.44
**3**	18.34

Apramycin was used as positive control.

Table S2. Cytotoxicity of compounds **1** and **2** against HCT116 cells

Compounds	IC_{50} (μM)[a]
**1**	25.85 ± 3.32
**2**	20.72 ± 1.20

[a]IC_{50} value of each compound was defined as the concentration (μM) that caused 50% inhibition of NO production in LPS-activated RAW264.7 mouse macrophages. The results are the averages of three independent experiments, and the data are expressed as mean ± SD.

Table S3. NO inhibition of compounds **1** and **2** against LPS-induced RAW264.7 cells

Compounds	IC_{50} (μM)[a]
**1**	7.0 ± 0.004
**2**	5.5 ± 0.006

[a]IC_{50} value of each compound was defined as the concentration (μM) that caused 50% inhibition of NO production in LPS-activated RAW264.7 mouse macrophages. The results are the averages of three independent experiments, and the data are expressed as mean ± SD.

Table S4. Gibbs free energies and Boltzmann distribution of conformer **1a**

Conformers	B3LYP/6-31+G(d,p) Gibbs free energy (298.15 K)		
	G (Hartree)	ΔG (kcal/mol)	Boltzmann distribution(%)
**1a**-1	-1690.059209	0.00	78.5%
**1a**-3	-1690.057693	0.95	15.7%
**1a**-2	-1690.056746	1.55	5.8%

Table S5. Gibbs free energies and Boltzmann distribution of conformer **1b**

Conformers	B3LYP/6-31+G(d,p) Gibbs free energy (298.15 K)		
	G (Hartree)	ΔG (kcal/mol)	Boltzmann distribution(%)
**1b**-2	-1690.060353	-0.89	32.1%
**1b**-11	-1690.059722	-0.49	16.4%
**1b**-10	-1690.059066	-0.08	8.2%
**1b**-6	-1690.059027	-0.05	7.9%
**1b**-12	-1690.059033	-0.06	7.9%
**1b**-8	-1690.059017	-0.05	7.8%
**1b**-1	-1690.058941	0.00	7.2%
**1b**-5	-1690.058296	0.40	3.6%
**1b**-13	-1690.057805	0.71	2.2%
**1b**-3	-1690.05763	0.82	1.8%
**1b**-7	-1690.057629	0.82	1.8%
**1b**-4	-1690.057569	0.86	1.7%
**1b**-9	-1690.056402	1.59	0.5%
**1b**-14	-1690.05703	1.20	0.9%

Coordinates of the conformers

1a-1

Center Number	Coordinates (Angstroms)			Atom	Atomic Number	Atomic Type
	X	Y	Z			
1	2.23594536	3.65872422	-7.95092901	o	8	0
2	4.50170026	4.14706152	-8.15217258	c	6	0
3	5.31461643	6.56526691	-8.56158754	n	7	0
4	7.95187274	7.38060085	-8.70317217	c	6	0
5	3.40680525	8.58409097	-8.39199216	c	6	0
6	1.52545907	8.70376266	-10.61654914	c	6	0
7	2.68438724	9.31430102	-13.16204582	c	6	0
8	2.28722936	7.70719253	-15.2288718	c	6	0
9	3.24946253	8.28996326	-17.6185812	c	6	0
10	4.64532899	10.49855463	-17.98067915	c	6	0
11	5.05223731	12.12402329	-15.93995263	c	6	0
12	4.07182778	11.5401337	-13.56285645	c	6	0
13	1.96540054	8.54217752	-5.8585993	c	6	0
14	3.46735573	7.80748379	-3.93265042	o	8	0
15	2.32836384	7.60209438	-1.41187906	c	6	0
16	3.67187857	9.47035977	0.33703669	c	6	0
17	3.30749357	12.26992971	-0.35973115	c	6	0
18	0.5833265	13.240558	-0.10308026	c	6	0
19	0.29662985	16.1036113	-0.52116037	c	6	0
20	0.8936889	16.98097347	-3.21928864	c	6	0
21	2.43543076	4.77852084	-0.63123001	c	6	0

22	0.71659007	4.23315009	1.64410359	c	6	0
23	5.14741994	3.91738298	-0.05665413	c	6	0
24	6.72272499	2.79025173	-2.22496393	c	6	0
25	9.32211586	1.88274514	-1.30312739	c	6	0
26	6.07044268	4.22121264	2.02593752	o	8	0
27	-0.19314175	9.26875391	-5.63267203	o	8	0
28	6.43056944	1.95074326	-7.84089351	c	6	0
29	6.81933438	0.47990068	-10.35394508	c	6	0
30	8.59074745	1.744401	-12.20956756	c	6	0
31	11.21211166	1.40652744	-11.98142196	c	6	0
32	12.87937328	2.60729046	-13.63434745	c	6	0
33	11.94721335	4.16911795	-15.54812679	c	6	0
34	9.34381095	4.51050382	-15.8012062	c	6	0
35	7.68105839	3.29933566	-14.14813639	c	6	0
36	5.47650684	0.180326	-5.95580068	n	7	0
37	5.25075791	0.5937721	-3.40141568	c	6	0
38	3.99905852	-0.82904987	-2.06816108	o	8	0
39	4.4825435	10.35583442	-8.3474059	h	1	0
40	0.33785765	8.10341436	-1.61531458	h	1	0
41	1.72458332	3.72883761	-2.26412894	h	1	0
42	6.94998702	4.32976449	-3.59416588	h	1	0
43	8.26604574	2.63540178	-7.1955143	h	1	0
44	9.19167093	5.81266405	-9.18095684	h	1	0
45	8.14853613	8.77851693	-10.2122459	h	1	0
46	8.56900406	8.22973426	-6.91301164	h	1	0
47	0.12709201	10.14493023	-10.1159038	h	1	0

48	0.51500957	6.90776613	-10.69420794	h	1	0
49	1.18137198	5.99454385	-14.96588069	h	1	0
50	2.89633604	7.0224628	-19.19585904	h	1	0
51	5.39121931	10.96176864	-19.83663745	h	1	0
52	6.1150796	13.8613023	-16.20492438	h	1	0
53	4.3659463	12.85523304	-12.00933099	h	1	0
54	2.98056916	9.14037654	2.26299295	h	1	0
55	5.6932443	9.0246683	0.37686588	h	1	0
56	3.99839348	12.58189849	-2.28976086	h	1	0
57	4.54027168	13.39671161	0.87364749	h	1	0
58	-0.11263196	12.77091756	1.79723382	h	1	0
59	-0.64614451	12.24724451	-1.44539787	h	1	0
60	1.50854593	17.12252598	0.82384584	h	1	0
61	-1.6515386	16.64301316	-0.057441	h	1	0
62	0.51343809	19.00306187	-3.4479206	h	1	0
63	2.88073364	16.66899689	-3.70948812	h	1	0
64	-0.26203463	15.95916268	-4.60234426	h	1	0
65	1.46584094	5.08469252	3.36880963	h	1	0
66	0.59967013	2.19254825	1.94592866	h	1	0
67	-1.19507071	4.94959855	1.30742639	h	1	0
68	10.47839016	1.21732228	-2.88439675	h	1	0
69	10.31607495	3.41247202	-0.33839289	h	1	0
70	9.0887382	0.33084245	0.0390177	h	1	0
71	7.57990837	-1.37386535	-9.83270939	h	1	0
72	4.95995291	0.1629243	-11.21160702	h	1	0
73	11.94899249	0.17961338	-10.50474933	h	1	0

74	14.90210313	2.31460624	-13.43536926	h	1	0
75	13.24209194	5.10560022	-16.83720137	h	1	0
76	8.59414522	5.72763521	-17.27403678	h	1	0
77	5.65997374	3.58602675	-14.35990869	h	1	0
78	4.18423731	-1.08889639	-6.5810889	h	1	0

1a-2

Center Number	Coordinates (Angstroms)			Atom	Atomic Number	Atomic Type
	X	Y	Z			
1	3.60752337	2.5650867	-8.32691019	o	8	0
2	5.76908323	3.39193545	-8.09869767	c	6	0
3	6.32445871	5.84892776	-8.661493	n	7	0
4	8.82555359	7.01434021	-8.64994345	c	6	0
5	4.20645074	7.50257454	-9.39398628	c	6	0
6	3.27676582	7.04589867	-12.13600201	c	6	0
7	2.28528919	9.39873733	-13.44083533	c	6	0
8	3.92882191	10.9109928	-14.86937541	c	6	0
9	3.06823908	13.08991005	-16.08132939	c	6	0
10	0.53239205	13.79121079	-15.88621328	c	6	0
11	-1.12370632	12.29992181	-14.47281681	c	6	0
12	-0.25737533	10.12356109	-13.25931053	c	6	0
13	2.03984388	7.37316022	-7.45722917	c	6	0
14	2.95927153	7.50436571	-5.07784126	o	8	0
15	1.17307204	7.2491724	-2.97749744	c	6	0
16	1.33855122	9.63308018	-1.35759515	c	6	0
17	0.48563153	12.02844513	-2.75710985	c	6	0
18	0.33652976	14.39178401	-1.06943172	c	6	0

19	2.84358348	15.27306912	0.11484823	c	6	0
20	4.86281609	16.05735472	-1.81113567	c	6	0
21	1.77548022	4.70644355	-1.65336208	c	6	0
22	-0.25606073	3.98572332	0.29176707	c	6	0
23	4.3887424	4.75416611	-0.39009519	c	6	0
24	6.64690418	3.6470352	-1.85103983	c	6	0
25	9.06710891	3.65072165	-0.24687229	c	6	0
26	4.69196468	5.733999	1.66682889	o	8	0
27	-0.17985309	7.32672229	-8.00005502	o	8	0
28	7.84948171	1.60912865	-7.05438437	c	6	0
29	8.73018397	-0.25885601	-9.13333006	c	6	0
30	10.07326863	0.96105671	-11.34356052	c	6	0
31	8.74172455	1.65908791	-13.52538774	c	6	0
32	9.97793442	2.82378787	-15.54524019	c	6	0
33	12.56961866	3.3047954	-15.41413992	c	6	0
34	13.92007344	2.60281248	-13.25790088	c	6	0
35	12.67931537	1.43649936	-11.24282662	c	6	0
36	6.84367975	0.1094793	-4.97141167	n	7	0
37	5.99222366	0.9516258	-2.66742804	c	6	0
38	4.82223726	-0.45716608	-1.24683125	o	8	0
39	4.94356747	9.43423228	-9.26183172	h	1	0
40	-0.71336137	7.09076287	-3.80577206	h	1	0
41	1.77791524	3.29256379	-3.16318261	h	1	0
42	6.88476034	4.87450967	-3.50357807	h	1	0
43	9.48443361	2.66430229	-6.36519167	h	1	0
44	10.31393205	5.5974712	-8.6102234	h	1	0

45	9.0855121	8.09986064	-10.39227676	h	1	0
46	9.0485519	8.29786754	-7.03641879	h	1	0
47	1.84822722	5.55751397	-12.09213721	h	1	0
48	4.89704654	6.32099597	-13.20070686	h	1	0
49	5.90336151	10.36275462	-15.05326829	h	1	0
50	4.37207817	14.22844029	-17.18702879	h	1	0
51	-0.14844563	15.48128904	-16.83331358	h	1	0
52	-3.10198248	12.82911159	-14.31529892	h	1	0
53	-1.53901049	8.98203412	-12.13819755	h	1	0
54	0.13874555	9.34596763	0.30895715	h	1	0
55	3.26821295	9.84488925	-0.64225912	h	1	0
56	-1.38612464	11.69637955	-3.58947873	h	1	0
57	1.7590306	12.38201188	-4.35414044	h	1	0
58	-1.03005716	14.03765087	0.45338872	h	1	0
59	-0.45012944	15.95105523	-2.19331775	h	1	0
60	3.60347431	13.78858035	1.348532	h	1	0
61	2.43944483	16.88311051	1.35860984	h	1	0
62	6.55173807	16.78746533	-0.86278796	h	1	0
63	5.45739527	14.46835035	-2.99694731	h	1	0
64	4.15741544	17.54458177	-3.07073882	h	1	0
65	-0.17949523	5.22366003	1.94227887	h	1	0
66	0.06324163	2.04776299	0.93320467	h	1	0
67	-2.14450078	4.08992914	-0.54772322	h	1	0
68	10.68787392	2.98698957	-1.34854298	h	1	0
69	9.46131715	5.55232841	0.45131904	h	1	0
70	8.83066202	2.41015803	1.38704941	h	1	0

71	9.97238718	-1.64228523	-8.22149725	h	1	0
72	7.04878927	-1.27088485	-9.79756169	h	1	0
73	6.72658576	1.27630041	-13.64239524	h	1	0
74	8.9184526	3.33913658	-17.227122	h	1	0
75	13.53377577	4.20263845	-16.98861873	h	1	0
76	15.9414264	2.94736627	-13.15058071	h	1	0
77	13.74792978	0.86988965	-9.57961086	h	1	0
78	6.07586433	-1.58215808	-5.43725497	h	1	0

1a-3

Center Number	Coordinates (Angstroms)			Atom	Atomic Number	Atomic Type
	X	Y	Z			
1	2.23594536	3.65872422	-7.95092901	o	8	0
2	4.50170026	4.14706152	-8.15217258	c	6	0
3	5.31461643	6.56526691	-8.56158754	n	7	0
4	7.95187274	7.38060085	-8.70317217	c	6	0
5	3.40680525	8.58409097	-8.39199216	c	6	0
6	1.52545907	8.70376266	-10.61654914	c	6	0
7	2.68438724	9.31430102	-13.16204582	c	6	0
8	2.28722936	7.70719253	-15.2288718	c	6	0
9	3.24946253	8.28996326	-17.6185812	c	6	0
10	4.64532899	10.49855463	-17.98067915	c	6	0
11	5.05223731	12.12402329	-15.93995263	c	6	0
12	4.07182778	11.5401337	-13.56285645	c	6	0
13	1.96540054	8.54217752	-5.8585993	c	6	0
14	3.46735573	7.80748379	-3.93265042	o	8	0
15	2.32836384	7.60209438	-1.41187906	c	6	0

16	3.67187857	9.47035977	0.33703669	c	6	0
17	3.30749357	12.26992971	-0.35973115	c	6	0
18	0.5833265	13.240558	-0.10308026	c	6	0
19	0.29662985	16.1036113	-0.52116037	c	6	0
20	0.8936889	16.98097347	-3.21928864	c	6	0
21	2.43543076	4.77852084	-0.63123001	c	6	0
22	0.71659007	4.23315009	1.64410359	c	6	0
23	5.14741994	3.91738298	-0.05665413	c	6	0
24	6.72272499	2.79025173	-2.22496393	c	6	0
25	9.32211586	1.88274514	-1.30312739	c	6	0
26	6.07044268	4.22121264	2.02593752	o	8	0
27	-0.19314175	9.26875391	-5.63267203	o	8	0
28	6.43056944	1.95074326	-7.84089351	c	6	0
29	6.81933438	0.47990068	-10.35394508	c	6	0
30	8.59074745	1.744401	-12.20956756	c	6	0
31	11.21211166	1.40652744	-11.98142196	c	6	0
32	12.87937328	2.60729046	-13.63434745	c	6	0
33	11.94721335	4.16911795	-15.54812679	c	6	0
34	9.34381095	4.51050382	-15.8012062	c	6	0
35	7.68105839	3.29933566	-14.14813639	c	6	0
36	5.47650684	0.180326	-5.95580068	n	7	0
37	5.25075791	0.5937721	-3.40141568	c	6	0
38	3.99905852	-0.82904987	-2.06816108	o	8	0
39	4.4825435	10.35583442	-8.3474059	h	1	0
40	0.33785765	8.10341436	-1.61531458	h	1	0
41	1.72458332	3.72883761	-2.26412894	h	1	0

42	6.94998702	4.32976449	-3.59416588	h	1	0
43	8.26604574	2.63540178	-7.1955143	h	1	0
44	9.19167093	5.81266405	-9.18095684	h	1	0
45	8.14853613	8.77851693	-10.2122459	h	1	0
46	8.56900406	8.22973426	-6.91301164	h	1	0
47	0.12709201	10.14493023	-10.1159038	h	1	0
48	0.51500957	6.90776613	-10.69420794	h	1	0
49	1.18137198	5.99454385	-14.96588069	h	1	0
50	2.89633604	7.0224628	-19.19585904	h	1	0
51	5.39121931	10.96176864	-19.83663745	h	1	0
52	6.1150796	13.8613023	-16.20492438	h	1	0
53	4.3659463	12.85523304	-12.00933099	h	1	0
54	2.98056916	9.14037654	2.26299295	h	1	0
55	5.6932443	9.0246683	0.37686588	h	1	0
56	3.99839348	12.58189849	-2.28976086	h	1	0
57	4.54027168	13.39671161	0.87364749	h	1	0
58	-0.11263196	12.77091756	1.79723382	h	1	0
59	-0.64614451	12.24724451	-1.44539787	h	1	0
60	1.50854593	17.12252598	0.82384584	h	1	0
61	-1.6515386	16.64301316	-0.057441	h	1	0
62	0.51343809	19.00306187	-3.4479206	h	1	0
63	2.88073364	16.66899689	-3.70948812	h	1	0
64	-0.26203463	15.95916268	-4.60234426	h	1	0
65	1.46584094	5.08469252	3.36880963	h	1	0
66	0.59967013	2.19254825	1.94592866	h	1	0
67	-1.19507071	4.94959855	1.30742639	h	1	0

68	10.47839016	1.21732228	-2.88439675	h	1	0
69	10.31607495	3.41247202	-0.33839289	h	1	0
70	9.0887382	0.33084245	0.0390177	h	1	0
71	7.57990837	-1.37386535	-9.83270939	h	1	0
72	4.95995291	0.1629243	-11.21160702	h	1	0
73	11.94899249	0.17961338	-10.50474933	h	1	0
74	14.90210313	2.31460624	-13.43536926	h	1	0
75	13.24209194	5.10560022	-16.83720137	h	1	0
76	8.59414522	5.72763521	-17.27403678	h	1	0
77	5.65997374	3.58602675	-14.35990869	h	1	0
78	4.18423731	-1.08889639	-6.5810889	h	1	0

1b-1

Center Number	Coordinates (Angstroms)			Atom	Atomic Number	Atomic Type
	X	Y	Z			
1	2.27276234	3.20952597	-8.21263714	o	8	0
2	4.4803232	3.93369703	-8.33389567	c	6	0
3	5.04823663	6.40983271	-8.80277436	n	7	0
4	7.58581902	7.50188129	-8.86853178	c	6	0
5	2.92816103	8.21378978	-8.78882515	c	6	0
6	1.15807636	8.06776758	-11.09991545	c	6	0
7	2.36848226	8.73604872	-13.6066877	c	6	0
8	2.24719211	7.04505991	-15.64114769	c	6	0
9	3.25923135	7.66926375	-17.99957555	c	6	0
10	4.42836678	10.00569517	-18.36212635	c	6	0
11	4.55910389	11.71523879	-16.35394871	c	6	0
12	3.52956016	11.08765531	-14.00905682	c	6	0

13	1.37719662	8.07859212	-6.32484342	c	6	0
14	2.87169559	7.61809041	-4.30541363	o	8	0
15	1.64971718	7.38310753	-1.82901824	c	6	0
16	2.70165024	9.44820272	-0.10648317	c	6	0
17	2.01195736	12.13340301	-0.9595966	c	6	0
18	2.98562074	14.17945032	0.84860661	c	6	0
19	2.327067	16.8734648	0.00191508	c	6	0
20	3.30056038	18.91052648	1.81487773	c	6	0
21	2.00857537	4.61636107	-0.93223121	c	6	0
22	0.25378432	3.97468725	1.28921262	c	6	0
23	4.76745132	4.0716845	-0.21187052	c	6	0
24	6.54976196	3.04569828	-2.26737088	c	6	0
25	9.18795705	2.45970063	-1.20569147	c	6	0
26	5.55827133	4.54653646	1.89328879	o	8	0
27	-0.86274286	8.52453584	-6.21970242	o	8	0
28	6.6149329	1.9708122	-7.85126569	c	6	0
29	7.26900451	0.45927162	-10.28403909	c	6	0
30	8.97314926	1.84134797	-12.1188203	c	6	0
31	7.98606511	3.22857144	-14.14499184	c	6	0
32	9.57961295	4.55315527	-15.77916908	c	6	0
33	12.19209066	4.49448681	-15.41916871	c	6	0
34	13.20310022	3.10251478	-13.41646184	c	6	0
35	11.6040123	1.78789746	-11.78279854	c	6	0
36	5.76820655	0.17842813	-5.93529647	n	7	0
37	5.3757705	0.66189533	-3.41295159	c	6	0
38	4.21822782	-0.83594581	-2.07711846	o	8	0

39	3.80545092	10.09131089	-8.74971321	h	1	0
40	-0.3718843	7.68300305	-2.13109277	h	1	0
41	1.48684746	3.43938657	-2.54991622	h	1	0
42	6.67183653	4.55116317	-3.68768547	h	1	0
43	8.33705957	2.87165629	-7.15997267	h	1	0
44	9.00743702	6.06087821	-9.22238142	h	1	0
45	7.70279512	8.85217994	-10.42829174	h	1	0
46	8.02169973	8.48439546	-7.09317456	h	1	0
47	-0.41260806	9.35857098	-10.7100334	h	1	0
48	0.35314697	6.17057217	-11.16767395	h	1	0
49	1.31837407	5.23020689	-15.3792134	h	1	0
50	3.12222275	6.33257971	-19.55320029	h	1	0
51	5.21147421	10.50054025	-20.19444521	h	1	0
52	5.44398976	13.54936232	-16.62022566	h	1	0
53	3.60531514	12.46434454	-12.48306407	h	1	0
54	1.96578719	9.11523983	1.80230801	h	1	0
55	4.7595806	9.25632663	0.03777054	h	1	0
56	-0.0499266	12.29546547	-1.1432323	h	1	0
57	2.78617951	12.47356442	-2.85514777	h	1	0
58	5.04758482	14.00556907	1.04145815	h	1	0
59	2.20713094	13.84889813	2.74714089	h	1	0
60	0.2667904	17.04378843	-0.19110796	h	1	0
61	3.10695574	17.20368979	-1.89474973	h	1	0
62	2.80072192	20.80919878	1.16012484	h	1	0
63	2.50098691	18.66975844	3.71094091	h	1	0
64	5.36281165	18.82946502	1.99527968	h	1	0

65	0.82816904	4.96513721	3.00731545	h	1	0
66	0.33808512	1.9459929	1.67137619	h	1	0
67	-1.70465684	4.47024666	0.84242533	h	1	0
68	10.47879573	1.86973094	-2.71077491	h	1	0
69	9.96790381	4.12100455	-0.26221478	h	1	0
70	9.06309274	0.93911038	0.18644552	h	1	0
71	8.20077184	-1.28008312	-9.65714099	h	1	0
72	5.49341641	-0.0900366	-11.20029911	h	1	0
73	5.95624452	3.29457248	-14.44110577	h	1	0
74	8.7675158	5.63683022	-17.32142173	h	1	0
75	13.43392294	5.51836307	-16.69391066	h	1	0
76	15.2356482	3.03011679	-13.13293758	h	1	0
77	12.40419902	0.69337032	-10.23672414	h	1	0
78	4.64616998	-1.24306842	-6.56181599	h	1	0

1b-2

Center Number	Coordinates (Angstroms)			Atom	Atomic Number	Atomic Type
	X	Y	Z			
1	2.50814438	3.80598956	-8.19939971	o	8	0
2	4.42930462	4.97464499	-8.78829686	c	6	0
3	4.36243146	7.51135296	-9.27030234	n	7	0
4	6.53052587	9.07394582	-9.96576534	c	6	0
5	1.95642339	8.82425835	-8.81206214	c	6	0
6	-0.04554331	8.36119513	-10.88735191	c	6	0
7	0.84495439	9.16248526	-13.48360166	c	6	0
8	1.77672357	7.38231318	-15.21100586	c	6	0
9	2.64741892	8.12455456	-17.59000875	c	6	0

10	2.60311484	10.66996972	-18.2816975	c	6	0
11	1.66949211	12.46402717	-16.58436741	c	6	0
12	0.79631232	11.71243159	-14.21152943	c	6	0
13	0.86400996	8.25349774	-6.17589222	c	6	0
14	2.70785789	8.10101276	-4.41713882	o	8	0
15	1.9805233	7.38224979	-1.84209938	c	6	0
16	2.69043146	9.5438772	-0.06491335	c	6	0
17	1.19255311	11.98088387	-0.54053048	c	6	0
18	1.8664021	14.09752526	1.32218318	c	6	0
19	0.39260315	16.55153499	0.86445026	c	6	0
20	1.0768888	18.65837934	2.72915622	c	6	0
21	3.2039368	4.77389162	-1.29348156	c	6	0
22	2.08760657	3.54435156	1.08752171	c	6	0
23	6.08964513	4.97529408	-1.04752315	c	6	0
24	7.70753436	4.49089274	-3.41321289	c	6	0
25	10.547209	4.64841335	-2.82712599	c	6	0
26	7.0676866	5.61042179	0.93307762	o	8	0
27	-1.37855679	8.15215066	-5.74328423	o	8	0
28	6.98174428	3.54543058	-8.95242036	c	6	0
29	7.25905542	2.38008836	-11.62687685	c	6	0
30	9.74985183	1.04178427	-12.0619424	c	6	0
31	11.94454912	2.42713848	-12.59660041	c	6	0
32	14.2565509	1.21418883	-12.95826666	c	6	0
33	14.40920717	-1.41626078	-12.7965535	c	6	0
34	12.23588719	-2.81842878	-12.28118246	c	6	0
35	9.92589565	-1.59542128	-11.92117228	c	6	0

36	7.07196278	1.56158018	-7.05494862	n	7	0
37	7.0601511	1.87767325	-4.47772852	c	6	0
38	6.66048947	0.09347902	-3.05490076	o	8	0
39	2.41591325	10.84601634	-8.78486062	h	1	0
40	-0.06942726	7.12239026	-1.84940833	h	1	0
41	2.74350836	3.59571091	-2.92925217	h	1	0
42	7.17334308	5.98005272	-4.75140365	h	1	0
43	8.57316141	4.82045266	-8.62547884	h	1	0
44	7.9989927	7.95094632	-10.87030195	h	1	0
45	5.92513327	10.49350242	-11.33761945	h	1	0
46	7.34039157	10.03864615	-8.31628865	h	1	0
47	-1.74314957	9.40544912	-10.33805084	h	1	0
48	-0.53517353	6.35689789	-10.86095022	h	1	0
49	1.79197338	5.39462569	-14.69038773	h	1	0
50	3.34854834	6.71253241	-18.90637168	h	1	0
51	3.27376758	11.25098095	-20.13315339	h	1	0
52	1.60548813	14.44705849	-17.11461295	h	1	0
53	0.04715094	13.11950913	-12.91223973	h	1	0
54	2.36481806	8.89888085	1.87772808	h	1	0
55	4.72256825	9.92066946	-0.2045307	h	1	0
56	-0.84167891	11.57991498	-0.42945741	h	1	0
57	1.54326977	12.63819457	-2.47741421	h	1	0
58	3.90441625	14.4903208	1.21181062	h	1	0
59	1.51859197	13.4430549	3.26383181	h	1	0
60	-1.64268463	16.15742852	0.97964599	h	1	0
61	0.73830131	17.20487499	-1.07652428	h	1	0

62	-0.0083428	20.38146437	2.35872666	h	1	0
63	0.69344792	18.08307723	4.68222676	h	1	0
64	3.08777146	19.14300331	2.60795985	h	1	0
65	2.67300015	4.55090725	2.79214678	h	1	0
66	2.76201333	1.59700923	1.23838662	h	1	0
67	0.02121728	3.51050539	0.99460941	h	1	0
68	11.67034998	4.42399607	-4.54947117	h	1	0
69	11.00205212	6.46326603	-1.95530861	h	1	0
70	11.07325803	3.15676257	-1.49985812	h	1	0
71	5.67116499	1.07864565	-11.90909296	h	1	0
72	7.01563804	3.90249434	-13.01171621	h	1	0
73	11.84130647	4.47586881	-12.74742834	h	1	0
74	15.93551483	2.3196972	-13.3766444	h	1	0
75	16.20748557	-2.36502804	-13.07997754	h	1	0
76	12.33394728	-4.8656715	-12.16212739	h	1	0
77	8.23879058	-2.7066619	-11.54246183	h	1	0
78	6.40030341	-0.15557195	-7.56839123	h	1	0

1b-3

Center Number	Coordinates (Angstroms)			Atom	Atomic Number	Atomic Type
	X	Y	Z			
1	2.26590601	3.26314473	-8.17229045	o	8	0
2	4.47576256	3.98332647	-8.27807991	c	6	0
3	5.05026632	6.46481456	-8.70742314	n	7	0
4	7.58931646	7.5541686	-8.7517153	c	6	0
5	2.93332876	8.27222295	-8.67548939	c	6	0
6	1.17248083	8.16206141	-10.99551301	c	6	0

7	2.39663346	8.85651057	-13.48849773	c	6	0
8	2.27779164	7.19136418	-15.54427686	c	6	0
9	3.30382352	7.84033043	-17.88994586	c	6	0
10	4.48524294	10.17572698	-18.21783401	c	6	0
11	4.61370811	11.8597492	-16.1879816	c	6	0
12	3.56976238	11.20780441	-13.85612515	c	6	0
13	1.37234011	8.10188018	-6.21987486	c	6	0
14	2.86008161	7.61609616	-4.20129541	o	8	0
15	1.62946751	7.33688651	-1.73372776	c	6	0
16	2.69870645	9.35275005	0.03559998	c	6	0
17	1.99519252	12.06150025	-0.72902587	c	6	0
18	3.00712486	14.04290807	1.13349178	c	6	0
19	2.24366819	16.77973836	0.5201836	c	6	0
20	3.36370889	17.81888396	-1.94327529	c	6	0
21	1.96126051	4.54819611	-0.89523847	c	6	0
22	0.1784659	3.868827	1.29227477	c	6	0
23	4.7089398	3.97028896	-0.15845559	c	6	0
24	6.50571235	2.98360094	-2.22034719	c	6	0
25	9.13627894	2.3772778	-1.15155017	c	6	0
26	5.47945695	4.38693456	1.96625855	o	8	0
27	-0.86853549	8.5435613	-6.11711376	o	8	0
28	6.60419104	2.00754524	-7.82198788	c	6	0
29	7.2663089	0.53962875	-10.27945036	c	6	0
30	8.9914756	1.946538	-12.07516085	c	6	0
31	11.61889914	1.87879029	-11.71581517	c	6	0
32	13.23712534	3.21575277	-13.31204952	c	6	0

33	12.24902716	4.64437521	-15.30036691	c	6	0
34	9.64024683	4.71662255	-15.68403187	c	6	0
35	8.02752856	3.37025387	-14.08694319	c	6	0
36	5.74551579	0.18197352	-5.94336617	n	7	0
37	5.33761867	0.62099457	-3.41544672	c	6	0
38	4.17098025	-0.89958494	-2.11361932	o	8	0
39	3.81385179	10.1472992	-8.60580225	h	1	0
40	-0.38866623	7.66184539	-2.03332133	h	1	0
41	1.44950527	3.41020311	-2.54399235	h	1	0
42	6.63751549	4.51364721	-3.61320221	h	1	0
43	8.32477225	2.89213176	-7.10641638	h	1	0
44	9.01206912	6.11270212	-9.0982489	h	1	0
45	7.72011041	8.9093845	-10.30617151	h	1	0
46	8.01209556	8.52996159	-6.96966923	h	1	0
47	-0.39504473	9.45395822	-10.59651689	h	1	0
48	0.36038064	6.26903975	-11.09033569	h	1	0
49	1.34072913	5.37709095	-15.30911647	h	1	0
50	3.1681847	6.52383749	-19.46080209	h	1	0
51	5.27950178	10.68964372	-20.04008734	h	1	0
52	5.50821478	13.69294756	-16.42711555	h	1	0
53	3.64446086	12.56485695	-12.31267447	h	1	0
54	1.99020469	8.96301828	1.94380867	h	1	0
55	4.75880848	9.15928354	0.14670352	h	1	0
56	-0.07090675	12.2305274	-0.85868252	h	1	0
57	2.72614668	12.44108082	-2.63121123	h	1	0
58	5.07987265	13.91235567	1.21717007	h	1	0

59	2.32978449	13.5735315	3.03940286	h	1	0
60	2.83437954	17.99785165	2.09196808	h	1	0
61	0.17221135	16.9082641	0.44441852	h	1	0
62	2.835861	19.80065598	-2.22282844	h	1	0
63	5.43397517	17.71880777	-1.92101798	h	1	0
64	2.70249601	16.76560316	-3.59768698	h	1	0
65	0.72497794	4.83464062	3.03338244	h	1	0
66	0.26326552	1.83494338	1.64555673	h	1	0
67	-1.7747602	4.36601089	0.82513307	h	1	0
68	10.43128727	1.79401002	-2.65564312	h	1	0
69	9.92022485	4.02724388	-0.1913264	h	1	0
70	8.99806204	0.84628857	0.22760727	h	1	0
71	8.18392323	-1.21688095	-9.67999115	h	1	0
72	5.49423499	0.01822695	-11.21852547	h	1	0
73	12.40128053	0.75601463	-10.18091487	h	1	0
74	15.26669673	3.13233524	-13.01075774	h	1	0
75	13.5057506	5.68606623	-16.5457556	h	1	0
76	8.8459252	5.82694729	-17.21658383	h	1	0
77	6.00081577	3.44790133	-14.40060576	h	1	0
78	4.62760171	-1.22839522	-6.60143706	h	1	0

1b-4

Center Number	Coordinates (Angstroms)			Atom	Atomic Number	Atomic Type
	X	Y	Z			
1	2.33431636	3.12010189	-8.20409935	o	8	0
2	4.49192505	3.98317754	-8.32264082	c	6	0
3	4.90334054	6.47182217	-8.87864748	n	7	0

4	7.36311139	7.72845536	-8.95754121	c	6	0
5	2.66844304	8.1292181	-8.97152969	c	6	0
6	0.9586395	7.77760699	-11.30656744	c	6	0
7	2.1655586	8.44408567	-13.81545069	c	6	0
8	2.20511991	6.67881265	-15.78926771	c	6	0
9	3.21262544	7.29286449	-18.15235727	c	6	0
10	4.21670179	9.69416412	-18.5797589	c	6	0
11	4.18611054	11.47754959	-16.63249513	c	6	0
12	3.16060067	10.85882847	-14.28366969	c	6	0
13	1.08005789	7.98128842	-6.53234112	c	6	0
14	2.56116651	7.70679288	-4.46952311	o	8	0
15	1.30638304	7.47304152	-2.00925851	c	6	0
16	2.19187968	9.65449672	-0.33653464	c	6	0
17	1.36155478	12.26025553	-1.31157034	c	6	0
18	1.98392291	14.45559987	0.48797289	c	6	0
19	4.816228	14.95098006	0.88158688	c	6	0
20	5.33187348	17.25280357	2.56279727	c	6	0
21	1.82137455	4.76433851	-1.01579925	c	6	0
22	0.05787102	4.07739794	1.18509741	c	6	0
23	4.59073845	4.42574241	-0.21642189	c	6	0
24	6.48564695	3.45464427	-2.1960048	c	6	0
25	9.13345312	3.09310553	-1.06039479	c	6	0
26	5.29666334	5.01891047	1.88894249	o	8	0
27	-1.18721362	8.27249845	-6.48479917	o	8	0
28	6.74200865	2.18509845	-7.73476941	c	6	0
29	7.53796951	0.63391218	-10.09954152	c	6	0

30	9.18158651	2.0577358	-11.95749968	c	6	0
31	8.14350922	3.30469689	-14.04840216	c	6	0
32	9.67740583	4.66941004	-15.70646085	c	6	0
33	12.28167128	4.7921127	-15.30517089	c	6	0
34	13.34410181	3.54250131	-13.23627605	c	6	0
35	11.80398076	2.18684124	-11.57952891	c	6	0
36	5.98257382	0.40935894	-5.7674146	n	7	0
37	5.50518732	0.95415269	-3.27237696	c	6	0
38	4.42807605	-0.57279832	-1.90276525	o	8	0
39	3.41846082	10.0613226	-8.98917206	h	1	0
40	-0.72338531	7.63402138	-2.35799104	h	1	0
41	1.41671915	3.50641796	-2.60590654	h	1	0
42	6.53542886	4.91124785	-3.67073682	h	1	0
43	8.38858871	3.22359928	-7.0512431	h	1	0
44	8.88515367	6.37102326	-9.2116119	h	1	0
45	7.42274813	9.00347481	-10.58250711	h	1	0
46	7.69463877	8.82541337	-7.2276753	h	1	0
47	-0.70716164	8.96525795	-10.99026479	h	1	0
48	0.29099445	5.82682904	-11.31972886	h	1	0
49	1.40575085	4.81130665	-15.47660165	h	1	0
50	3.20054697	5.89707298	-19.65915134	h	1	0
51	4.99612541	10.18090413	-20.41584361	h	1	0
52	4.94188476	13.36054766	-16.95042088	h	1	0
53	3.10624911	12.2900401	-12.80767374	h	1	0
54	1.41550658	9.34760114	1.56131802	h	1	0
55	4.24899247	9.57333841	-0.12985743	h	1	0

56	-0.6843377	12.23119532	-1.65486172	h	1	0
57	2.24818594	12.61757277	-3.15433986	h	1	0
58	1.08954547	14.10692772	2.3316749	h	1	0
59	1.10444634	16.18442548	-0.25283275	h	1	0
60	5.72107312	15.2284281	-0.96831647	h	1	0
61	5.71773603	13.28203704	1.71919864	h	1	0
62	7.36176491	17.56929753	2.8137826	h	1	0
63	4.52169818	18.97542396	1.74463037	h	1	0
64	4.49858162	17.00616577	4.443414	h	1	0
65	0.50166049	5.17860004	2.87430687	h	1	0
66	0.28738593	2.07866045	1.65558069	h	1	0
67	-1.91890407	4.399022	0.66607098	h	1	0
68	10.48921481	2.5130063	-2.51113433	h	1	0
69	9.7884624	4.84575736	-0.18977228	h	1	0
70	9.07668805	1.63925356	0.40576295	h	1	0
71	8.57058367	-1.01636181	-9.39405573	h	1	0
72	5.81902399	-0.0638751	-11.02211361	h	1	0
73	6.11900124	3.229677	-14.37723872	h	1	0
74	8.82475812	5.64224843	-17.29987822	h	1	0
75	13.47810637	5.84535741	-16.59926493	h	1	0
76	15.37158219	3.61354158	-12.9181325	h	1	0
77	12.64542525	1.2023622	-9.98222447	h	1	0
78	4.97881991	-1.11173583	-6.35951362	h	1	0

1b-5

Center Number	Coordinates (Angstroms)			Atom	Atomic Number	Atomic Type
	X	Y	Z			

1	2.22179094	3.71843569	-7.92919614	o	8	0
2	4.49577639	4.14017402	-8.18388675	c	6	0
3	5.36580367	6.52952579	-8.64083439	n	7	0
4	8.01932968	7.27266021	-8.85419074	c	6	0
5	3.51719528	8.60166372	-8.45947492	c	6	0
6	1.59401984	8.746686	-10.64628534	c	6	0
7	2.71660136	9.2944451	-13.22207941	c	6	0
8	2.2429512	7.6679828	-15.2573696	c	6	0
9	3.17240359	8.19420317	-17.67305174	c	6	0
10	4.61141678	10.36450578	-18.09339109	c	6	0
11	5.0947494	12.00875405	-16.08454622	c	6	0
12	4.14712306	11.48148092	-13.68109099	c	6	0
13	2.12905243	8.62896348	-5.89645867	c	6	0
14	3.65682937	7.88979058	-3.99207662	o	8	0
15	2.56839099	7.75159269	-1.44509552	c	6	0
16	4.02791279	9.57750397	0.25381436	c	6	0
17	3.76683472	12.38403377	-0.45696447	c	6	0
18	1.09489142	13.48104258	-0.18653175	c	6	0
19	0.98952482	16.33881167	-0.67093793	c	6	0
20	-1.67467643	17.44011784	-0.40785009	c	6	0
21	2.57850532	4.93317373	-0.63687421	c	6	0
22	0.87462999	4.47798229	1.66923606	c	6	0
23	5.2641172	3.97491385	-0.09465204	c	6	0
24	6.76904029	2.78972277	-2.2814285	c	6	0
25	9.35902447	1.81823153	-1.39904566	c	6	0
26	6.22337922	4.24664028	1.97586485	o	8	0

27	-0.00734837	9.40622795	-5.6350383	o	8	0
28	6.36856783	1.89477751	-7.88230077	c	6	0
29	6.67440857	0.38680218	-10.38481414	c	6	0
30	8.44552709	1.58223413	-12.28581323	c	6	0
31	7.54293589	3.13665204	-14.22815669	c	6	0
32	9.20753985	4.28413032	-15.92424389	c	6	0
33	11.80529219	3.87819199	-15.71108479	c	6	0
34	12.72966639	2.31527623	-13.7943712	c	6	0
35	11.06075652	1.17856299	-12.09830136	c	6	0
36	5.39852027	0.17065864	-5.96267215	n	7	0
37	5.21593746	0.62432721	-3.41111353	c	6	0
38	3.93899214	-0.74326623	-2.04466775	o	8	0
39	4.64194413	10.34341342	-8.45942972	h	1	0
40	0.59603414	8.33275255	-1.60790915	h	1	0
41	1.80336951	3.89488433	-2.24762979	h	1	0
42	7.01283738	4.30969255	-3.66919731	h	1	0
43	8.23276299	2.5356647	-7.27488642	h	1	0
44	9.21108636	5.65967603	-9.30269752	h	1	0
45	8.22482871	8.61855222	-10.40888556	h	1	0
46	8.68802702	8.15917951	-7.10128995	h	1	0
47	0.24644793	10.23194035	-10.13535491	h	1	0
48	0.5331497	6.97875948	-10.68068745	h	1	0
49	1.1031596	5.98562863	-14.9483225	h	1	0
50	2.76020292	6.91304547	-19.22469495	h	1	0
51	5.33163312	10.78436046	-19.96970066	h	1	0
52	6.19200514	13.71681043	-16.39511753	h	1	0

53	4.50141424	12.81065724	-12.15234409	h	1	0
54	3.36795207	9.29329667	2.19777289	h	1	0
55	6.02959096	9.04890339	0.25329943	h	1	0
56	4.43824381	12.66793972	-2.40130519	h	1	0
57	5.06353423	13.46468444	0.75089693	h	1	0
58	0.38243772	13.08748849	1.72700484	h	1	0
59	-0.19543922	12.5320853	-1.50350005	h	1	0
60	1.71458102	16.73379543	-2.57702403	h	1	0
61	2.27440182	17.30733264	0.64260673	h	1	0
62	-1.69173183	19.47892356	-0.76533606	h	1	0
63	-2.98482937	16.55421024	-1.74548253	h	1	0
64	-2.42719314	17.13101908	1.49794263	h	1	0
65	1.66763331	5.33975416	3.36913303	h	1	0
66	0.70518735	2.44750153	2.01164321	h	1	0
67	-1.02052497	5.24089885	1.34144219	h	1	0
68	10.45832052	1.08801032	-2.99225237	h	1	0
69	10.42212062	3.33310673	-0.48546844	h	1	0
70	9.10776106	0.29977804	-0.02252866	h	1	0
71	7.3945314	-1.48067686	-9.85522727	h	1	0
72	4.79259741	0.10998161	-11.20649865	h	1	0
73	5.52655802	3.47293588	-14.40942658	h	1	0
74	8.46426611	5.50268623	-17.39914285	h	1	0
75	13.10156799	4.76545114	-17.03314783	h	1	0
76	14.74721783	1.97164167	-13.62673261	h	1	0
77	11.79111112	-0.04934019	-10.61921165	h	1	0
78	4.0598288	-1.06657426	-6.55367619	h	1	0

1b-6

Center Number	Coordinates (Angstroms)			Atom	Atomic Number	Atomic Type
	X	Y	Z			
1	2.5095653	3.8522464	-8.17308874	o	8	0
2	4.44039721	5.02772827	-8.71529063	c	6	0
3	4.38645146	7.57502089	-9.13900772	n	7	0
4	6.56873335	9.14556689	-9.76934189	c	6	0
5	1.9803119	8.88480038	-8.67313291	c	6	0
6	-0.00353939	8.47640106	-10.77691705	c	6	0
7	0.91432562	9.33212638	-13.34620269	c	6	0
8	1.84638845	7.58611581	-15.1078416	c	6	0
9	2.73991359	8.37734085	-17.46247543	c	6	0
10	2.71902791	10.93840911	-18.09467992	c	6	0
11	1.78645924	12.699239	-16.36232741	c	6	0
12	0.89017216	11.89881111	-14.01421839	c	6	0
13	0.8615397	8.25659968	-6.06120371	c	6	0
14	2.68856216	8.05658946	-4.28968852	o	8	0
15	1.93505164	7.28476597	-1.73777547	c	6	0
16	2.65219877	9.39828977	0.09388901	c	6	0
17	1.18048085	11.86028753	-0.33654856	c	6	0
18	1.87693489	13.92146776	1.58326264	c	6	0
19	0.37371846	16.39328996	1.30388086	c	6	0
20	0.87745177	17.83672512	-1.15912525	c	6	0
21	3.12985333	4.65266983	-1.2400542	c	6	0
22	1.97498841	3.37457708	1.09646762	c	6	0
23	6.0141312	4.82219437	-0.95627483	c	6	0

24	7.65415268	4.39379742	-3.31746412	c	6	0
25	10.48838953	4.5225225	-2.69865057	c	6	0
26	6.97504198	5.38994573	1.05291351	o	8	0
27	-1.38532705	8.15365769	-5.65158251	o	8	0
28	6.98962905	3.59393282	-8.88800687	c	6	0
29	7.29218505	2.49658337	-11.58819302	c	6	0
30	9.77915821	1.15271429	-12.0283365	c	6	0
31	11.99331776	2.53595273	-12.48234996	c	6	0
32	14.3004867	1.31551608	-12.8496559	c	6	0
33	14.42871416	-1.32023847	-12.77533719	c	6	0
34	12.23619222	-2.71968227	-12.34039879	c	6	0
35	9.93116833	-1.4891354	-11.97371158	c	6	0
36	7.05202749	1.56154426	-7.0413254	n	7	0
37	7.00665252	1.81203948	-4.45707228	c	6	0
38	6.57913205	-0.00580118	-3.08601583	o	8	0
39	2.44550149	10.90384169	-8.59485768	h	1	0
40	-0.11699638	7.0446773	-1.76637523	h	1	0
41	2.67875066	3.51936961	-2.90991901	h	1	0
42	7.14033624	5.91999264	-4.62156047	h	1	0
43	8.58131736	4.85462459	-8.51102955	h	1	0
44	8.0386914	8.04176676	-10.69506696	h	1	0
45	5.98234632	10.60818977	-11.10368101	h	1	0
46	7.36978065	10.05691671	-8.0855895	h	1	0
47	-1.70229232	9.51486785	-10.22016252	h	1	0
48	-0.50119928	6.47408325	-10.80025418	h	1	0
49	1.84326359	5.58667039	-14.63395552	h	1	0

50	3.44019409	6.99122428	-18.80652948	h	1	0
51	3.40708619	11.5574561	-19.92731702	h	1	0
52	1.74104045	14.69459999	-16.8462727	h	1	0
53	0.14178258	13.28093643	-12.68788438	h	1	0
54	2.30874385	8.71156225	2.01917232	h	1	0
55	4.68858608	9.75957823	-0.02431976	h	1	0
56	-0.85843639	11.47914818	-0.23122326	h	1	0
57	1.54222199	12.53767495	-2.26142788	h	1	0
58	3.90858883	14.33661904	1.43895395	h	1	0
59	1.5837193	13.17546836	3.49885791	h	1	0
60	0.82775809	17.62654752	2.90876062	h	1	0
61	-1.65511256	15.97793422	1.45749114	h	1	0
62	-0.16322819	19.6252152	-1.19882279	h	1	0
63	2.89228557	18.27880688	-1.36475719	h	1	0
64	0.3012574	16.75004334	-2.82367063	h	1	0
65	2.54568004	4.33530926	2.83227676	h	1	0
66	2.6338965	1.41941329	1.20895983	h	1	0
67	-0.09026243	3.35787084	0.97696276	h	1	0
68	11.62806226	4.33690558	-4.4147367	h	1	0
69	10.94255137	6.31220323	-1.77576926	h	1	0
70	10.99417031	2.99498129	-1.40475064	h	1	0
71	5.69993603	1.21322588	-11.92314253	h	1	0
72	7.07511192	4.05587637	-12.93603048	h	1	0
73	11.90965378	4.58947464	-12.56513174	h	1	0
74	15.99490689	2.41968547	-13.20444947	h	1	0
75	16.22310619	-2.27475265	-13.06392882	h	1	0

76	12.31534704	-4.77056264	-12.28940325	h	1	0
77	8.22924831	-2.5972886	-11.65778809	h	1	0
78	6.37957207	-0.1389158	-7.60663339	h	1	0

1b-7

Center Number	Coordinates (Angstroms)			Atom	Atomic Number	Atomic Type
	X	Y	Z			
1	2.3139252	3.26851345	-8.1903646	o	8	0
2	4.51115864	4.01093824	-8.37551901	c	6	0
3	5.04334139	6.46358359	-8.98684193	n	7	0
4	7.56599309	7.58084914	-9.14300936	c	6	0
5	2.90104063	8.24018139	-9.05350948	c	6	0
6	1.11015628	7.94354427	-11.33423236	c	6	0
7	2.28944426	8.46823554	-13.88942996	c	6	0
8	2.15409344	6.65788376	-15.81747874	c	6	0
9	3.1349198	7.14436232	-18.2210673	c	6	0
10	4.28743498	9.46032629	-18.73628416	c	6	0
11	4.43224563	11.28755887	-16.83554688	c	6	0
12	3.43296366	10.7971644	-14.44531188	c	6	0
13	1.37480597	8.22364243	-6.57062709	c	6	0
14	2.89709649	7.91174823	-4.54297554	o	8	0
15	1.7027471	7.79408592	-2.04506814	c	6	0
16	2.76329922	9.94556809	-0.43685518	c	6	0
17	2.03154401	12.57901767	-1.41517126	c	6	0
18	3.10900863	14.77240238	0.15739793	c	6	0
19	2.05164455	14.97806412	2.85217248	c	6	0
20	3.06082296	17.28506904	4.28302745	c	6	0

21	2.08580634	5.07646156	-1.01578735	c	6	0
22	0.34235209	4.52849562	1.23929526	c	6	0
23	4.85124777	4.5923941	-0.27892702	c	6	0
24	6.63537123	3.48325563	-2.28874358	c	6	0
25	9.28819407	2.99180728	-1.21566729	c	6	0
26	5.64398515	5.17668825	1.79777654	o	8	0
27	-0.87201401	8.63345921	-6.46822473	o	8	0
28	6.67455272	2.10323946	-7.80711837	c	6	0
29	7.32588968	0.46840953	-10.15965512	c	6	0
30	8.99661297	1.76685725	-12.0840846	c	6	0
31	7.97463098	3.0283603	-14.17430353	c	6	0
32	9.53738964	4.27797245	-15.89505189	c	6	0
33	12.15357995	4.26883202	-15.55781875	c	6	0
34	13.19928126	3.00124468	-13.49132528	c	6	0
35	11.63085541	1.76177046	-11.77141075	c	6	0
36	5.86811178	0.40782957	-5.78818226	n	7	0
37	5.48586155	1.02485241	-3.29340572	c	6	0
38	4.35625652	-0.41143679	-1.86901694	o	8	0
39	3.75556388	10.1270881	-9.12795384	h	1	0
40	-0.32349842	8.06935576	-2.33947021	h	1	0
41	1.5695612	3.81757461	-2.57247007	h	1	0
42	6.72665708	4.91019981	-3.79001061	h	1	0
43	8.39150613	3.06156195	-7.1827833	h	1	0
44	9.00333155	6.13813686	-9.41882314	h	1	0
45	7.65349123	8.83225371	-10.78477949	h	1	0
46	8.00268033	8.67744956	-7.4361215	h	1	0

47	-0.4669622	9.2437325	-11.0062647	h	1	0
48	0.31930453	6.04009766	-11.28027483	h	1	0
49	1.23943253	4.85736429	-15.4353564	h	1	0
50	2.98617579	5.71666127	-19.69033048	h	1	0
51	5.04745879	9.84798973	-20.60383885	h	1	0
52	5.30417065	13.10642725	-17.22189386	h	1	0
53	3.51886519	12.26351389	-13.00575148	h	1	0
54	2.06814175	9.67863436	1.49446571	h	1	0
55	4.82654374	9.78278698	-0.32744515	h	1	0
56	-0.03772477	12.73792771	-1.4936767	h	1	0
57	2.69668521	12.77342936	-3.36940126	h	1	0
58	2.72386077	16.55151926	-0.84194648	h	1	0
59	5.17901353	14.60467236	0.24546167	h	1	0
60	2.50539619	13.26276124	3.92541509	h	1	0
61	-0.0214048	15.070821	2.76201888	h	1	0
62	2.27858009	17.39120038	6.19610299	h	1	0
63	5.1245496	17.2167016	4.45198892	h	1	0
64	2.57418856	19.04595854	3.30515306	h	1	0
65	0.899432	5.619908	2.90108169	h	1	0
66	0.4599644	2.52530626	1.73137126	h	1	0
67	-1.62381021	4.96699078	0.76766515	h	1	0
68	10.57238101	2.32007333	-2.69205711	h	1	0
69	10.05927391	4.71697752	-0.38645551	h	1	0
70	9.19088081	1.5619367	0.27167519	h	1	0
71	8.2845328	-1.22248993	-9.44689979	h	1	0
72	5.54926358	-0.15155212	-11.02715576	h	1	0

73	5.94148379	3.05555058	-14.45356173	h	1	0
74	8.69846948	5.26451862	-17.48732084	h	1	0
75	13.37168092	5.23470349	-16.89898614	h	1	0
76	15.23514095	2.96648592	-13.22496132	h	1	0
77	12.45794608	0.76465889	-10.17442152	h	1	0
78	4.7586216	-1.05900545	-6.32644185	h	1	0

1b-8

Center Number	Coordinates (Angstroms)			Atom	Atomic Number	Atomic Type
	X	Y	Z			
1	2.51822917	3.68829774	-8.22879093	o	8	0
2	4.36643022	4.97359263	-8.80804771	c	6	0
3	4.13573369	7.48994927	-9.34273275	n	7	0
4	6.20169929	9.18392929	-10.0419981	c	6	0
5	1.63644282	8.64252876	-8.95861141	c	6	0
6	-0.28668719	8.0011068	-11.06140458	c	6	0
7	0.59600934	8.81135474	-13.65740177	c	6	0
8	1.67708093	7.06546648	-15.33199714	c	6	0
9	2.5372875	7.81875741	-17.71142786	c	6	0
10	2.33292048	10.34133795	-18.45632231	c	6	0
11	1.25021463	12.10050438	-16.81176869	c	6	0
12	0.38732073	11.33736851	-14.43884962	c	6	0
13	0.53034827	8.04917406	-6.33318314	c	6	0
14	2.34161801	8.05742919	-4.53435262	o	8	0
15	1.60902979	7.32613118	-1.96368579	c	6	0
16	2.10771832	9.56128668	-0.20556023	c	6	0
17	0.4519538	11.87409856	-0.78505728	c	6	0

18	0.72041863	14.03388027	1.14064123	c	6	0
19	3.32977681	15.30349608	1.19990587	c	6	0
20	3.45783244	17.52417013	3.05522036	c	6	0
21	3.01277245	4.82718401	-1.34475067	c	6	0
22	1.94556144	3.55859533	1.03834817	c	6	0
23	5.87007729	5.24729219	-1.05001629	c	6	0
24	7.56858268	4.82856516	-3.37121975	c	6	0
25	10.37621511	5.20214216	-2.73200769	c	6	0
26	6.75740614	5.99983286	0.93253693	o	8	0
27	-1.70891243	7.80112419	-5.94442216	o	8	0
28	7.01248399	3.71870344	-8.89934061	c	6	0
29	7.41324376	2.51802653	-11.54231212	c	6	0
30	9.99553893	1.34265473	-11.90840756	c	6	0
31	12.10056956	2.86101369	-12.44250039	c	6	0
32	14.49440424	1.79924611	-12.7418529	c	6	0
33	14.82095252	-0.81050659	-12.51662534	c	6	0
34	12.73894361	-2.34460396	-12.0005685	c	6	0
35	10.34643379	-1.27277995	-11.70346724	c	6	0
36	7.20468183	1.78646051	-6.95676663	n	7	0
37	7.13174641	2.15352892	-4.38782997	c	6	0
38	6.84244142	0.37461824	-2.93185534	o	8	0
39	1.95567748	10.69128516	-8.96533093	h	1	0
40	-0.41454308	6.91075475	-2.01425591	h	1	0
41	2.67289674	3.59026434	-2.96643603	h	1	0
42	6.95867832	6.24690706	-4.7533659	h	1	0
43	8.5079713	5.10576941	-8.57631219	h	1	0

44	7.76624675	8.14431382	-10.88266046	h	1	0
45	5.52668219	10.51745158	-11.46681982	h	1	0
46	6.90396109	10.25051254	-8.40582115	h	1	0
47	-2.06350645	8.93390367	-10.56479537	h	1	0
48	-0.63663031	5.96819747	-11.00236223	h	1	0
49	1.81780558	5.09390112	-14.77046053	h	1	0
50	3.35476071	6.43217704	-18.98706081	h	1	0
51	2.99561414	10.93038401	-20.30807636	h	1	0
52	1.0619543	14.06398264	-17.38337965	h	1	0
53	-0.48008145	12.71493251	-13.18205806	h	1	0
54	1.74776001	8.92703215	1.73552314	h	1	0
55	4.11423678	10.0590179	-0.27953479	h	1	0
56	-1.53500384	11.28155192	-0.85974907	h	1	0
57	0.90896546	12.5884887	-2.6796491	h	1	0
58	0.26543989	13.32085151	3.03935731	h	1	0
59	-0.71066523	15.47749386	0.71732995	h	1	0
60	3.80489076	15.96846116	-0.71043579	h	1	0
61	4.7827978	13.91312473	1.70372765	h	1	0
62	5.33277725	18.40102573	3.06092431	h	1	0
63	2.07725673	18.99003789	2.56702287	h	1	0
64	3.0497384	16.90562319	4.99008264	h	1	0
65	2.42566783	4.63312692	2.73436636	h	1	0
66	2.75619845	1.66792663	1.23448198	h	1	0
67	-0.11107482	3.37359846	0.91173915	h	1	0
68	11.54978415	5.0241564	-4.42598047	h	1	0
69	10.68076627	7.06246999	-1.89105763	h	1	0

70	10.97887219	3.77937619	-1.36226382	h	1	0
71	5.92185909	1.1061472	-11.81987394	h	1	0
72	7.09128852	3.99056369	-12.96447467	h	1	0
73	11.86226273	4.89436645	-12.64228128	h	1	0
74	16.1016968	3.00610365	-13.16168329	h	1	0
75	16.68336988	-1.64137446	-12.7514994	h	1	0
76	12.97264216	-4.37741234	-11.83192428	h	1	0
77	8.73195293	-2.48744947	-11.32469219	h	1	0
78	6.67146359	0.01389333	-7.44335615	h	1	0

1b-9

Center Number	Coordinates (Angstroms)			Atom	Atomic Number	Atomic Type
	X	Y	Z			
1	2.33563439	3.14351683	-8.17251307	o	8	0
2	4.48605932	4.02845025	-8.25343973	c	6	0
3	4.88066968	6.52828637	-8.77091158	n	7	0
4	7.32980779	7.80788535	-8.78998274	c	6	0
5	2.63107766	8.16577077	-8.86272342	c	6	0
6	0.94776667	7.8313427	-11.21923114	c	6	0
7	2.1736442	8.54641381	-13.70558323	c	6	0
8	2.24457056	6.81322174	-15.70688028	c	6	0
9	3.27048979	7.47206216	-18.04987327	c	6	0
10	4.26138331	9.88687512	-18.42929819	c	6	0
11	4.19929324	11.63868041	-16.45433107	c	6	0
12	3.15602296	10.97514644	-14.12560146	c	6	0
13	1.02204378	7.96905999	-6.44060743	c	6	0
14	2.48826858	7.67957726	-4.36888353	o	8	0

15	1.21643886	7.40608586	-1.92129813	c	6	0
16	2.08185481	9.57038451	-0.21593835	c	6	0
17	1.22376247	12.18066493	-1.15075352	c	6	0
18	1.87641713	14.36049692	0.66090616	c	6	0
19	4.71003611	14.81631877	1.13735325	c	6	0
20	6.22236544	15.51815565	-1.23225902	c	6	0
21	1.7383734	4.68732565	-0.95912752	c	6	0
22	-0.0426558	3.96134543	1.21509173	c	6	0
23	4.5018167	4.35575492	-0.13632076	c	6	0
24	6.42386122	3.42667976	-2.11012831	c	6	0
25	9.06207383	3.06548257	-0.95230426	c	6	0
26	5.18196182	4.92394081	1.98435849	o	8	0
27	-1.24811147	8.23803354	-6.40877607	o	8	0
28	6.74623491	2.24386134	-7.66457987	c	6	0
29	7.58442654	0.73385592	-10.04139651	c	6	0
30	9.22941683	2.20086219	-11.86415016	c	6	0
31	8.19711399	3.46456527	-13.94789649	c	6	0
32	9.73127968	4.86818868	-15.57285	c	6	0
33	12.33018593	5.01366918	-15.14545897	c	6	0
34	13.38720376	3.74687963	-13.08431282	c	6	0
35	11.84661935	2.35327887	-11.45982456	c	6	0
36	5.98022778	0.43300832	-5.73220683	n	7	0
37	5.47239107	0.93657894	-3.23477219	c	6	0
38	4.39194934	-0.61858182	-1.89976231	o	8	0
39	3.36251351	10.10492439	-8.84635691	h	1	0
40	-0.81150781	7.56135809	-2.28281807	h	1	0

41	1.35727451	3.44850869	-2.5698853	h	1	0
42	6.47927716	4.90634271	-3.56160696	h	1	0
43	8.37458427	3.28806007	-6.94704243	h	1	0
44	8.86538813	6.47227257	-9.07637715	h	1	0
45	7.38927973	9.1339304	-10.37355024	h	1	0
46	7.6399333	8.85369248	-7.0245241	h	1	0
47	-0.73261028	8.99829704	-10.90251313	h	1	0
48	0.29945695	5.87464525	-11.26768734	h	1	0
49	1.45560558	4.93543527	-15.43180439	h	1	0
50	3.28349583	6.10037367	-19.57858006	h	1	0
51	5.05493781	10.40827658	-20.24972133	h	1	0
52	4.94394806	13.53200487	-16.73489396	h	1	0
53	3.07816086	12.38200184	-12.62745744	h	1	0
54	1.31860942	9.22720258	1.68086858	h	1	0
55	4.14105446	9.50566928	-0.01882524	h	1	0
56	-0.83068669	12.15408522	-1.43846728	h	1	0
57	2.04992402	12.54719928	-3.01681716	h	1	0
58	0.94314366	14.01660311	2.48394715	h	1	0
59	1.04028812	16.10694335	-0.08901595	h	1	0
60	5.55590789	13.1446534	2.02674991	h	1	0
61	4.89314298	16.34431873	2.52854421	h	1	0
62	8.19005978	15.95208834	-0.75907927	h	1	0
63	6.24705592	13.97917338	-2.61789432	h	1	0
64	5.41888355	17.18493438	-2.16604556	h	1	0
65	0.39474426	5.02595768	2.9292232	h	1	0
66	0.17643405	1.95238993	1.64548248	h	1	0

67	-2.01486903	4.30127102	0.69051289	h	1	0
68	10.44264925	2.54393875	-2.40162659	h	1	0
69	9.68298502	4.80070544	-0.02372035	h	1	0
70	9.00611589	1.56956266	0.47102365	h	1	0
71	8.62907573	-0.91364275	-9.34717283	h	1	0
72	5.88286914	0.02874712	-10.99034274	h	1	0
73	6.17671668	3.37173747	-14.29699889	h	1	0
74	8.8827893	5.85305308	-17.16104283	h	1	0
75	13.52657447	6.09887809	-16.41287883	h	1	0
76	15.41113016	3.83304048	-12.74794865	h	1	0
77	12.68370821	1.35806504	-9.86694015	h	1	0
78	4.99531147	-1.08690701	-6.35784666	h	1	0

1b-10

Center Number	Coordinates (Angstroms)			Atom	Atomic Number	Atomic Type
	X	Y	Z			
1	2.52182108	3.95349455	-8.13512748	o	8	0
2	4.47197596	5.06469378	-8.73994065	c	6	0
3	4.4787556	7.60427404	-9.21179581	n	7	0
4	6.68726441	9.10092032	-9.92551377	c	6	0
5	2.12005448	8.98960173	-8.72286141	c	6	0
6	0.08297906	8.59717294	-10.77844506	c	6	0
7	0.97031355	9.38022688	-13.38134443	c	6	0
8	1.82465625	7.57826594	-15.12603921	c	6	0
9	2.69047637	8.30189315	-17.51250254	c	6	0
10	2.71951413	10.85016779	-18.19421082	c	6	0
11	1.86424377	12.66630456	-16.47920162	c	6	0

12	0.99522418	11.933401	-14.0990426	c	6	0
13	1.03789023	8.44368274	-6.07721555	c	6	0
14	2.89365013	8.22220304	-4.3389388	o	8	0
15	2.16924644	7.51772913	-1.75888607	c	6	0
16	2.93828263	9.65984382	0.01750597	c	6	0
17	1.52045354	12.14239935	-0.47146935	c	6	0
18	2.23836265	14.22262951	1.41992038	c	6	0
19	0.98722126	16.79681658	0.91830883	c	6	0
20	-1.89371508	16.81525148	1.23655242	c	6	0
21	3.33689582	4.8814504	-1.22410187	c	6	0
22	2.22649531	3.68179117	1.17499186	c	6	0
23	6.23018584	5.01121096	-1.02179857	c	6	0
24	7.79985993	4.48055489	-3.40979062	c	6	0
25	10.65105313	4.56007822	-2.86487197	c	6	0
26	7.25375782	5.62634759	0.94205502	o	8	0
27	-1.20229531	8.41508476	-5.62106786	o	8	0
28	6.97675371	3.55730999	-8.93732134	c	6	0
29	7.17945281	2.3844972	-11.61458868	c	6	0
30	9.6050471	0.93823684	-12.07396878	c	6	0
31	11.86941359	2.22635617	-12.55953684	c	6	0
32	14.11957027	0.90935166	-12.9461653	c	6	0
33	14.13931195	-1.72928291	-12.86031445	c	6	0
34	11.8958776	-3.03444609	-12.39417981	c	6	0
35	9.6481213	-1.70735252	-12.00741659	c	6	0
36	7.03122052	1.57150778	-7.04069419	n	7	0
37	7.06658157	1.88587627	-4.46403253	c	6	0

38	6.64114012	0.11244653	-3.03510063	o	8	0
39	2.64247637	10.99599067	-8.69327161	h	1	0
40	0.11396185	7.30438246	-1.75171636	h	1	0
41	2.8236287	3.71305502	-2.85122027	h	1	0
42	7.28756434	5.983764	-4.7406414	h	1	0
43	8.61129378	4.78251396	-8.63277071	h	1	0
44	8.10507648	7.93809441	-10.86043744	h	1	0
45	6.1095568	10.55046808	-11.27775169	h	1	0
46	7.55140412	10.02547328	-8.28065679	h	1	0
47	-1.57530332	9.69190299	-10.20764617	h	1	0
48	-0.46862417	6.60907903	-10.75391155	h	1	0
49	1.78250857	5.58902124	-14.61286465	h	1	0
50	3.33047501	6.87353727	-18.84235304	h	1	0
51	3.38640681	11.41694896	-20.05143442	h	1	0
52	1.85777659	14.65252443	-17.00135819	h	1	0
53	0.3066096	13.35842785	-12.78588299	h	1	0
54	2.57814009	9.02843772	1.95871279	h	1	0
55	4.98207824	9.97225484	-0.10789534	h	1	0
56	-0.52166426	11.78891965	-0.40817172	h	1	0
57	1.9334261	12.80243821	-2.39581358	h	1	0
58	4.30069567	14.4650831	1.39521304	h	1	0
59	1.76148916	13.59527618	3.34323858	h	1	0
60	1.47505317	17.42825637	-0.99964638	h	1	0
61	1.81507947	18.19622426	2.2071228	h	1	0
62	-2.66112772	18.71706972	0.95497621	h	1	0
63	-2.83113796	15.56360056	-0.1187005	h	1	0

64	-2.43622471	16.1963678	3.13840791	h	1	0
65	2.86488165	4.67326936	2.86939289	h	1	0
66	2.85226924	1.71758302	1.3161971	h	1	0
67	0.15862936	3.7015776	1.11481007	h	1	0
68	11.74238278	4.3029978	-4.6030971	h	1	0
69	11.16856414	6.36236514	-2.00218164	h	1	0
70	11.15486828	3.0555057	-1.54353441	h	1	0
71	5.53496579	1.1524272	-11.88320075	h	1	0
72	6.98865599	3.91815383	-12.99555942	h	1	0
73	11.87096469	4.28117335	-12.65049933	h	1	0
74	15.85448601	1.94052104	-13.32434375	h	1	0
75	15.88931272	-2.7586368	-13.16363507	h	1	0
76	11.89027579	-5.08656675	-12.33402993	h	1	0
77	7.90521975	-2.74219	-11.66618266	h	1	0
78	6.31647085	-0.13003728	-7.54765178	h	1	0

1b-11

Center Number	Coordinates (Angstroms)			Atom	Atomic Number	Atomic Type
	X	Y	Z			
1	2.57711438	4.33792636	-7.87967243	o	8	0
2	4.58590707	5.27098265	-8.58643734	c	6	0
3	4.79118154	7.79958761	-9.07229389	n	7	0
4	7.083647	9.10248983	-9.89462269	c	6	0
5	2.58430727	9.38234796	-8.48644197	c	6	0
6	0.42852048	9.15715854	-10.44431743	c	6	0
7	1.25777403	9.84644605	-13.09221757	c	6	0
8	1.88099918	7.96759504	-14.85345358	c	6	0

9	2.68999965	8.60018425	-17.2850789	c	6	0
10	2.8924713	11.13246057	-17.99646106	c	6	0
11	2.26859164	13.02522893	-16.26488236	c	6	0
12	1.45573044	12.38360097	-13.83903658	c	6	0
13	1.58242884	8.9363873	-5.79052145	c	6	0
14	3.48755118	8.57039458	-4.13522824	o	8	0
15	2.82854141	7.92938674	-1.51960457	c	6	0
16	3.87984992	9.99279359	0.20893506	c	6	0
17	2.75840177	12.63932994	-0.21937124	c	6	0
18	-0.07049556	12.89892227	0.37519331	c	6	0
19	-1.02559484	15.62711747	0.1458645	c	6	0
20	-3.84981713	15.89957805	0.72475886	c	6	0
21	3.79033047	5.20153421	-1.02867153	c	6	0
22	2.69665683	4.10611021	1.42791413	c	6	0
23	6.69012758	5.0704863	-0.96727252	c	6	0
24	8.08894358	4.40325946	-3.42623598	c	6	0
25	10.95989883	4.23764831	-3.02143015	c	6	0
26	7.85983872	5.5865696	0.94273264	o	8	0
27	-0.63238299	9.09321037	-5.23814126	o	8	0
28	6.93320966	3.54647437	-8.90047395	c	6	0
29	6.89617675	2.36035973	-11.578418	c	6	0
30	9.15134417	0.69402761	-12.14764525	c	6	0
31	11.51014249	1.7642391	-12.70763816	c	6	0
32	13.60468587	0.24136599	-13.19801338	c	6	0
33	13.37060954	-2.38775756	-13.14364465	c	6	0
34	11.03048169	-3.47656262	-12.6041236	c	6	0

35	8.93937906	-1.94377542	-12.11228835	c	6	0
36	6.90464174	1.56429224	-6.99945562	n	7	0
37	7.08500766	1.87932402	-4.42905072	c	6	0
38	6.57450926	0.15244334	-2.9714774	o	8	0
39	3.27581595	11.33708182	-8.49396896	h	1	0
40	0.76804	7.87989074	-1.41689137	h	1	0
41	3.09706403	4.0807191	-2.62231099	h	1	0
42	7.64325091	5.94302156	-4.73900944	h	1	0
43	8.68363365	4.6207522	-8.68360389	h	1	0
44	8.36807162	7.81444608	-10.8578488	h	1	0
45	6.57364549	10.57305521	-11.25168025	h	1	0
46	8.07869351	9.97948329	-8.29867997	h	1	0
47	-1.10132124	10.39423208	-9.80925541	h	1	0
48	-0.29071941	7.22432258	-10.37628441	h	1	0
49	1.70103779	5.9923588	-14.31648722	h	1	0
50	3.14952298	7.11459487	-18.62688269	h	1	0
51	3.51467861	11.6287731	-19.88905375	h	1	0
52	2.39812384	15.00131257	-16.8090157	h	1	0
53	0.94573739	13.86979291	-12.51207074	h	1	0
54	3.53600927	9.40740823	2.1671585	h	1	0
55	5.93694358	10.07155827	-0.00648673	h	1	0
56	3.10920111	13.22445933	-2.17999259	h	1	0
57	3.82004341	13.97285294	0.96548465	h	1	0
58	-0.4336611	12.2092969	2.30309316	h	1	0
59	-1.1730093	11.69401651	-0.90202464	h	1	0
60	-0.64514874	16.32144279	-1.77467148	h	1	0

61	0.06600291	16.84825289	1.4230738	h	1	0
62	-4.47420071	17.86482518	0.54427676	h	1	0
63	-4.9915428	14.75337684	-0.56878849	h	1	0
64	-4.28212592	15.27839566	2.65346671	h	1	0
65	3.50209194	5.0377112	3.0849253	h	1	0
66	3.1526278	2.09424252	1.54779853	h	1	0
67	0.63785154	4.30856314	1.46890437	h	1	0
68	11.93805127	3.88056592	-4.80895284	h	1	0
69	11.67274801	5.99136745	-2.19926465	h	1	0
70	11.39661013	2.70001288	-1.71430511	h	1	0
71	5.13367097	1.28623118	-11.7623584	h	1	0
72	6.78241695	3.90577021	-12.95508219	h	1	0
73	11.70928278	3.81048276	-12.77363108	h	1	0
74	15.41633511	1.10509838	-13.6316878	h	1	0
75	14.99928904	-3.57679889	-13.52757393	h	1	0
76	10.82714432	-5.51922907	-12.56776955	h	1	0
77	7.11861501	-2.81014632	-11.71188186	h	1	0
78	6.02495125	-0.07053409	-7.46476836	h	1	0

1b-12

Center Number	Coordinates (Angstroms)			Atom	Atomic Number	Atomic Type
	X	Y	Z			
1	2.51966412	3.82083178	-8.19952574	o	8	0
2	4.39938347	5.01424358	-8.86748471	c	6	0
3	4.24729548	7.51486565	-9.49524154	n	7	0
4	6.35605066	9.10473885	-10.30155577	c	6	0
5	1.80160314	8.77325389	-9.09816482	c	6	0

6	-0.1964459	8.12197877	-11.12656738	c	6	0
7	0.64742117	8.79859799	-13.77316437	c	6	0
8	1.6338706	6.95380123	-15.39952342	c	6	0
9	2.45775482	7.58349149	-17.82709319	c	6	0
10	2.31087693	10.07912705	-18.66980787	c	6	0
11	1.32259076	11.93592121	-17.07393882	c	6	0
12	0.49640764	11.29633835	-14.6520433	c	6	0
13	0.74372083	8.32448283	-6.42424913	c	6	0
14	2.6016107	8.34104421	-4.67304306	o	8	0
15	1.91443792	7.7472076	-2.05491549	c	6	0
16	2.58820218	10.01891219	-0.40476943	c	6	0
17	0.99613438	12.3750391	-0.98562479	c	6	0
18	1.6937383	14.70094512	0.61071391	c	6	0
19	1.13988086	14.44824969	3.44881395	c	6	0
20	1.72060632	16.87002256	4.92728565	c	6	0
21	3.20518821	5.20206281	-1.37566045	c	6	0
22	2.12264988	4.06742884	1.06762925	c	6	0
23	6.08477569	5.4907358	-1.14538531	c	6	0
24	7.71282806	4.92840411	-3.48674519	c	6	0
25	10.54739942	5.198922	-2.91819117	c	6	0
26	7.04694248	6.2502661	0.79885832	o	8	0
27	-1.49139845	8.17261241	-5.96920844	o	8	0
28	6.99634685	3.66090672	-8.96275031	c	6	0
29	7.31025273	2.36227358	-11.57126253	c	6	0
30	9.84843471	1.09612967	-11.94353499	c	6	0
31	11.98329505	2.52895041	-12.58453261	c	6	0

32	14.33876424	1.38632699	-12.89046031	c	6	0
33	14.59588572	-1.22051281	-12.56488403	c	6	0
34	12.48306776	-2.67051254	-11.94164233	c	6	0
35	10.1294281	-1.51787062	-11.6378293	c	6	0
36	7.1527794	1.78686092	-6.96120187	n	7	0
37	7.13939748	2.24362524	-4.40507644	c	6	0
38	6.79619742	0.52939053	-2.8849872	o	8	0
39	2.19427156	10.80708497	-9.19507837	h	1	0
40	-0.12873821	7.43866701	-2.02749102	h	1	0
41	2.77412797	3.92993852	-2.94762161	h	1	0
42	7.1335927	6.32676953	-4.90199946	h	1	0
43	8.54619282	5.00233657	-8.70881486	h	1	0
44	7.87052482	7.97611614	-11.11860034	h	1	0
45	5.70218162	10.39719897	-11.7734154	h	1	0
46	7.12138149	10.21730766	-8.72541463	h	1	0
47	-1.92519414	9.13870876	-10.6247055	h	1	0
48	-0.61727582	6.10721455	-10.97842728	h	1	0
49	1.7287536	5.00307898	-14.76101165	h	1	0
50	3.20240482	6.12219728	-19.06328911	h	1	0
51	2.94460697	10.57226576	-20.55939656	h	1	0
52	1.17919848	13.87962833	-17.72130106	h	1	0
53	-0.29673519	12.74961436	-13.43209143	h	1	0
54	2.33388542	9.45282001	1.56973539	h	1	0
55	4.60271174	10.45688637	-0.61714663	h	1	0
56	-1.01626807	11.93463227	-0.72102645	h	1	0
57	1.21932697	12.85435888	-2.99027346	h	1	0

58	0.65212434	16.34077183	-0.12346872	h	1	0
59	3.70834963	15.1388797	0.34435887	h	1	0
60	2.24163943	12.88446687	4.25015264	h	1	0
61	-0.85741307	13.93983309	3.70807519	h	1	0
62	1.31055392	16.64385873	6.94324933	h	1	0
63	3.71703393	17.39489006	4.7487191	h	1	0
64	0.59326476	18.4626601	4.23011695	h	1	0
65	2.68155586	5.17482586	2.71772907	h	1	0
66	2.84878095	2.1488714	1.31716054	h	1	0
67	0.05794031	3.97419568	0.98003689	h	1	0
68	11.67448445	4.91441399	-4.62911315	h	1	0
69	10.95108367	7.07041762	-2.14677744	h	1	0
70	11.11776719	3.79660177	-1.5140027	h	1	0
71	5.77113588	0.9892164	-11.77558147	h	1	0
72	7.00701988	3.79638877	-13.03579292	h	1	0
73	11.79827973	4.55842119	-12.86364953	h	1	0
74	15.96988477	2.52758237	-13.39354331	h	1	0
75	16.4280213	-2.11483919	-12.80536522	h	1	0
76	12.66244578	-4.70078664	-11.69461492	h	1	0
77	8.49052731	-2.66820055	-11.17318021	h	1	0
78	6.52482621	0.02683703	-7.37574754	h	1	0

1b-13

Center Number	Coordinates (Angstroms)			Atom	Atomic Number	Atomic Type
	X	Y	Z			
1	2.53297443	3.7058866	-8.19297033	o	8	0
2	4.38076349	5.00795518	-8.73470231	c	6	0

3	4.14494524	7.53185448	-9.23040907	n	7	0
4	6.21314886	9.24332273	-9.87989119	c	6	0
5	1.63695001	8.66769159	-8.85246962	c	6	0
6	-0.25781071	8.06013047	-10.99044837	c	6	0
7	0.649864	8.93356165	-13.55724169	c	6	0
8	1.74796148	7.22980937	-15.26359734	c	6	0
9	2.6325467	8.04207293	-17.61458349	c	6	0
10	2.43544343	10.5823498	-18.29859671	c	6	0
11	1.33555587	12.30022064	-16.62202028	c	6	0
12	0.44895601	11.4784002	-14.2776766	c	6	0
13	0.50224623	8.01818814	-6.25278126	c	6	0
14	2.2906432	8.00754049	-4.43113329	o	8	0
15	1.53198187	7.22004599	-1.8846271	c	6	0
16	1.97731527	9.42808668	-0.07864391	c	6	0
17	0.30442062	11.7312006	-0.64383691	c	6	0
18	0.51019549	13.85191388	1.33591951	c	6	0
19	3.13029748	15.0850753	1.61581143	c	6	0
20	4.0811259	16.4232128	-0.7723024	c	6	0
21	2.95817479	4.72739618	-1.29412579	c	6	0
22	1.88413612	3.40710363	1.05763237	c	6	0
23	5.80781666	5.17364848	-0.96670012	c	6	0
24	7.53350253	4.7977558	-3.27533677	c	6	0
25	10.33158565	5.18128262	-2.60130562	c	6	0
26	6.66803295	5.91362972	1.03243135	o	8	0
27	-1.73970581	7.74673855	-5.89697592	o	8	0
28	7.03287957	3.76599413	-8.82407475	c	6	0

29	7.45605861	2.60464671	-11.48100333	c	6	0
30	10.04206466	1.43628695	-11.84334248	c	6	0
31	12.15402817	2.96462624	-12.31861364	c	6	0
32	14.55054106	1.9078693	-12.61369619	c	6	0
33	14.87288285	-0.70669784	-12.44421396	c	6	0
34	12.78413248	-2.25032299	-11.98734908	c	6	0
35	10.3889737	-1.18334377	-11.69338994	c	6	0
36	7.22035867	1.80689649	-6.90809283	n	7	0
37	7.12561284	2.1348388	-4.33484634	c	6	0
38	6.8392467	0.33221066	-2.90761581	o	8	0
39	1.94792205	10.7172758	-8.81474425	h	1	0
40	-0.48524513	6.78068052	-1.97110183	h	1	0
41	2.64646217	3.51434696	-2.93928258	h	1	0
42	6.92848848	6.2318796	-4.64329995	h	1	0
43	8.52008998	5.15474009	-8.47167256	h	1	0
44	7.77738287	8.22639153	-10.74872007	h	1	0
45	5.54000634	10.61545919	-11.2681678	h	1	0
46	6.91540998	10.26441573	-8.21495393	h	1	0
47	-2.04651632	8.97007748	-10.49414302	h	1	0
48	-0.59472018	6.02412292	-10.98008922	h	1	0
49	1.88325345	5.24476246	-14.75007924	h	1	0
50	3.4639871	6.68768947	-18.91552589	h	1	0
51	3.11700943	11.21710173	-20.12826052	h	1	0
52	1.152407	14.27723179	-17.14673752	h	1	0
53	-0.42997065	12.82488941	-12.99540098	h	1	0
54	1.59709374	8.75464991	1.84534086	h	1	0

55	3.9793404	9.94939115	-0.11294628	h	1	0
56	-1.67560142	11.12142448	-0.75377327	h	1	0
57	0.77048711	12.47371142	-2.5225556	h	1	0
58	-0.06985678	13.09860535	3.18205499	h	1	0
59	-0.87088676	15.3282917	0.86140298	h	1	0
60	4.51991464	13.66606375	2.21281482	h	1	0
61	3.03332205	16.46211631	3.16450421	h	1	0
62	5.88913838	17.36991142	-0.42565884	h	1	0
63	4.36857244	15.09862866	-2.33748284	h	1	0
64	2.73158121	17.86106649	-1.41090971	h	1	0
65	2.34108744	4.45607034	2.77598435	h	1	0
66	2.71067322	1.52076972	1.22655537	h	1	0
67	-0.16975268	3.2057802	0.91176268	h	1	0
68	11.52388382	5.03696456	-4.28544486	h	1	0
69	10.61368425	7.03077967	-1.72926244	h	1	0
70	10.93022044	3.74218673	-1.24697581	h	1	0
71	5.96812717	1.19611259	-11.79115935	h	1	0
72	7.14434278	4.09795396	-12.88389336	h	1	0
73	11.91931011	5.00218384	-12.47516894	h	1	0
74	16.16350388	3.12258839	-12.98643828	h	1	0
75	16.73736402	-1.53361001	-12.6765892	h	1	0
76	13.01465128	-4.28664698	-11.86242249	h	1	0
77	8.76954231	-2.40471361	-11.36021063	h	1	0
78	6.71007918	0.03650822	-7.42588845	h	1	0

1b-14

Center Number	Coordinates (Angstroms)			Atom	Atomic Number	Atomic Type

	X	Y	Z			
1	2.23594536	3.65872422	-7.95092901	o	8	0
2	4.50170026	4.14706152	-8.15217258	c	6	0
3	5.31461643	6.56526691	-8.56158754	n	7	0
4	7.95187274	7.38060085	-8.70317217	c	6	0
5	3.40680525	8.58409097	-8.39199216	c	6	0
6	1.52545907	8.70376266	-10.61654914	c	6	0
7	2.68438724	9.31430102	-13.16204582	c	6	0
8	2.28722936	7.70719253	-15.2288718	c	6	0
9	3.24946253	8.28996326	-17.6185812	c	6	0
10	4.64532899	10.49855463	-17.98067915	c	6	0
11	5.05223731	12.12402329	-15.93995263	c	6	0
12	4.07182778	11.5401337	-13.56285645	c	6	0
13	1.96540054	8.54217752	-5.8585993	c	6	0
14	3.46735573	7.80748379	-3.93265042	o	8	0
15	2.32836384	7.60209438	-1.41187906	c	6	0
16	3.67187857	9.47035977	0.33703669	c	6	0
17	3.30749357	12.26992971	-0.35973115	c	6	0
18	0.5833265	13.240558	-0.10308026	c	6	0
19	0.29662985	16.1036113	-0.52116037	c	6	0
20	0.8936889	16.98097347	-3.21928864	c	6	0
21	2.43543076	4.77852084	-0.63123001	c	6	0
22	0.71659007	4.23315009	1.64410359	c	6	0
23	5.14741994	3.91738298	-0.05665413	c	6	0
24	6.72272499	2.79025173	-2.22496393	c	6	0
25	9.32211586	1.88274514	-1.30312739	c	6	0

26	6.07044268	4.22121264	2.02593752	o	8	0
27	-0.19314175	9.26875391	-5.63267203	o	8	0
28	6.43056944	1.95074326	-7.84089351	c	6	0
29	6.81933438	0.47990068	-10.35394508	c	6	0
30	8.59074745	1.744401	-12.20956756	c	6	0
31	11.21211166	1.40652744	-11.98142196	c	6	0
32	12.87937328	2.60729046	-13.63434745	c	6	0
33	11.94721335	4.16911795	-15.54812679	c	6	0
34	9.34381095	4.51050382	-15.8012062	c	6	0
35	7.68105839	3.29933566	-14.14813639	c	6	0
36	5.47650684	0.180326	-5.95580068	n	7	0
37	5.25075791	0.5937721	-3.40141568	c	6	0
38	3.99905852	-0.82904987	-2.06816108	o	8	0
39	4.4825435	10.35583442	-8.3474059	h	1	0
40	0.33785765	8.10341436	-1.61531458	h	1	0
41	1.72458332	3.72883761	-2.26412894	h	1	0
42	6.94998702	4.32976449	-3.59416588	h	1	0
43	8.26604574	2.63540178	-7.1955143	h	1	0
44	9.19167093	5.81266405	-9.18095684	h	1	0
45	8.14853613	8.77851693	-10.2122459	h	1	0
46	8.56900406	8.22973426	-6.91301164	h	1	0
47	0.12709201	10.14493023	-10.1159038	h	1	0
48	0.51500957	6.90776613	-10.69420794	h	1	0
49	1.18137198	5.99454385	-14.96588069	h	1	0
50	2.89633604	7.0224628	-19.19585904	h	1	0
51	5.39121931	10.96176864	-19.83663745	h	1	0

52	6.1150796	13.8613023	-16.20492438	h	1	0
53	4.3659463	12.85523304	-12.00933099	h	1	0
54	2.98056916	9.14037654	2.26299295	h	1	0
55	5.6932443	9.0246683	0.37686588	h	1	0
56	3.99839348	12.58189849	-2.28976086	h	1	0
57	4.54027168	13.39671161	0.87364749	h	1	0
58	-0.11263196	12.77091756	1.79723382	h	1	0
59	-0.64614451	12.24724451	-1.44539787	h	1	0
60	1.50854593	17.12252598	0.82384584	h	1	0
61	-1.6515386	16.64301316	-0.057441	h	1	0
62	0.51343809	19.00306187	-3.4479206	h	1	0
63	2.88073364	16.66899689	-3.70948812	h	1	0
64	-0.26203463	15.95916268	-4.60234426	h	1	0
65	1.46584094	5.08469252	3.36880963	h	1	0
66	0.59967013	2.19254825	1.94592866	h	1	0
67	-1.19507071	4.94959855	1.30742639	h	1	0
68	10.47839016	1.21732228	-2.88439675	h	1	0
69	10.31607495	3.41247202	-0.33839289	h	1	0
70	9.0887382	0.33084245	0.0390177	h	1	0
71	7.57990837	-1.37386535	-9.83270939	h	1	0
72	4.95995291	0.1629243	-11.21160702	h	1	0
73	11.94899249	0.17961338	-10.50474933	h	1	0
74	14.90210313	2.31460624	-13.43536926	h	1	0
75	13.24209194	5.10560022	-16.83720137	h	1	0
76	8.59414522	5.72763521	-17.27403678	h	1	0
77	5.65997374	3.58602675	-14.35990869	h	1	0

78	4.18423731	-1.08889639	-6.5810889	h	1	0
